# An Integrative Computational Approach Based on Expression Similarity Signatures to Identify Protein–Protein Interaction Networks in Female-Specific Cancers

**DOI:** 10.3389/fgene.2020.612521

**Published:** 2020-12-03

**Authors:** Katia Pane, Ornella Affinito, Mario Zanfardino, Rossana Castaldo, Mariarosaria Incoronato, Marco Salvatore, Monica Franzese

**Affiliations:** IRCCS SDN, Naples, Italy

**Keywords:** TCGA, breast cancer, ovarian cancer, endometrial cancer, bioinformatics, signaling pathway, molecular signatures, principal component analysis - PCA

## Abstract

Breast, ovarian, and endometrial cancers have a major impact on mortality in women. These tumors share hormone-dependent mechanisms involved in female-specific cancers which support tumor growth in a different manner. Integrated computational approaches may allow us to better detect genomic similarities between these different female-specific cancers, helping us to deliver more sophisticated diagnosis and precise treatments. Recently, several initiatives of The Cancer Genome Atlas (TCGA) have encouraged integrated analyses of multiple cancers rather than individual tumors. These studies revealed common genetic alterations (driver genes) even in clinically distinct entities such as breast, ovarian, and endometrial cancers. In this study, we aimed to identify expression similarity signatures by extracting common genes among TCGA breast (BRCA), ovarian (OV), and uterine corpus endometrial carcinoma (UCEC) cohorts and infer co-regulatory protein–protein interaction networks that might have a relationship with the estrogen signaling pathway. Thus, we carried out an unsupervised principal component analysis (PCA)-based computational approach, using RNA sequencing data of 2,015 female cancer and 148 normal samples, in order to simultaneously capture the data heterogeneity of intertumors. Firstly, we identified tumor-associated genes from gene expression profiles. Secondly, we investigated the signaling pathways and co-regulatory protein–protein interaction networks underlying these three cancers by leveraging the Ingenuity Pathway Analysis software. In detail, we discovered 1,643 expression similarity signatures (638 downregulated and 1,005 upregulated genes, with respect to normal phenotype), denoted as tumor-associated genes. Through functional genomic analyses, we assessed that these genes were involved in the regulation of cell-cycle-dependent mechanisms, including metaphase kinetochore formation and estrogen-dependent S-phase entry. Furthermore, we generated putative co-regulatory protein–protein interaction networks, based on upstream regulators such as the ERBB2/HER2 gene. Moreover, we provided an *ad-hoc* bioinformatics workflow with a manageable list of intertumor expression similarity signatures for the three female-specific cancers. The expression similarity signatures identified in this study might uncover potential estrogen-dependent molecular mechanisms promoting carcinogenesis.

## Introduction

Breast, ovarian, and endometrial cancers have a major impact on mortality in women worldwide (Ginsburg et al., 2017; deSantis et al., 2019). Nowadays, biomedical data integration represents an emerging frontier for precision oncology (Incoronato et al., 2017; Affinito et al., 2019; Zanfardino et al., 2019). Integrative analyses of female-specific and hormone-sensitive cancers may decipher still cryptic molecular features. In this scenario, The Cancer Genome Atlas (TCGA) may yield an unprecedented public cancer data resource (Tomczak et al., 2015).

TCGA initiatives and others have demonstrated an extensive inter- and intratumor heterogeneity across breast, ovarian, and endometrial cancers (Salvesen et al., 2009; Kandoth et al., 2013; Ciriello et al., 2015; Grimaldi et al., 2020).

On the other hand, interesting results have been shown by the TCGA consortium (Pan-Cancer cohort) on hidden molecular feature similarities and differences across various cancers (Neapolitan et al., 2015; Hoadley et al., 2018; Liu et al., 2018; Sanchez-Vega et al., 2018).

Indeed, in 2018, the TCGA Research Network obtained a global view of the 33 human cancers and their genomic aberrations at multiple layers of regulations (Hoadley et al., 2018; Sanchez-Vega et al., 2018). Surprisingly, they found that breast cancer basal-like emerged as a distinct entity differently from the other identified tumor subtypes.

In the same year, Berger et al. performed an outstanding molecular study (Berger et al., 2018). They analyzed various genomic data from five female cancers, i.e., high-grade serous ovarian cystadenocarcinoma (OV), uterine corpus endometrial carcinoma (UCEC), cervical squamous cell carcinoma and endocervical adenocarcinoma (CESC), uterine carcinosarcoma (UCS), and invasive breast carcinoma (BRCA). Berger et al. highlighted unique molecular features (somatic aberrations and mutated genes), clinically relevant subtypes, and potentially novel therapeutic targets among five hormone-dependent cancers (Berger et al., 2018). From a molecular point of view, several multi-omics studies of each single tumor suggested interesting uterus endometrial genetic classifications (Kandoth et al., 2013). Also, other studies dissected multiscale genetic aberrations by looking at clinical outcomes and/or histopathological classifications (Sørlie et al., 2001; Mischel et al., 2003; Tothill et al., 2008; Dai et al., 2015).

However, although these cancers are clinically managed as distinct entities, the literature findings of co-occurring driver genes among these three cancers, such as TP53, PI3K pathway members, and GATA3 genes (Kandoth et al., 2013; Neapolitan et al., 2015; Hoadley et al., 2018; Liu et al., 2018; Sanchez-Vega et al., 2018), led us to combine these three female-specific and hormone-sensitive cancers. We merged all phenotypes to evaluate intertumor similarities and cross talks between transcriptomic and protein–protein interaction networks.

There is an increasing interest in studying similarities across cancer types. Several studies combined the results of multiple tumor types by meta-analysis methods (Pihur and Datta, 2008). Other recent approaches merged data together from multiple cancer types to create a whole integrated dataset for analyses (Berger et al., 2018; Hoadley et al., 2018).

Moreover, among computational approaches, network-based methods are gaining great attention in precision medicine (Oulas et al., 2019). A widely used network-based method is the weighted co-expression network WGCNA algorithm (Langfelder and Horvath, 2008). For instance, this approach was used by Hamed et al., to explore breast cancer tumor biology (Hamed et al., 2015). In addition, Zhang et al., carried out an integrative approach for breast and ovarian cancers (Zhang, 2018). They constructed a co-expression network G1 (BRCA) and G2 (OV) consisting of 6,779 genes and defined the numbers of clusters M in both networks by a spectral clustering approach (Zhang, 2018). On the other hand, knowledge-based integrative approaches have also been applied to women cancers. Indeed, Bhyan et al., achieved 52 common driver genes in breast, endometrial, ovarian, and cervical cancers using specialized disease database annotations (Bhyan et al., 2019).

In this study, we developed a bioinformatics workflow to capture expression similarity signatures across three female-specific cancer types regardless of their tissue or organ origin and exploit putative regulatory protein–protein interaction (PPI) networks underlying common tumorigenic processes.

We integrated transcriptomic profiling of publicly available RNA sequencing (RNA-Seq) data related to 148 normal and 2,015 female tumor samples from the TCGA breast (BRCA), uterine corpus endometrial carcinoma (UCEC), and ovarian (OV) projects.

We carried out a principal component analysis (PCA)-based unsupervised feature extraction (FE) approach followed by enrichment analyses and co-regulatory PPI networks to jointly capture expression similarity signatures underlying the three female-specific hormone-dependent cancers. Thus, we leveraged the bioinformatics software Ingenuity Pathway Analysis (IPA) to assess the pathways regulated by these genes and investigate the putative PPI networks promoting cancer progression.

## Materials and Methods

### TCGA Data Download and Preprocessing

TCGA-BRCA, TCGA-OV, and TCGA-UCEC primary solid tumors and solid tissue normal HT-Seq raw counts were downloaded using TCGAbiolinks version 2.14.1 (Colaprico et al., 2016). In the TCGA-BRCA dataset, we filtered out 12 male samples. Overall, we analyzed transcriptomic data of 2,163 female samples including primary tumors (mixed histology) and solid tissue normal for TCGA-BRCA (113 cases) and TCGA-UCEC (35 cases) (ovarian RNA-Seq data, not available for solid tissue normal samples). Mixed histology annotations were retrieved from the clinical data, downloaded from the GDC portal (accessed on August 4th, 2020). We provided the TCGA patient ID, project ID, and sample type metadata (see Supplementary Table 1).

### Integrated TCGA Data Analysis

TCGA Batch Effects Viewer tool (https://bioinformatics.mdanderson.org/BatchEffectsViewer/) was used to evaluate the existence of batch effect(s) in each RNA-Seq dataset. From a total of 60,483 genes, we only retained those genes expressed at a counts per million (CPM) above 0.5 in at least 90% of the samples (*n* = 13,720), and we only considered filtered genes, which were annotated in Ensembl version 86 based on the GRCh38 genome build (*n* = 13,703). Then, we performed a global data normalization via the upper quartile approach (UQUA) and removed the gene length bias. Finally, we estimated gene expression as log2(normalized count + 1) and only considered genes with |logFC|>1 between normal and tumor conditions.

### PCA-Based Unsupervised Feature Extraction

To extract the most informative tumor-associated genes and remove any redundancy in the transcriptomic dataset, we performed a PCA-based unsupervised FE approach, which is based on correlation analysis. The FE approach was applied to a unique dataset, composed of the three tumors. We retained a sufficient number of principal components (PCs), which were able to explain at least 60% of the total variance. We also evaluated the most significantly associated variables (genes) with each one of these components. These variables, called eigenvalues, were considered significantly associated with PCs if two requirements were fulfilled: (i) Pearson's correlation value was higher than the 95th percentile and (ii) Pearson's correlation *p* < 0.001.

### Integrated Pathways and PPI Network Analyses

We carried out integrated functional analyses using the Ingenuity Pathways Analysis software (IPA, QIAGEN, Inc. Redwood City, CA, USA) (Krämer et al., 2014). We investigated the molecules' subcellular locations and type(s) using the manually curated annotations included in the IPA Knowledge Base (IPA KB). IPA researchers categorized gene annotations based on all types of biological functions useful for IPA software downstream analyses (Krämer et al., 2014).

We carried out functional enrichment analysis for the 1,643 tumor-associated genes. We used the Ingenuity Knowledge Base (release date: 2020-06-01) as the reference set for the integration of functional analyses and protein–protein interaction networks. One Ensembl ID (ENSG00000279010, unmapped sense-overlapping feature) was excluded after IPA mapping IDs. Overall, we found 158 enriched pathways with the statistical score threshold [–log(*p*-value) based on Fisher's exact test] ≥1.3. We analyzed the top 10 enriched and statistically significant pathways within a score threshold ≥5. Protein–protein interaction networks were based on the 1,642 ID expression signatures. They were generated with *n* = 35 maximum number of molecules per network and node types which can be direct and indirect relationships excluding the impact of chemical drugs. Only experimentally validated observations were included in the IPA KB, release 2020. Networks were ranked based on the hypergeometric distribution, which was calculated by the right-tailed Fisher's exact test.

## Results

### Integrative Computational Approach

**Figure 1:**
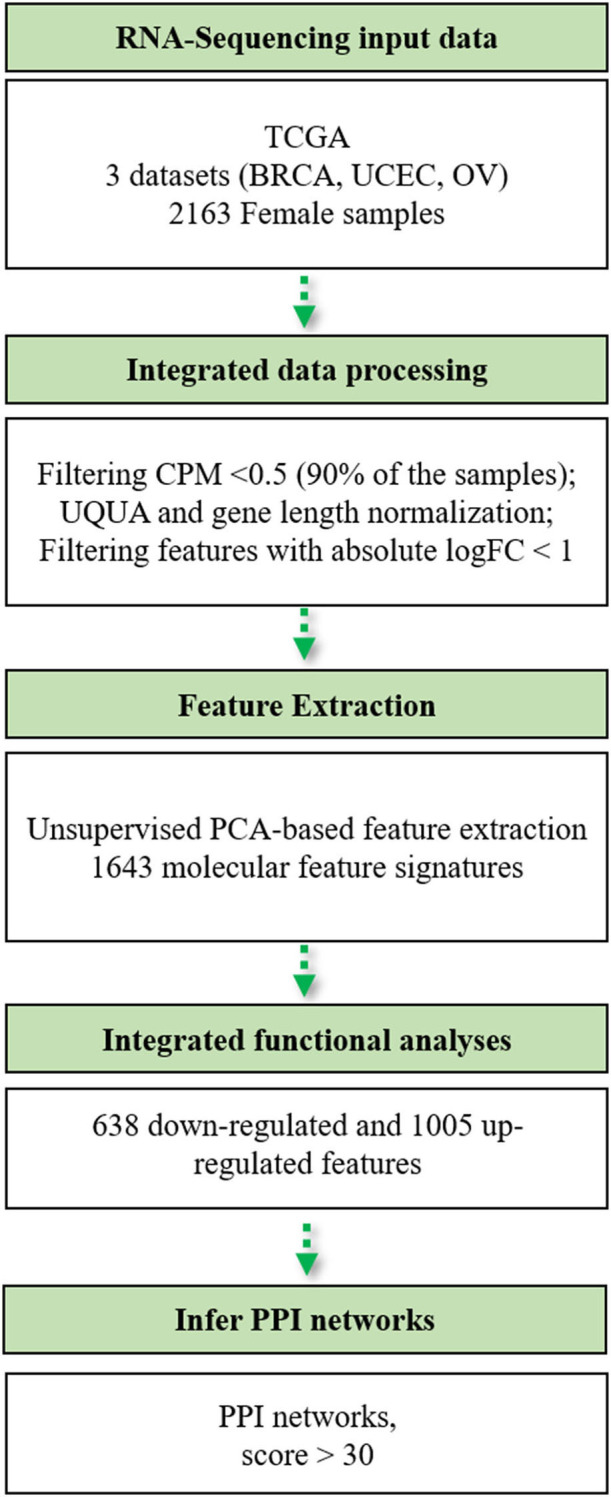
Flowchart of the bioinformatics pipeline used in this study. TCGA, The Cancer Genome Atlas; BRCA, breast cancer; UCEC, uterine cervix endometrial cancer; OV, ovarian cancer; CPM, counts per million; UQUA, upper quartile normalization method.

The bioinformatics workflow followed in this study is shown in Figure 1. We carried out an integrated analysis of gene expression profiling from three TCGA publicly available datasets (Table 1). Metadata is provided in Supplementary Table 1 (see Data Availability Statement). We integrated the transcriptomic (RNA-Seq) profiles from 2,015 primary tumors and 148 solid tissue normal samples from TCGA-BRCA, TCGA-UCEC, and TCGA-OV datasets to capture biological relationships among these three female tumor types. From a total of 60,483 genes, we only retained those genes expressed at a CPM above 0.5 in at least 90% of the samples (*n* = 13,720) and annotated in the Ensembl database (version 86) based on the GRCh38 genome build (*n* = 13,703). We normalized all filtered counts using the upper quartile method, considering gene length. Finally, we included genes with the absolute logFC>1 between normal and tumor conditions, thus obtaining a total of 2,460 genes to be used for downstream analysis.

**Table 1:** Summary of TCGA dataset handling used in this study.

**TCGA dataset**	**RNA-Seq data (BRCA/OV/UCEC)**
BRCA/OV/UCEC Tumors (cases)	1,102^a^/374/551
BRCA/OV/UCEC Normal (cases)	113/NA^b^/35
Total samples	2,175

a *12 male samples filtered out from the TCGA–BRCA dataset to analyze only female samples*.

b *Not available*.

### Unsupervised PCA-Based Feature Extraction

In order to extract the most informative tumor-associated genes, we performed a PCA based on the unsupervised FE approach. We only retained the PCs that were able to explain at least 60% of data variance. Therefore, the first 20 PCs were extracted (Supplementary Figure 1). Then, we evaluated the most statistically significant correlated variables (Pearson's correlation value higher than 95th percentile and Pearson's correlation with *p* < 0.001) with each one of the components, by extracting the associated eigenvalues. By applying this approach, we reduced the dimensionality of our transcriptomic dataset to 1,643 most informative molecular signatures that characterized the expression similarity signatures of intertumor phenotypes. By performing the PCA on these 1,643 genes, tumor samples were randomly distributed in the two-dimensional space (Supplementary Figure 2). Out of the 1,643 expression signatures, 1,561 were protein-coding genes.

### Integrative Functional Analyses

Transcriptomic analyses resulted in 1,643 expression signatures across the three female-specific and hormone-sensitive cancers. We analyzed the overall expression changes of the 1,643 tumor-associated genes, by using the Ingenuity Pathway Analysis QIAGEN software.

IPA mapped 1,642 out of the 1,643 expression signatures (ENSG00000279010, sense overlapping unmapped), of which 637 were downregulated and 1,005 were upregulated genes in the three tumors with respect to the normal samples.

**Figure 2:**
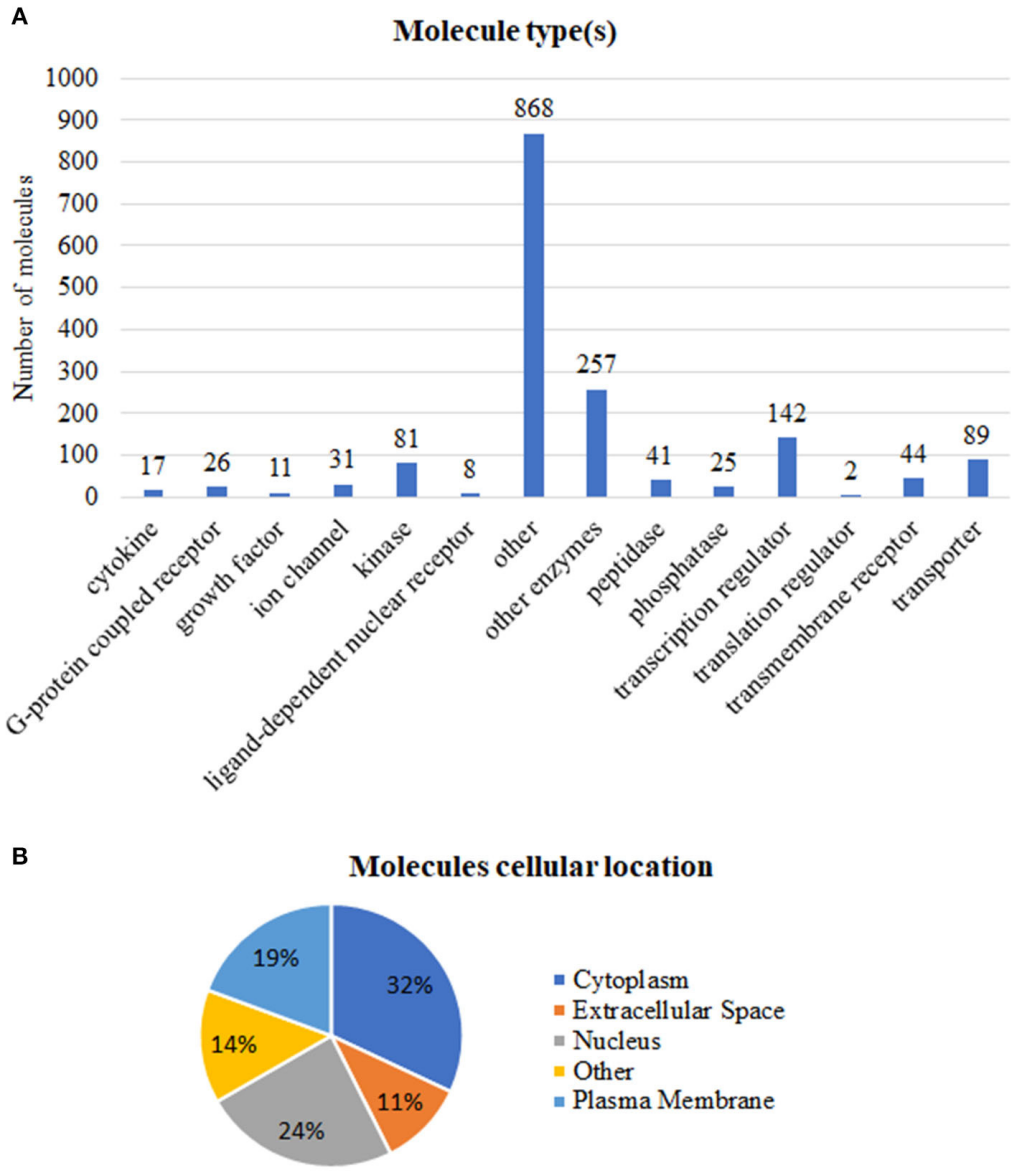
Functional annotations of the 1,642 tumor-associated genes. **(A)** Number of molecules per type. **(B)** Percentage of molecules in accordance to subcellular location. Annotations based on the Ingenuity Pathway Analysis manually curated Knowledge Base, QIAGEN software, 2020.

We grouped these expression signatures according to IPA software Knowledge Base (KB) biological molecular type(s) (Figure 2A) and subcellular location (Figure 2B) annotations. In our dataset, most protein-coding genes were enzymes, transcription factors, and transporter (Figure 2), while the “other” category encompassed not only protein-coding genes but also proteins whose biological function is still uncharacterized and/or cytoskeletal proteins such as members of the actin family (Pollard, 2016; Svitkina, 2018).

**Figure 3:**
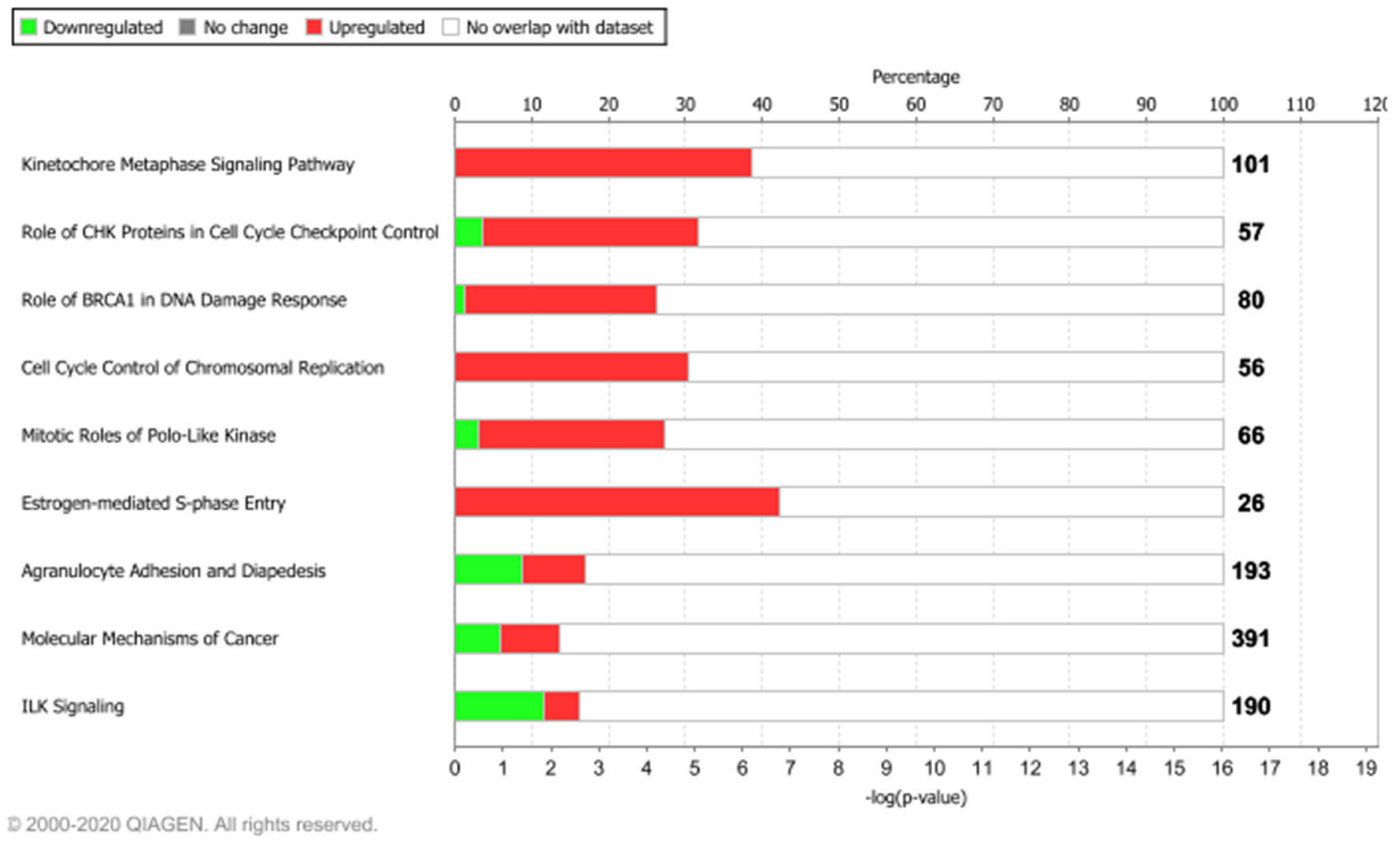
Functional enrichment analysis of the 1,642 tumor-associated genes. The top 10 enriched pathways, ranked for enrichment score with threshold ≥5 [–log(*p*-value), based on Fisher's exact test]. Bolded numbers on the right represent the total amount of genes within each IPA canonical pathway. Dataset analyzed by the Ingenuity Pathway Analysis, QIAGEN software, 2020.

To understand whether the tumor-associated genes (intertumor similarity signatures) orchestrated oncogenic signaling pathways, we carried out functional enrichment analyses. We displayed the most enriched pathways in Figure 3, whereas the overall results are shown in Supplementary Table 2.

Among the top 10 enriched pathways, ranked for enrichment score, we identified pivotal signaling pathways linked to many important cellular processes such as mitotic division, cell cycle checkpoint control, and S-phase entry regulated by estrogens.

We found that about 39% (39/101) molecular common signatures, enriched the “Kinetocore Metaphase Signaling Pathway” with the highest enrichment score (Supplementary Table 2). The “Kinetocore Metaphase Signaling Pathway” allows sister chromatid segregation during mitosis (Tanaka, 2013). Furthermore, during this process, some hub genes took part in essential protein complexes (i.e., KNL1/MIS12/NDC80 protein complexes) with a central role in connecting kinetochores to microtubules (Tanaka, 2013). These genes are also regulated by several enzymes including AURKB, a component of the chromosomal passenger complex (CPC) that plays a major role in the correction of erroneous attachments through phosphorylation of the KMN network and the SKA complex (Tanaka, 2013). Those 39 molecules were upregulated in our dataset (pink genes, Supplementary Figure 3), in agreement with their biological role. Moreover, IPA regulatory predictions revealed that the expression changes associated with the 1,642 intertumor similarity signatures may lead to the activation of cell proliferative mechanisms (orange bar) and inhibition of cell cycle checkpoint control (blue bar) (Supplementary Figure 4).

Other key biological processes promoting tumor growth were enriched by upregulated genes (Figure 3, Supplementary Figure 4). In particular, we found that about 30% (17/56) molecular signatures enriched the “Cell Cycle Control of Chromosomal Replication” biological process (CDC45, CDC6, CDC7, CDK1, CDK16, CDK18, CDT1, DBF4, DNA2, LIG1, MCM2, MCM4, ORC1, ORC6, PCNA, POLD1, TOP2A), while about 42% (11/26) enriched the “Estrogen-Mediated S-Phase Entry” signaling pathway (Figure 3, Supplementary Figure 4) composed of CCNA2, CCNE1, CCNE2, CDC25A, CDK1, E2F1, E2F2, E2F3, E2F5, E2F7, and E2F8 protein-coding genes.

Thus, our *ad-hoc* bioinformatics workflow, designed to capture transcriptional signatures of interfemale tumors, confirmed that most of the identified similarities could impact on pro-oncogenic signaling pathways and hormone-stimuli-dependent cell proliferation.

### Protein–Protein Interaction Networks

In summary, functional analyses highlighted that the 1,642 molecular signatures, jointly associated with the three female-specific cancan, and may regulate important tumorigenic pathways including estrogen-dependent S-phase entry.

Thus, we leveraged the IPA network analytic algorithms based on mammalian genes and their products driving direct or indirect relationships at multiple levels to infer molecular mechanisms associated with the expression changes of the 1,642 tumor-associated genes.

**Figure 4:**
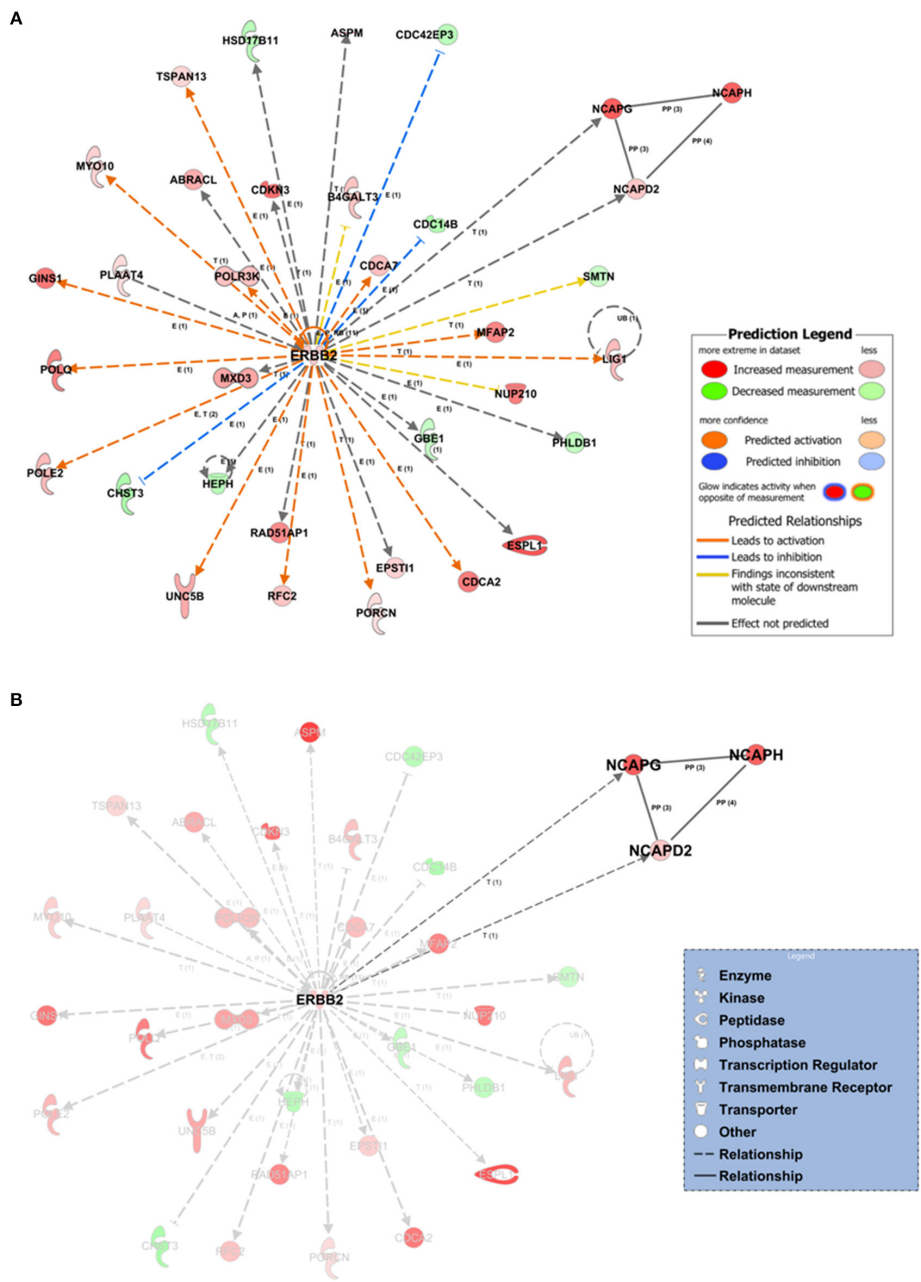
Network inference underlying the 1,642 tumor-associated genes. **(A)** The top-scoring network (the first over 25 networks) based on the statistical approach and manually curated IPA Knowledge Base (IPA KB). Network edges associated with ERBB2 direct (continuous lines) or indirect relationships (dashed lines) at expression, transcription, or protein–protein-binding level. *In silico* prediction of the ERBB2 downstream impact within the network is highlighted by orange-pointed arrowheads (activation effect) or blue blunt arrowheads (inhibitory effect) based on the expression changes and the experimentally observed evidences within the IPA KB. **(B)** Protein–protein interaction subnetwork within network 1 formed by the non-SMC condensin I complexes (NCAPD2, NCAPG, NCAPH). E, expression; T, transcription; P, phosphorylation/dephosphorylation; PP, protein–protein interaction (binding).

Within the PPI networks, we found that ZBTB17, ERBB2, TGFB1, CSF2, and FOXM1 proteins could potentially act as upstream regulators, due to their expression trend and target molecules presented within the dataset. We collected a total of 25 statistically significant networks associated with our dataset (Supplementary Table 3). Among them, the top-scoring network (Figure 4) was centered on *ERBB2* gene coding for the erb-b2 receptor tyrosine kinase 2. The annotation of molecules involved in network 1 (Figure 4) is reported in Supplementary Table 4. The prioritized ERBB2 network had multiple indirect relationships including (i) the DNA ligase 1 (LIG1), POLE2, and RFC2 proteins, involved in the nucleotide excision repairs; (ii) the extra spindle pole bodies like 1 separase (ESPL1) and myosin X (MXD3), members of the “Kinetocore Metaphase Signaling Pathways”; and (iii) the transmembrane receptor unc-5 netrin receptor (UNC5B) involved in the “Axonal Guidance Signaling” signaling pathway (Figure 4, Supplementary Figure 4). We also highlighted a subnetwork of direct protein–protein interactions formed by the non-SMC condensin I complexes (NCAPD2, NCAPG, NCAPH) (Figure 4B). Those complexes are associated with the DNA condensation during cell division and have transcriptional-based relationships with ERBB2 signaling pathway and cancer development (Hua et al., 2018).

The IPA Knowledge Base and the 1,642 expression gene trends enabled us to generate a hypothesis on the potential upstream and downstream effects of activation or inhibition of molecules within the networks. IPA suggested that in network 1, ERBB2 could indirectly inhibit three neighbors (blue blunt arrowheads, Figure 4A) and activate several network neighbors (orange point arrowheads, Figure 4A). For this application, activity is assumed to be at the protein level for the nodes of a network or pathway. Thus, we found potential ERBB2-mediated inhibition of the cell division cycle 14B (CDC14B) phosphatase, involved in the PI3K signaling pathway; the CDC42 effector protein 3 (CDC42EP3), a cytoskeleton regulatory protein binding involved in Rho GTPase signaling; and the carbohydrate sulfotransferase 3 (CHST3) enzyme, a member of the carbohydrate sulfotransferase 3 family. Moreover, ERBB2, over its positive feedback (circular arrow, Figure 4A), could have expression-mediated relationships with ABRACL, B4GALT3, CDC14B, CDC42EP3, CDC42EP3, CDCA2, CDCA7, CHST3, EPSTI1, GBE1, GINS1, HEPH, HSD17B11, LIG1, NUP210, PHLDB1, POLE2, POLQ, POLR3K, RFC2, TSPAN13, and UNC5B and transcriptional-mediated relationships with CDKN3, ESPL1, MFAP2, MXD3, MYO10, NCAPD2, NCAPG, POLE2, PORCN, RAD51AP1, and SMTN, leading to their upregulation.

As previously described, the ERBB2-centered network (network 1) resulted as top scoring out of 25 possible networks (Supplementary Table 3). Out of network 1, four other networks are also interesting because they were endowed with a relatively high score (>30) (Supplementary Table 3, Supplementary Figures 5, 6).

## Discussion

Breast, ovarian, and endometrial cancers are hormone-sensitive cancers and showed an extensive inter- and intratumor heterogeneity (Salvesen et al., 2009; Kandoth et al., 2013; Ciriello et al., 2015; Grimaldi et al., 2020).

Integrative analyses of these female-specific and hormone-sensitive cancers may decipher still cryptic molecular features. Several integrative analyses showed the co-occurrence of driver genes across distinct tumor entities and the existence of common molecular mechanisms for their progression also between breast and gynecological malignancy entities (Neapolitan et al., 2015; Berger et al., 2018; Hoadley et al., 2018; Liu et al., 2018; Sanchez-Vega et al., 2018; Bhyan et al., 2019; Zhong et al., 2020).

In this study, we carried out a computational analysis by integrating three hormone-sensitive female cancer datasets, i.e., breast, ovarian, and endometrial TCGA cohorts. Our goal was to identify intertumor expression similarities and infer putative co-regulatory protein–protein interaction networks with possible implications on estrogen-dependent mechanisms. To address this aim, we developed an *ad-hoc* bioinformatics pipeline only on gene expression profiling, including a PCA-based unsupervised FE method, combined with global normalization in order to capture global intertumor heterogeneity.

We integrated publicly available transcriptomic data (RNA-Seq) of 2,163 female samples from breast, endometrial, and ovarian TCGA datasets. Worth noting, we grouped tumor and normal samples to perform an overall upper quartile normalization step of gene expression data. Thus, we minimized the baseline expression of tissue-related genes in order to capture female cancer similarity in terms of expression signatures. We then carried out a PCA-based unsupervised FE approach resulting in 1,643 expression similarity signatures as the most informative tumor-associated genes. To assess whether these tumor-associated genes regulated oncogenic signaling pathways, we leveraged the commercial IPA software to analyze functional and downstream effects, based on PPI networks. Functional enrichment analyses resulted in a total of 158 statistically significant enriched pathways (*p* < 0.05), regulated by 1,642 breast, ovarian, and endometrial expression signature similarities. Above all pathways, we found top 10 biological processes such as the “Kinetochore Metaphase Signaling Pathway,” the “Cell Cycle Control of Chromosomal Replication,” and the “Estrogen-Mediated S-Phase Entry” signaling pathway. The latter is composed of CCNA2, CCNE1, CCNE2, CDC25A, CDK1 protein-coding genes (CCNA2, CCNE1, CCNE2, CDC25A, CDK1) and the E2F transcription factor family members.

The activity of CDKs, along with the retinoblastoma tumor-suppressor RB protein and E2F pathway, is a crucial regulator of cell cycle progression (G1–S transition). Indeed, the deregulation of the CDK–RB–E2F pathway is recurrent in almost all human malignancies (Kent and Leone, 2019).

Oncogenic signaling pathways found in our study are in agreement with previous literature findings (Zhang, 2018; Zhong et al., 2020). To the best of our knowledge, we carried out for the first time a transcriptomic integrated PCA-based feature extraction approach to evaluate intertumor expression signatures and putative protein–protein interaction networks.

For instance, Bhyan et al. identified several common genes across four cancers in women involved in endogenous hormonal regulation pathways (Bhyan et al., 2019). Neapolitan et al., discovered in Pan-Cancer analysis which signal transduction pathway (STPs) were implicated in cancer or cancer subtypes focusing the attention on “notable” altered pathways (Neapolitan et al., 2015). These biological processes were the focal adhesion pathway, P13k–Akt pathway, Rap1 pathway, and calcium signaling pathway. Several bioinformatics analyses also revealed that ovarian and uterine cancer shared oncogenic pathways (Neapolitan et al., 2015; Zhang, 2018) as well as breast and ovarian cancer cells may even have co-occurrence of cancer stem cells (CSCs) as a hormone-stimuli response (Wang et al., 2013). In addition, we investigated putative co-regulatory PPI networks underlying the 1,642 expression similarity signatures. Interestingly, we identified a key network centered on ERBB2 (Erb-B2 Receptor Tyrosine Kinase 2), commonly known as HER2, widely recognized as a breast cancer biomarker and a relevant player in gynecologic malignancies (Erickson et al., 2020). There are several findings on ERBB2 gene amplification, also commonly known as member of the epidermal growth factor receptor family, in multiple tumor types including early endometroid uterine cancer (Kandoth et al., 2013; Abdel Azim et al., 2017) and ovarian cancer (Luo et al., 2018). Many efforts have been devoted in the last years to target HER2 tyrosine kinase by various chemical and biological drugs for several cancers (Yan et al., 2014). In the ERBB2 network, we also found a subnetwork of direct protein–protein interactions formed by the non-SMC condensin I complexes (NCAPD2, NCAPG, NCAPH), a vital complex associated with the ERBB2 signaling pathway and cancer development (Hua et al., 2018). Currently, understanding the role of HER2 in gynecological cancers is an interesting field of investigation. The prognostic value of ERBB2 in ovarian cancer has been recently evaluated in 5,180 ovarian cancer patients and was negatively correlated with overall survival outcome (Luo et al., 2018). Importantly, estrogens act not only through genomic (nuclear ER receptors) but also through non-genomic actions via ligand binding (Arnal et al., 2017).

The genomic action, also known as classical estrogen action, consists of estrogen receptor α (ERα) cytosolic activation by E2, nuclear ERα dimerization, and direct or indirect DNA binding in order to regulate target genes involved in cell cycle, S-phase entry, cell migration, proliferation, and differentiation (Hewitt et al., 2016; Hua et al., 2018). Estrogens, such as 11β-estradiol (E2), bind to ERα expressed on breast, ovarian, and endometrial cancer cells and regulate the expression of target genes. So far, in mammals, two membrane receptors, ERα and ERβ, expressed on multiple organ sites, have been mapped. The majority of breast cancers (about 70%) express ERα, which is the therapeutic target for hormone-based therapy. Breast, ovarian, and endometrial cancers share both receptors; however, ERα and ERβ signaling transductions have different biological effects. ERα knockout mice model experiments have shown a strong impact on sexual maturation leading to infertility (Hewitt et al., 2016). In contrast, mice models lacking Erβ showed reduced fertility and ovarian maturation efficiency (Hewitt et al., 2016). In addition, classical/genomic action has been more investigated than the estrogens' non-genomic action; as such, downstream biological effects are less known. In recent years, *in vivo* studies on mice models are helping to understand the mechanistic links of estrogens through non-genomic action (Adlanmerini et al., 2014; Hua et al., 2018).

Non-genomic action of estrogens may act *via* the PI3K/AKT pathway that represents one of the molecular mechanisms to mediate the ERBB2/ERBB3 oncogenic signaling pathway (Arnal et al., 2017).

Probably, the top-scoring network predicted in this study might uncover the non-genomic estrogen-dependent downstream effect orchestrated by ERBB2 in these female-specific cancers. Certainly, the herein *in silico* generated hypotheses need precautions and deserve further investigations.

Nevertheless, this study has some limitations. We conducted the analyses on TCGA publicly available data without an internal validation dataset. Indeed, the TCGA provided us the chance to carry out an intertumor study using next-generation sequencing (NGS) conspicuous data of tumor and normal samples. We are also aware that our results cannot be generalized since we did not have any experimental validation. However, our *in silico* predictions and the protein–protein interaction networks may open the way to further investigations. Our methodological approach proved to be efficient in selecting expression similarity signatures leading to tumorigenic processes and, thus, may be a useful workflow for studying other hormone-sensitive cancers such as thyroid and cervical cancers. Indeed, our integrated computational pipeline enabled us to capture the global intertumor (breast, endometrial, and ovarian) expression similarities regardless of their tissue of origin, with putative co-regulatory PPI networks orchestrated by ERBB2 underlying these female tumors.

## Data Availability Statement

The original contributions presented in the study are included in the article/Supplementary Materials, further inquiries can be directed to the corresponding author/s.

## Author Contributions

KP and MF: study design. KP and OA: methodology, data analysis, software, formal analysis, and writing—original draft preparation. MF and MI: validation. KP, OA, MZ, and RC: data download and curation. KP, OA, MZ, RC, MI, MS, and MF: writing—review and editing. MF and MS: supervision. MS: funding acquisition. All authors contributed to the article and approved the submitted version.

## Conflict of Interest

The authors declare that the research was conducted in the absence of any commercial or financial relationships that could be construed as a potential conflict of interest.
