# Supplementary material for: An Integrative Computational Approach Based on Expression Similarity Signatures to Identify Protein–Protein Interaction Networks in Female-Specific Cancers
Source: Front Genet. 2020 Dec 3;11:612521. doi: 10.3389/fgene.2020.612521 (PMC7793872; doi:10.3389/fgene.2020.612521)
Supplement: Supplementary file 1 [file Data_Sheet_1.PDF]

frontiers

# *Supplementary Material*

## 1 Supplementary Figures and Tables

### 1.1 Supplementary Figures

**Supplementary figure 1.**

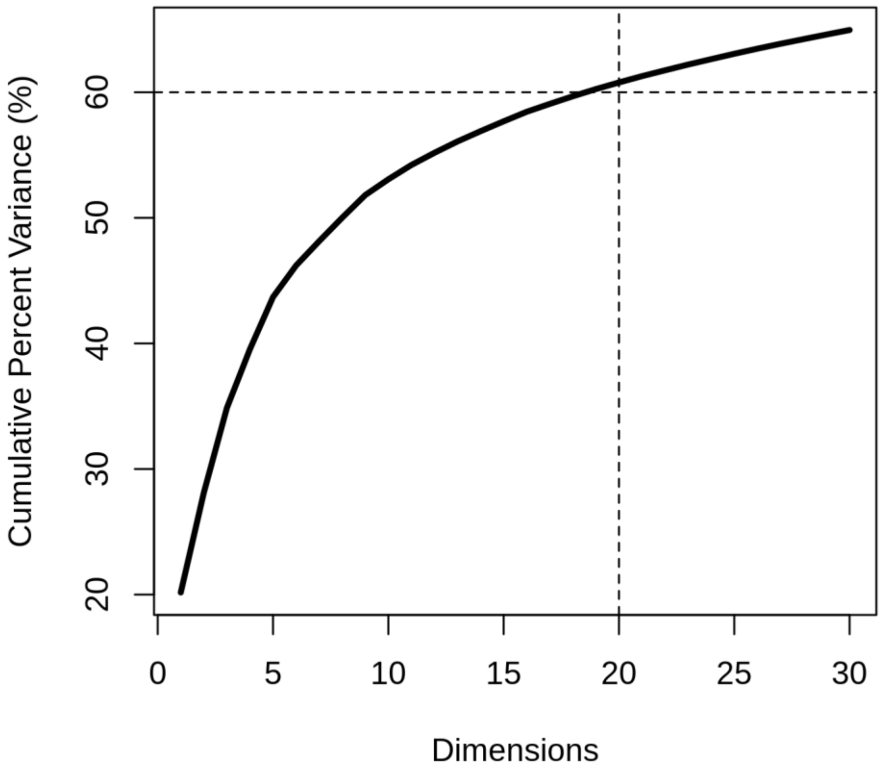


**Supplementary figure 1. Cumulative percent of variance.** The horizontal dashed line identifies the 60% of variance and the vertical dashed line identify the 20 PCs able to explain the 60% of the total variance.

**Supplementary figure 2.**

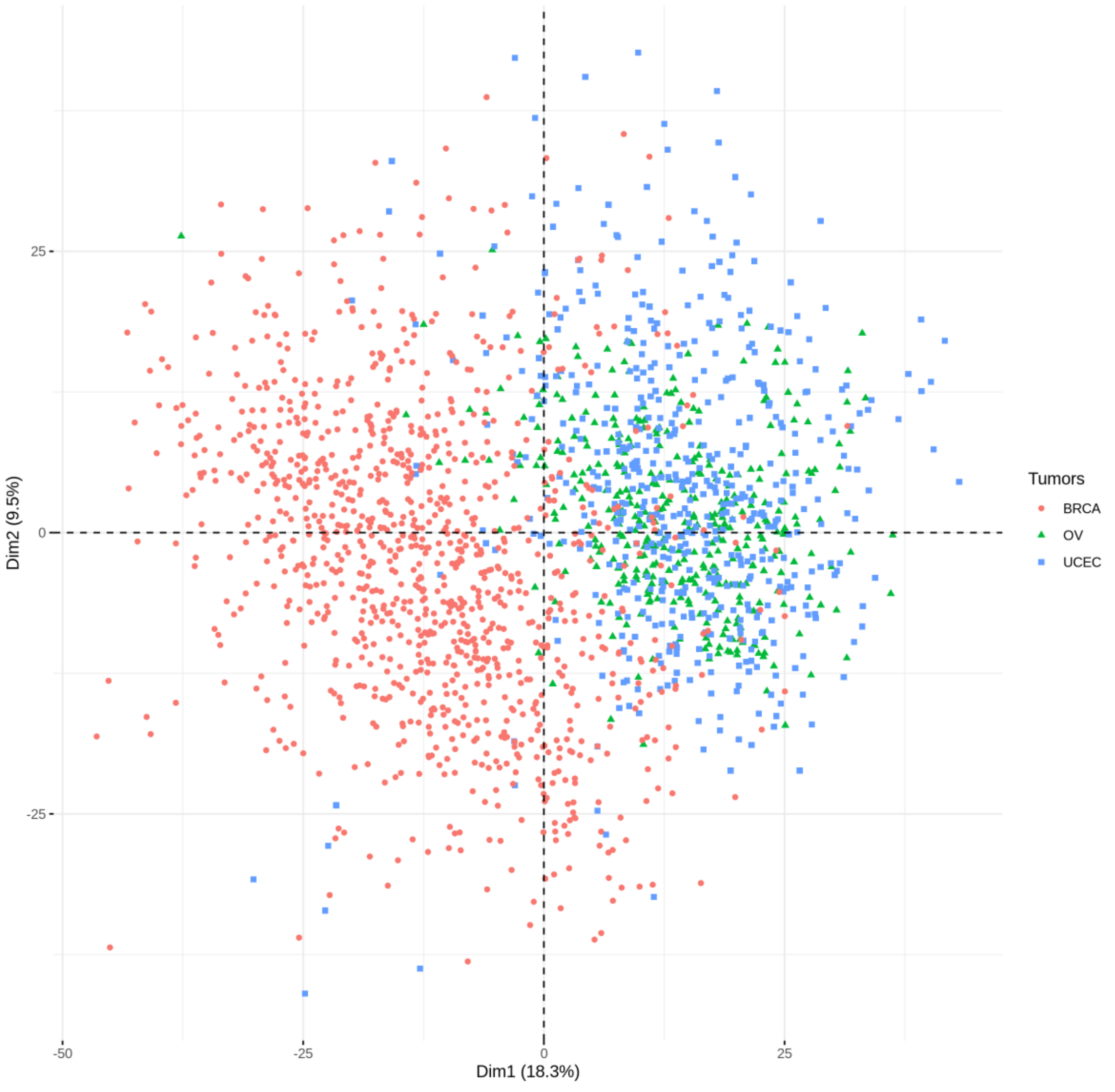


**Supplementary figure 2. PCA–based feature extraction analysis.** Samples are plotted by using the first two principal components. Samples from each tumor are coded by a different color.

**Supplementary figure 3.**

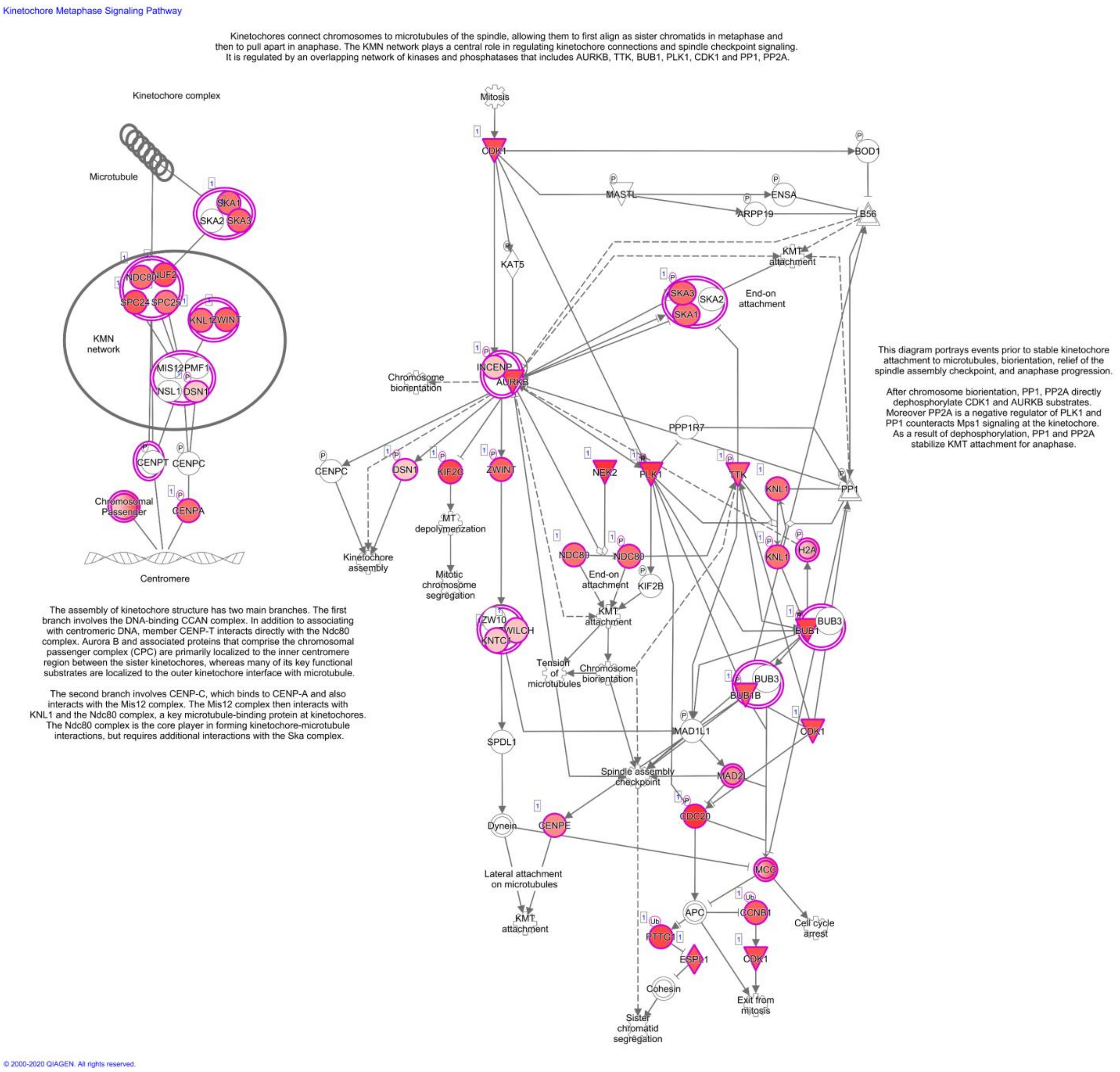


**Supplementary figure 3. Kinetocore Metaphase Signaling Pathway is the the top enriched pathway** (Fisher’s Exact right-tailed test, p-value << 0.001). All genes overlapping the signaling pathways (highlighted in pink) resulted up-regulated in BRCA,UCEC and OV primary tumor tissue compared with normal samples.

**Supplementary figure 4.**

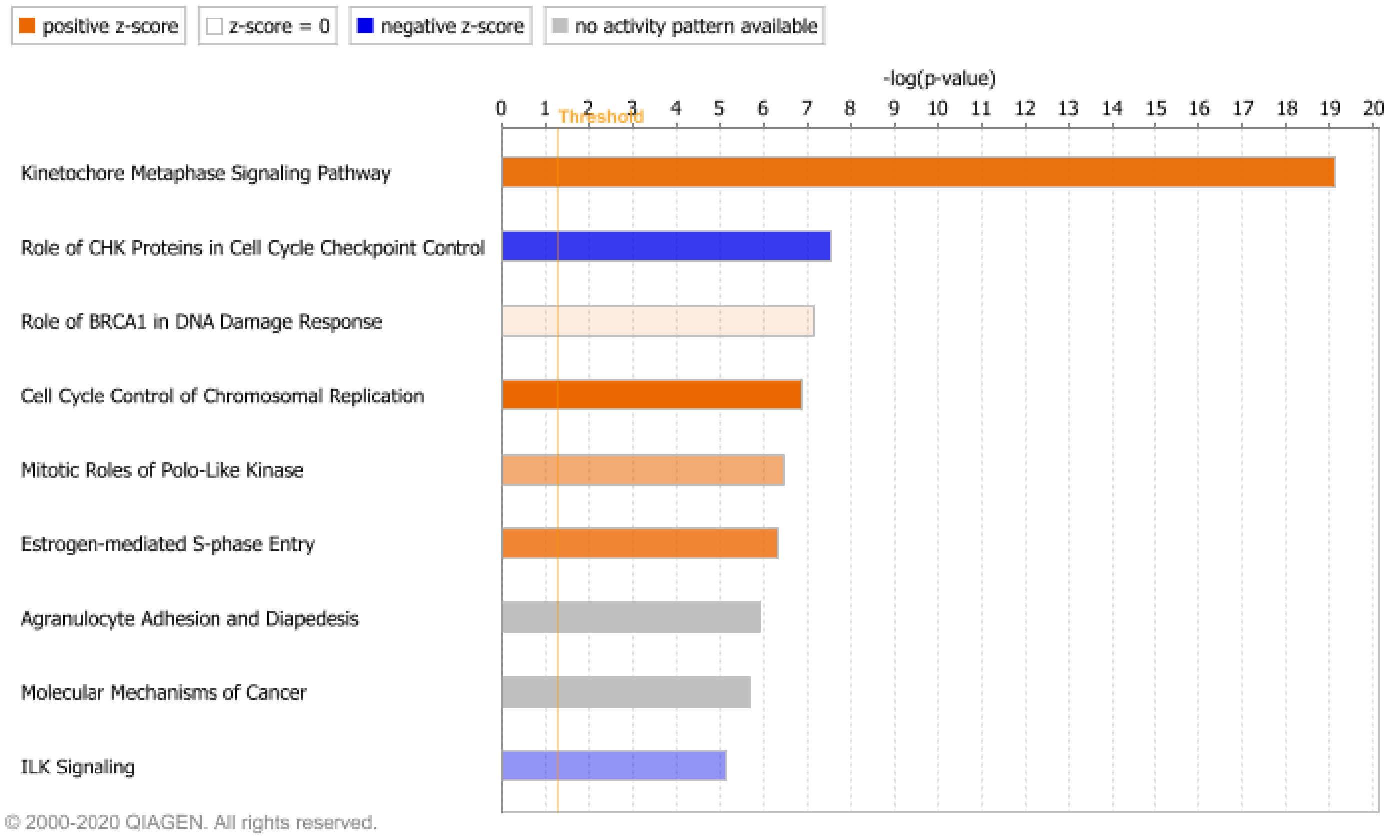


**Supplementary figure 4. Prediction of activated and inhibited signaling pathways.** The expression changes of genes involved in top 10 enriched pathway (Fisher's Exact right-tailed test, p-value << 0.001) are consistent with the activation (orange pathways) and inhibition (blue pathways) of the signaling pathways shown, in accordance with activation Z-score, Ingenuity Pathway Analysis QIAGEN software, 2020.

**Supplementary figure 5.**

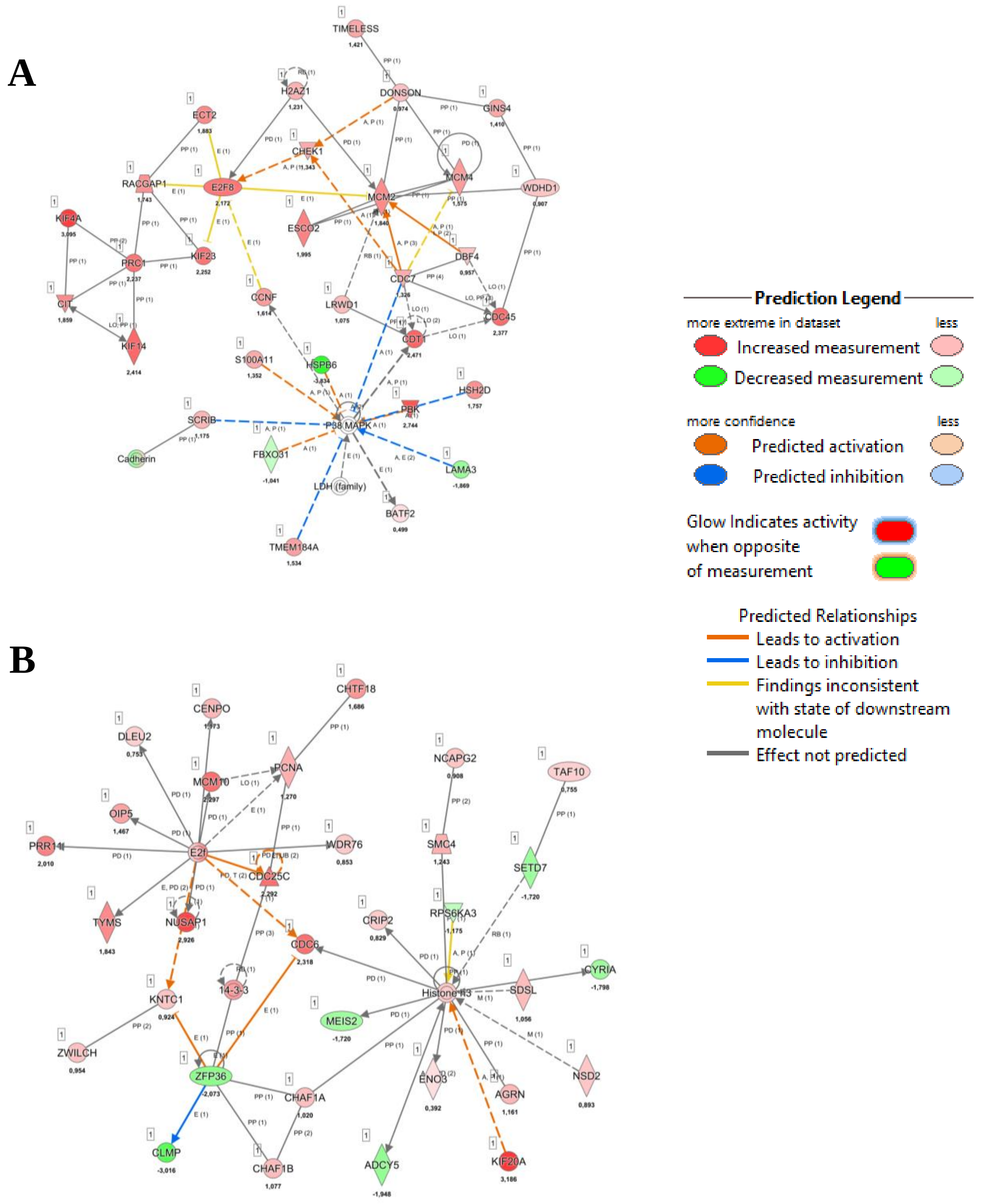


**Supplementary figure 5. Other top-scoring networks associated with the 1642 tumor associated genes**. A) The top-scoring network 2 (2nd over 25th networks). B) The top-scoring network 3 (3rd over 25th networks). Networks based on statistical approach and manually curated IPA Knowledge Base (IPA KB). Network edges with direct (continues lines) or indirect relationships (dashed lines) at expression, transcription or protein–protein binding level. *In silico* prediction of the downstream effect within the network are highlighted by orange pointed arrowheads (activation effect) or blue blunt arrowheads (inhibitory effect) based on the expression changes (up- and down-regulated genes in red and green, respectively) and the experimentally observed evidences within the IPA KB. Abbreviations: E= Expression; T= Transcription; P= Phosphorylation/Dephosphorylation; PP= Protein-Protein interaction (binding). Ingenuity Pathway Analysis QIAGEN software, 2020. Ingenuity Pathway Analysis QIAGEN software, 2020.

**Supplementary figure 6.**

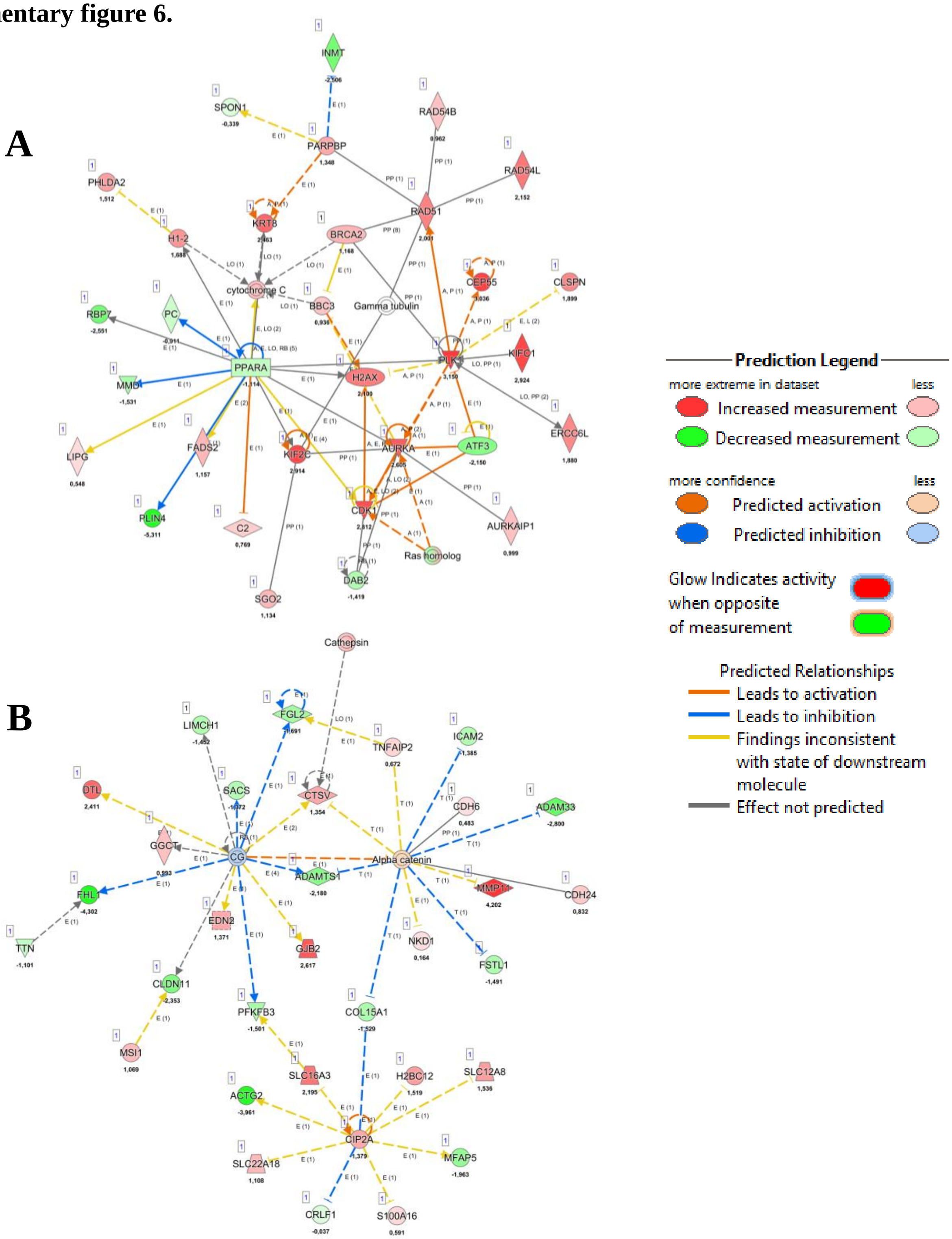


**Supplementary figure 6. Other top-scoring networks associated with the 1642 tumor associated genes**. A) The top-scoring network 4 (4th over 25th networks). B) The top-scoring network 5 (5th over 25th networks). Networks based on statistical approach and manually curated IPA Knowledge Base (IPA KB). Network edges with direct (continues lines) or indirect relationships (dashed lines) at expression, transcription or protein–protein binding level. *In silico* prediction of the downstream effect within the network are highlighted by orange pointed arrowheads (activation effect) or blue blunt arrowheads (inhibitory effect) based on the expression changes (up- and down-regulated genes in red and green, respectively) and the experimentally observed evidences within the IPA KB. Abbreviations: E= Expression; T= Transcription; P= Phosphorylation/Dephosphorylation; PP= Protein-Protein interaction (binding). Ingenuity Pathway Analysis QIAGEN software, 2020.

## 1.2 Supplementary Tables

**Supplementary Table 1. TCGA datasets used in this study.**

| TCGA_patient_barcode | Project ID | Sample_type |
|---|---|---|
| TCGA-E9-A1NF | TCGA-BRCA | Primary Solid tumor |
| TCGA-D8-A27M | TCGA-BRCA | Primary Solid tumor |
| TCGA-BH-A0GZ | TCGA-BRCA | Primary Solid tumor |
| TCGA-BH-A18V | TCGA-BRCA | Primary Solid tumor |
| TCGA-A7-A13G | TCGA-BRCA | Primary Solid tumor |
| TCGA-C8-A275 | TCGA-BRCA | Primary Solid tumor |
| TCGA-AN-A0XS | TCGA-BRCA | Primary Solid tumor |
| TCGA-OL-A5RW | TCGA-BRCA | Primary Solid tumor |
| TCGA-OL-A5RX | TCGA-BRCA | Primary Solid tumor |
| TCGA-E9-A1RD | TCGA-BRCA | Primary Solid tumor |
| TCGA-EW-A6SD | TCGA-BRCA | Primary Solid tumor |
| TCGA-V7-A7HQ | TCGA-BRCA | Primary Solid tumor |
| TCGA-BH-A0GY | TCGA-BRCA | Primary Solid tumor |
| TCGA-A8-A08I | TCGA-BRCA | Primary Solid tumor |
| TCGA-OL-A66H | TCGA-BRCA | Primary Solid tumor |
| TCGA-A1-A0SB | TCGA-BRCA | Primary Solid tumor |
| TCGA-AC-A3QP | TCGA-BRCA | Primary Solid tumor |
| TCGA-A2-A4RW | TCGA-BRCA | Primary Solid tumor |
| TCGA-E2-A14V | TCGA-BRCA | Primary Solid tumor |
| TCGA-AO-A0J8 | TCGA-BRCA | Primary Solid tumor |
| TCGA-E2-A14Y | TCGA-BRCA | Primary Solid tumor |
| TCGA-AO-A0JE | TCGA-BRCA | Primary Solid tumor |
| TCGA-EW-A1P1 | TCGA-BRCA | Primary Solid tumor |
| TCGA-E2-A107 | TCGA-BRCA | Primary Solid tumor |
| TCGA-AC-A2QH | TCGA-BRCA | Primary Solid tumor |
| TCGA-B6-A0IB | TCGA-BRCA | Primary Solid tumor |
| TCGA-AN-A0FW | TCGA-BRCA | Primary Solid tumor |
| TCGA-B6-A0X1 | TCGA-BRCA | Primary Solid tumor |
| TCGA-BH-A0EA | TCGA-BRCA | Primary Solid tumor |
| TCGA-A2-A3XY | TCGA-BRCA | Primary Solid tumor |
| TCGA-B6-A0RL | TCGA-BRCA | Primary Solid tumor |
| TCGA-A7-A4SE | TCGA-BRCA | Primary Solid tumor |
| TCGA-BH-AB28 | TCGA-BRCA | Primary Solid tumor |
| TCGA-BH-A0DI | TCGA-BRCA | Primary Solid tumor |
| TCGA-GM-A2DB | TCGA-BRCA | Primary Solid tumor |
| TCGA-AO-A0J7 | TCGA-BRCA | Primary Solid tumor |
| TCGA-3C-AALJ | TCGA-BRCA | Primary Solid tumor |
| TCGA-AC-A8OS | TCGA-BRCA | Primary Solid tumor |
| TCGA-OK-A5Q2 | TCGA-BRCA | Primary Solid tumor |
| TCGA-B6-A1KF | TCGA-BRCA | Primary Solid tumor |
| TCGA-A8-A07F | TCGA-BRCA | Primary Solid tumor |
| TCGA-AR-A24L | TCGA-BRCA | Primary Solid tumor |
| TCGA-A2-A1FW | TCGA-BRCA | Primary Solid tumor |
| TCGA-A2-A3XU | TCGA-BRCA | Primary Solid tumor |
| TCGA-BH-A1FN | TCGA-BRCA | Primary Solid tumor |

| TCGA_patient_barcode | Project ID | Sample_type |
|---|---|---|
| TCGA-A8-A09X | TCGA-BRCA | Primary Solid tumor |
| TCGA-A7-A13D | TCGA-BRCA | Primary Solid tumor |
| TCGA-BH-A18S | TCGA-BRCA | Primary Solid tumor |
| TCGA-WT-AB44 | TCGA-BRCA | Primary Solid tumor |
| TCGA-EW-A1IW | TCGA-BRCA | Primary Solid tumor |
| TCGA-A2-A0EU | TCGA-BRCA | Primary Solid tumor |
| TCGA-UU-A93S | TCGA-BRCA | Primary Solid tumor |
| TCGA-A1-A0SN | TCGA-BRCA | Primary Solid tumor |
| TCGA-A7-A26E | TCGA-BRCA | Primary Solid tumor |
| TCGA-B6-A0I5 | TCGA-BRCA | Primary Solid tumor |
| TCGA-A8-A08A | TCGA-BRCA | Primary Solid tumor |
| TCGA-C8-A135 | TCGA-BRCA | Primary Solid tumor |
| TCGA-PL-A8LZ | TCGA-BRCA | Primary Solid tumor |
| TCGA-A2-A0YL | TCGA-BRCA | Primary Solid tumor |
| TCGA-B6-A0I9 | TCGA-BRCA | Primary Solid tumor |
| TCGA-A1-A0SD | TCGA-BRCA | Primary Solid tumor |
| TCGA-BH-A0AZ | TCGA-BRCA | Primary Solid tumor |
| TCGA-A2-A04W | TCGA-BRCA | Primary Solid tumor |
| TCGA-A2-A0ES | TCGA-BRCA | Primary Solid tumor |
| TCGA-AC-A8OQ | TCGA-BRCA | Primary Solid tumor |
| TCGA-A8-A0A4 | TCGA-BRCA | Primary Solid tumor |
| TCGA-AR-A251 | TCGA-BRCA | Primary Solid tumor |
| TCGA-HN-A2NL | TCGA-BRCA | Primary Solid tumor |
| TCGA-E2-A156 | TCGA-BRCA | Primary Solid tumor |
| TCGA-A8-A06T | TCGA-BRCA | Primary Solid tumor |
| TCGA-A2-A3XT | TCGA-BRCA | Primary Solid tumor |
| TCGA-C8-A12K | TCGA-BRCA | Primary Solid tumor |
| TCGA-D8-A1XU | TCGA-BRCA | Primary Solid tumor |
| TCGA-LL-A7T0 | TCGA-BRCA | Primary Solid tumor |
| TCGA-A2-A0D4 | TCGA-BRCA | Primary Solid tumor |
| TCGA-AO-A12E | TCGA-BRCA | Primary Solid tumor |
| TCGA-D8-A1XA | TCGA-BRCA | Primary Solid tumor |
| TCGA-C8-A3M7 | TCGA-BRCA | Primary Solid tumor |
| TCGA-E2-A10E | TCGA-BRCA | Primary Solid tumor |
| TCGA-D8-A1XO | TCGA-BRCA | Primary Solid tumor |
| TCGA-A8-A095 | TCGA-BRCA | Primary Solid tumor |
| TCGA-E9-A1RH | TCGA-BRCA | Primary Solid tumor |
| TCGA-A7-A0DB | TCGA-BRCA | Primary Solid tumor |
| TCGA-E9-A5UP | TCGA-BRCA | Primary Solid tumor |
| TCGA-D8-A1X8 | TCGA-BRCA | Primary Solid tumor |
| TCGA-AR-A5QQ | TCGA-BRCA | Primary Solid tumor |
| TCGA-AR-A255 | TCGA-BRCA | Primary Solid tumor |
| TCGA-A8-A09B | TCGA-BRCA | Primary Solid tumor |
| TCGA-AR-A24S | TCGA-BRCA | Primary Solid tumor |
| TCGA-A2-A0D3 | TCGA-BRCA | Primary Solid tumor |
| TCGA-AN-A0FD | TCGA-BRCA | Primary Solid tumor |

| TCGA_patient_barcode | Project ID | Sample_type |
|---|---|---|
| TCGA-3C-AALI | TCGA-BRCA | Primary Solid tumor |
| TCGA-LL-A5YP | TCGA-BRCA | Primary Solid tumor |
| TCGA-A2-A0CK | TCGA-BRCA | Primary Solid tumor |
| TCGA-C8-A278 | TCGA-BRCA | Primary Solid tumor |
| TCGA-A2-A0CU | TCGA-BRCA | Primary Solid tumor |
| TCGA-B6-A402 | TCGA-BRCA | Primary Solid tumor |
| TCGA-BH-A0HK | TCGA-BRCA | Primary Solid tumor |
| TCGA-E9-A54X | TCGA-BRCA | Primary Solid tumor |
| TCGA-E9-A249 | TCGA-BRCA | Primary Solid tumor |
| TCGA-D8-A27N | TCGA-BRCA | Primary Solid tumor |
| TCGA-BH-A0C3 | TCGA-BRCA | Primary Solid tumor |
| TCGA-AN-A0G0 | TCGA-BRCA | Primary Solid tumor |
| TCGA-A7-A13E | TCGA-BRCA | Primary Solid tumor |
| TCGA-A8-A08F | TCGA-BRCA | Primary Solid tumor |
| TCGA-B6-A0IO | TCGA-BRCA | Primary Solid tumor |
| TCGA-C8-A12Z | TCGA-BRCA | Primary Solid tumor |
| TCGA-B6-A0X4 | TCGA-BRCA | Primary Solid tumor |
| TCGA-LL-A6FR | TCGA-BRCA | Primary Solid tumor |
| TCGA-BH-A0BW | TCGA-BRCA | Primary Solid tumor |
| TCGA-E2-A15C | TCGA-BRCA | Primary Solid tumor |
| TCGA-A7-A13E | TCGA-BRCA | Primary Solid tumor |
| TCGA-AR-A1AX | TCGA-BRCA | Primary Solid tumor |
| TCGA-BH-A18I | TCGA-BRCA | Primary Solid tumor |
| TCGA-UL-AAZ6 | TCGA-BRCA | Primary Solid tumor |
| TCGA-B6-A0RI | TCGA-BRCA | Primary Solid tumor |
| TCGA-D8-A143 | TCGA-BRCA | Primary Solid tumor |
| TCGA-AR-A0TW | TCGA-BRCA | Primary Solid tumor |
| TCGA-AQ-A0Y5 | TCGA-BRCA | Primary Solid tumor |
| TCGA-E9-A1RB | TCGA-BRCA | Primary Solid tumor |
| TCGA-AO-A0JC | TCGA-BRCA | Primary Solid tumor |
| TCGA-BH-A0BJ | TCGA-BRCA | Primary Solid tumor |
| TCGA-AN-A0FX | TCGA-BRCA | Primary Solid tumor |
| TCGA-E9-A1NC | TCGA-BRCA | Primary Solid tumor |
| TCGA-AC-A3TM | TCGA-BRCA | Primary Solid tumor |
| TCGA-A8-A08Z | TCGA-BRCA | Primary Solid tumor |
| TCGA-BH-A0HX | TCGA-BRCA | Primary Solid tumor |
| TCGA-AR-A24X | TCGA-BRCA | Primary Solid tumor |
| TCGA-AO-A12A | TCGA-BRCA | Primary Solid tumor |
| TCGA-E9-A1NG | TCGA-BRCA | Primary Solid tumor |
| TCGA-AC-A3EH | TCGA-BRCA | Primary Solid tumor |
| TCGA-A7-A0DB | TCGA-BRCA | Primary Solid tumor |
| TCGA-A7-A4SF | TCGA-BRCA | Primary Solid tumor |
| TCGA-A8-A06U | TCGA-BRCA | Primary Solid tumor |
| TCGA-A7-A0DC | TCGA-BRCA | Primary Solid tumor |
| TCGA-A2-A0EX | TCGA-BRCA | Primary Solid tumor |
| TCGA-B6-A0RP | TCGA-BRCA | Primary Solid tumor |

| TCGA_patient_barcode | Project ID | Sample_type |
|---|---|---|
| TCGA-A2-A0T6 | TCGA-BRCA | Primary Solid tumor |
| TCGA-A2-A04U | TCGA-BRCA | Primary Solid tumor |
| TCGA-BH-A0HA | TCGA-BRCA | Primary Solid tumor |
| TCGA-A1-A0SQ | TCGA-BRCA | Primary Solid tumor |
| TCGA-D8-A1JK | TCGA-BRCA | Primary Solid tumor |
| TCGA-C8-A26W | TCGA-BRCA | Primary Solid tumor |
| TCGA-BH-A1F0 | TCGA-BRCA | Primary Solid tumor |
| TCGA-BH-A0EB | TCGA-BRCA | Primary Solid tumor |
| TCGA-AN-A049 | TCGA-BRCA | Primary Solid tumor |
| TCGA-A2-A0EV | TCGA-BRCA | Primary Solid tumor |
| TCGA-E2-A10C | TCGA-BRCA | Primary Solid tumor |
| TCGA-D8-A141 | TCGA-BRCA | Primary Solid tumor |
| TCGA-BH-A0B1 | TCGA-BRCA | Primary Solid tumor |
| TCGA-BH-A5IZ | TCGA-BRCA | Primary Solid tumor |
| TCGA-A8-A096 | TCGA-BRCA | Primary Solid tumor |
| TCGA-BH-A18K | TCGA-BRCA | Primary Solid tumor |
| TCGA-BH-A0DV | TCGA-BRCA | Primary Solid tumor |
| TCGA-PE-A5DC | TCGA-BRCA | Primary Solid tumor |
| TCGA-OL-A66K | TCGA-BRCA | Primary Solid tumor |
| TCGA-B6-A0X0 | TCGA-BRCA | Primary Solid tumor |
| TCGA-AC-A2B8 | TCGA-BRCA | Primary Solid tumor |
| TCGA-AO-A0JM | TCGA-BRCA | Primary Solid tumor |
| TCGA-E2-A1B0 | TCGA-BRCA | Primary Solid tumor |
| TCGA-E9-A229 | TCGA-BRCA | Primary Solid tumor |
| TCGA-A1-A0SI | TCGA-BRCA | Primary Solid tumor |
| TCGA-BH-A1F6 | TCGA-BRCA | Primary Solid tumor |
| TCGA-A8-A06Q | TCGA-BRCA | Primary Solid tumor |
| TCGA-A2-A04N | TCGA-BRCA | Primary Solid tumor |
| TCGA-AC-A2BK | TCGA-BRCA | Primary Solid tumor |
| TCGA-BH-A1EW | TCGA-BRCA | Primary Solid tumor |
| TCGA-C8-A27B | TCGA-BRCA | Primary Solid tumor |
| TCGA-AO-A1KO | TCGA-BRCA | Primary Solid tumor |
| TCGA-E2-A15M | TCGA-BRCA | Primary Solid tumor |
| TCGA-OL-A5RZ | TCGA-BRCA | Primary Solid tumor |
| TCGA-E2-A10B | TCGA-BRCA | Primary Solid tumor |
| TCGA-E9-A1N8 | TCGA-BRCA | Primary Solid tumor |
| TCGA-A2-A04P | TCGA-BRCA | Primary Solid tumor |
| TCGA-AN-A0AM | TCGA-BRCA | Primary Solid tumor |
| TCGA-AR-A24Z | TCGA-BRCA | Primary Solid tumor |
| TCGA-E2-A1B1 | TCGA-BRCA | Primary Solid tumor |
| TCGA-AO-A0JF | TCGA-BRCA | Primary Solid tumor |
| TCGA-A8-A07Z | TCGA-BRCA | Primary Solid tumor |
| TCGA-C8-A8HR | TCGA-BRCA | Primary Solid tumor |
| TCGA-A2-A0ER | TCGA-BRCA | Primary Solid tumor |
| TCGA-A8-A07U | TCGA-BRCA | Primary Solid tumor |
| TCGA-E2-A1LE | TCGA-BRCA | Primary Solid tumor |

| TCGA_patient_barcode | Project ID | Sample_type |
|---|---|---|
| TCGA-E9-A1NA | TCGA-BRCA | Primary Solid tumor |
| TCGA-D8-A1JG | TCGA-BRCA | Primary Solid tumor |
| TCGA-E2-A152 | TCGA-BRCA | Primary Solid tumor |
| TCGA-D8-A1X5 | TCGA-BRCA | Primary Solid tumor |
| TCGA-BH-A1FH | TCGA-BRCA | Primary Solid tumor |
| TCGA-AR-A2LL | TCGA-BRCA | Primary Solid tumor |
| TCGA-BH-A0EE | TCGA-BRCA | Primary Solid tumor |
| TCGA-A7-A4SD | TCGA-BRCA | Primary Solid tumor |
| TCGA-E2-A15G | TCGA-BRCA | Primary Solid tumor |
| TCGA-Z7-A8R5 | TCGA-BRCA | Primary Solid tumor |
| TCGA-BH-A0B8 | TCGA-BRCA | Primary Solid tumor |
| TCGA-A2-A0CL | TCGA-BRCA | Primary Solid tumor |
| TCGA-BH-A1FR | TCGA-BRCA | Primary Solid tumor |
| TCGA-BH-A0AY | TCGA-BRCA | Primary Solid tumor |
| TCGA-B6-A0WZ | TCGA-BRCA | Primary Solid tumor |
| TCGA-AR-A2LJ | TCGA-BRCA | Primary Solid tumor |
| TCGA-E9-A228 | TCGA-BRCA | Primary Solid tumor |
| TCGA-EW-A6SC | TCGA-BRCA | Primary Solid tumor |
| TCGA-D8-A145 | TCGA-BRCA | Primary Solid tumor |
| TCGA-XX-A89A | TCGA-BRCA | Primary Solid tumor |
| TCGA-AR-A0TY | TCGA-BRCA | Primary Solid tumor |
| TCGA-AO-A0J3 | TCGA-BRCA | Primary Solid tumor |
| TCGA-BH-A0C0 | TCGA-BRCA | Primary Solid tumor |
| TCGA-AR-A0TU | TCGA-BRCA | Primary Solid tumor |
| TCGA-BH-A0WA | TCGA-BRCA | Primary Solid tumor |
| TCGA-BH-A1FJ | TCGA-BRCA | Primary Solid tumor |
| TCGA-E2-A1IJ | TCGA-BRCA | Primary Solid tumor |
| TCGA-AR-A24P | TCGA-BRCA | Primary Solid tumor |
| TCGA-AN-A0XR | TCGA-BRCA | Primary Solid tumor |
| TCGA-A7-A425 | TCGA-BRCA | Primary Solid tumor |
| TCGA-D8-A1XY | TCGA-BRCA | Primary Solid tumor |
| TCGA-AR-A1AO | TCGA-BRCA | Primary Solid tumor |
| TCGA-A1-A0SM | TCGA-BRCA | Primary Solid tumor |
| TCGA-A2-A3XV | TCGA-BRCA | Primary Solid tumor |
| TCGA-C8-A12T | TCGA-BRCA | Primary Solid tumor |
| TCGA-E2-A106 | TCGA-BRCA | Primary Solid tumor |
| TCGA-D8-A27R | TCGA-BRCA | Primary Solid tumor |
| TCGA-A8-A08X | TCGA-BRCA | Primary Solid tumor |
| TCGA-AR-A24N | TCGA-BRCA | Primary Solid tumor |
| TCGA-B6-A400 | TCGA-BRCA | Primary Solid tumor |
| TCGA-E9-A243 | TCGA-BRCA | Primary Solid tumor |
| TCGA-E9-A1RA | TCGA-BRCA | Primary Solid tumor |
| TCGA-D8-A142 | TCGA-BRCA | Primary Solid tumor |
| TCGA-A1-A0SE | TCGA-BRCA | Primary Solid tumor |
| TCGA-AO-A0J6 | TCGA-BRCA | Primary Solid tumor |
| TCGA-BH-A1EU | TCGA-BRCA | Primary Solid tumor |

| TCGA_patient_barcode | Project ID | Sample_type |
|---|---|---|
| TCGA-A2-A0CP | TCGA-BRCA | Primary Solid tumor |
| TCGA-A8-A0AB | TCGA-BRCA | Primary Solid tumor |
| TCGA-BH-A0DS | TCGA-BRCA | Primary Solid tumor |
| TCGA-A8-A09K | TCGA-BRCA | Primary Solid tumor |
| TCGA-AO-A1KQ | TCGA-BRCA | Primary Solid tumor |
| TCGA-C8-A138 | TCGA-BRCA | Primary Solid tumor |
| TCGA-BH-A0AW | TCGA-BRCA | Primary Solid tumor |
| TCGA-B6-A0X5 | TCGA-BRCA | Primary Solid tumor |
| TCGA-E2-A15E | TCGA-BRCA | Primary Solid tumor |
| TCGA-AC-A3YJ | TCGA-BRCA | Primary Solid tumor |
| TCGA-E2-A1IN | TCGA-BRCA | Primary Solid tumor |
| TCGA-AO-A0J5 | TCGA-BRCA | Primary Solid tumor |
| TCGA-D8-A1X7 | TCGA-BRCA | Primary Solid tumor |
| TCGA-E2-A15O | TCGA-BRCA | Primary Solid tumor |
| TCGA-C8-A12V | TCGA-BRCA | Primary Solid tumor |
| TCGA-AC-A6IX | TCGA-BRCA | Primary Solid tumor |
| TCGA-EW-A1PB | TCGA-BRCA | Primary Solid tumor |
| TCGA-E2-A14O | TCGA-BRCA | Primary Solid tumor |
| TCGA-A2-A0EM | TCGA-BRCA | Primary Solid tumor |
| TCGA-AR-A1AS | TCGA-BRCA | Primary Solid tumor |
| TCGA-LL-A5YN | TCGA-BRCA | Primary Solid tumor |
| TCGA-AO-A124 | TCGA-BRCA | Primary Solid tumor |
| TCGA-AR-A1AN | TCGA-BRCA | Primary Solid tumor |
| TCGA-BH-A6R8 | TCGA-BRCA | Primary Solid tumor |
| TCGA-BH-A1FE | TCGA-BRCA | Primary Solid tumor |
| TCGA-A8-A09A | TCGA-BRCA | Primary Solid tumor |
| TCGA-D8-A1Y0 | TCGA-BRCA | Primary Solid tumor |
| TCGA-A8-A07G | TCGA-BRCA | Primary Solid tumor |
| TCGA-B6-A1KI | TCGA-BRCA | Primary Solid tumor |
| TCGA-S3-AA15 | TCGA-BRCA | Primary Solid tumor |
| TCGA-E2-A1IO | TCGA-BRCA | Primary Solid tumor |
| TCGA-E9-A1R4 | TCGA-BRCA | Primary Solid tumor |
| TCGA-A2-A0EP | TCGA-BRCA | Primary Solid tumor |
| TCGA-BH-A0BA | TCGA-BRCA | Primary Solid tumor |
| TCGA-B6-A0WV | TCGA-BRCA | Primary Solid tumor |
| TCGA-A8-A076 | TCGA-BRCA | Primary Solid tumor |
| TCGA-BH-A0EI | TCGA-BRCA | Primary Solid tumor |
| TCGA-AN-A0XT | TCGA-BRCA | Primary Solid tumor |
| TCGA-GM-A2DA | TCGA-BRCA | Primary Solid tumor |
| TCGA-C8-A134 | TCGA-BRCA | Primary Solid tumor |
| TCGA-E2-A15T | TCGA-BRCA | Primary Solid tumor |
| TCGA-A8-A07O | TCGA-BRCA | Primary Solid tumor |
| TCGA-AN-A0AJ | TCGA-BRCA | Primary Solid tumor |
| TCGA-AR-A254 | TCGA-BRCA | Primary Solid tumor |
| TCGA-AO-A03U | TCGA-BRCA | Primary Solid tumor |
| TCGA-B6-A0IP | TCGA-BRCA | Primary Solid tumor |

| TCGA_patient_barcode | Project ID | Sample_type |
| --- | --- | --- |
| TCGA-EW-A1P8 | TCGA-BRCA | Primary Solid tumor |
| TCGA-S3-AA10 | TCGA-BRCA | Primary Solid tumor |
| TCGA-MS-A51U | TCGA-BRCA | Primary Solid tumor |
| TCGA-EW-A6SA | TCGA-BRCA | Primary Solid tumor |
| TCGA-AR-A24W | TCGA-BRCA | Primary Solid tumor |
| TCGA-C8-A12Y | TCGA-BRCA | Primary Solid tumor |
| TCGA-BH-A1EX | TCGA-BRCA | Primary Solid tumor |
| TCGA-BH-A1FL | TCGA-BRCA | Primary Solid tumor |
| TCGA-BH-A0DE | TCGA-BRCA | Primary Solid tumor |
| TCGA-JL-A3YX | TCGA-BRCA | Primary Solid tumor |
| TCGA-BH-A5J0 | TCGA-BRCA | Primary Solid tumor |
| TCGA-A7-A13D | TCGA-BRCA | Primary Solid tumor |
| TCGA-E9-A2JS | TCGA-BRCA | Primary Solid tumor |
| TCGA-AO-A125 | TCGA-BRCA | Primary Solid tumor |
| TCGA-OL-A5RV | TCGA-BRCA | Primary Solid tumor |
| TCGA-A8-A081 | TCGA-BRCA | Primary Solid tumor |
| TCGA-A2-A0D2 | TCGA-BRCA | Primary Solid tumor |
| TCGA-BH-A0HQ | TCGA-BRCA | Primary Solid tumor |
| TCGA-AR-A1AQ | TCGA-BRCA | Primary Solid tumor |
| TCGA-A2-A0YD | TCGA-BRCA | Primary Solid tumor |
| TCGA-BH-A0DG | TCGA-BRCA | Primary Solid tumor |
| TCGA-AR-A256 | TCGA-BRCA | Primary Solid tumor |
| TCGA-AO-A0JI | TCGA-BRCA | Primary Solid tumor |
| TCGA-EW-A1PE | TCGA-BRCA | Primary Solid tumor |
| TCGA-AO-A1KT | TCGA-BRCA | Primary Solid tumor |
| TCGA-A7-A26G | TCGA-BRCA | Primary Solid tumor |
| TCGA-EW-A1IZ | TCGA-BRCA | Primary Solid tumor |
| TCGA-S3-A6ZH | TCGA-BRCA | Primary Solid tumor |
| TCGA-AR-A1AP | TCGA-BRCA | Primary Solid tumor |
| TCGA-A7-A56D | TCGA-BRCA | Primary Solid tumor |
| TCGA-BH-A0W5 | TCGA-BRCA | Primary Solid tumor |
| TCGA-A8-A099 | TCGA-BRCA | Primary Solid tumor |
| TCGA-OL-A97C | TCGA-BRCA | Primary Solid tumor |
| TCGA-OL-A66L | TCGA-BRCA | Primary Solid tumor |
| TCGA-A7-A3J1 | TCGA-BRCA | Primary Solid tumor |
| TCGA-AN-A0FL | TCGA-BRCA | Primary Solid tumor |
| TCGA-B6-A0IA | TCGA-BRCA | Primary Solid tumor |
| TCGA-AO-A0JL | TCGA-BRCA | Primary Solid tumor |
| TCGA-E9-A22A | TCGA-BRCA | Primary Solid tumor |
| TCGA-BH-A18H | TCGA-BRCA | Primary Solid tumor |
| TCGA-A8-A07R | TCGA-BRCA | Primary Solid tumor |
| TCGA-D8-A1J9 | TCGA-BRCA | Primary Solid tumor |
| TCGA-AN-A0XO | TCGA-BRCA | Primary Solid tumor |
| TCGA-AC-A4ZE | TCGA-BRCA | Primary Solid tumor |
| TCGA-PL-A8LV | TCGA-BRCA | Primary Solid tumor |
| TCGA-EW-A1IX | TCGA-BRCA | Primary Solid tumor |

| TCGA_patient_barcode | Project ID | Sample_type |
|---|---|---|
| TCGA-A2-A0YH | TCGA-BRCA | Primary Solid tumor |
| TCGA-EW-A1PF | TCGA-BRCA | Primary Solid tumor |
| TCGA-E2-A1L6 | TCGA-BRCA | Primary Solid tumor |
| TCGA-AN-A0FZ | TCGA-BRCA | Primary Solid tumor |
| TCGA-BH-A0HF | TCGA-BRCA | Primary Solid tumor |
| TCGA-GM-A5PV | TCGA-BRCA | Primary Solid tumor |
| TCGA-A2-A1G1 | TCGA-BRCA | Primary Solid tumor |
| TCGA-A2-A0CS | TCGA-BRCA | Primary Solid tumor |
| TCGA-EW-A1PC | TCGA-BRCA | Primary Solid tumor |
| TCGA-AO-A03T | TCGA-BRCA | Primary Solid tumor |
| TCGA-E2-A1LS | TCGA-BRCA | Primary Solid tumor |
| TCGA-AR-A2LE | TCGA-BRCA | Primary Solid tumor |
| TCGA-A7-A26E | TCGA-BRCA | Primary Solid tumor |
| TCGA-BH-A0BR | TCGA-BRCA | Primary Solid tumor |
| TCGA-A8-A06X | TCGA-BRCA | Primary Solid tumor |
| TCGA-E9-A248 | TCGA-BRCA | Primary Solid tumor |
| TCGA-A2-A0YE | TCGA-BRCA | Primary Solid tumor |
| TCGA-A1-A0SF | TCGA-BRCA | Primary Solid tumor |
| TCGA-BH-A0BO | TCGA-BRCA | Primary Solid tumor |
| TCGA-A8-A09T | TCGA-BRCA | Primary Solid tumor |
| TCGA-EW-A1J5 | TCGA-BRCA | Primary Solid tumor |
| TCGA-E2-A2P5 | TCGA-BRCA | Primary Solid tumor |
| TCGA-A8-A0A1 | TCGA-BRCA | Primary Solid tumor |
| TCGA-EW-A2FR | TCGA-BRCA | Primary Solid tumor |
| TCGA-AO-A03N | TCGA-BRCA | Primary Solid tumor |
| TCGA-B6-A3ZX | TCGA-BRCA | Primary Solid tumor |
| TCGA-5L-AAT0 | TCGA-BRCA | Primary Solid tumor |
| TCGA-AQ-A04L | TCGA-BRCA | Primary Solid tumor |
| TCGA-AO-A12F | TCGA-BRCA | Primary Solid tumor |
| TCGA-A8-A07L | TCGA-BRCA | Primary Solid tumor |
| TCGA-AN-A0XV | TCGA-BRCA | Primary Solid tumor |
| TCGA-AO-A0JB | TCGA-BRCA | Primary Solid tumor |
| TCGA-BH-A0B7 | TCGA-BRCA | Primary Solid tumor |
| TCGA-E9-A1NH | TCGA-BRCA | Primary Solid tumor |
| TCGA-BH-A0BF | TCGA-BRCA | Primary Solid tumor |
| TCGA-AC-A62Y | TCGA-BRCA | Primary Solid tumor |
| TCGA-AR-A0TZ | TCGA-BRCA | Primary Solid tumor |
| TCGA-EW-A423 | TCGA-BRCA | Primary Solid tumor |
| TCGA-A2-A0EQ | TCGA-BRCA | Primary Solid tumor |
| TCGA-OL-A5D6 | TCGA-BRCA | Primary Solid tumor |
| TCGA-BH-A0AV | TCGA-BRCA | Primary Solid tumor |
| TCGA-A7-A0CG | TCGA-BRCA | Primary Solid tumor |
| TCGA-D8-A27G | TCGA-BRCA | Primary Solid tumor |
| TCGA-EW-A1J6 | TCGA-BRCA | Primary Solid tumor |
| TCGA-A2-A1FZ | TCGA-BRCA | Primary Solid tumor |
| TCGA-E9-A5UO | TCGA-BRCA | Primary Solid tumor |

| TCGA_patient_barcode | Project ID | Sample_type |
|---|---|---|
| TCGA-BH-A0E2 | TCGA-BRCA | Primary Solid tumor |
| TCGA-A2-A4S1 | TCGA-BRCA | Primary Solid tumor |
| TCGA-AC-A7VB | TCGA-BRCA | Primary Solid tumor |
| TCGA-AC-A3QQ | TCGA-BRCA | Primary Solid tumor |
| TCGA-EW-A2FS | TCGA-BRCA | Primary Solid tumor |
| TCGA-BH-A1FU | TCGA-BRCA | Primary Solid tumor |
| TCGA-C8-A1HO | TCGA-BRCA | Primary Solid tumor |
| TCGA-AN-A04C | TCGA-BRCA | Primary Solid tumor |
| TCGA-A8-A0A9 | TCGA-BRCA | Primary Solid tumor |
| TCGA-BH-A0DO | TCGA-BRCA | Primary Solid tumor |
| TCGA-E2-A155 | TCGA-BRCA | Primary Solid tumor |
| TCGA-A1-A0SP | TCGA-BRCA | Primary Solid tumor |
| TCGA-A2-A25D | TCGA-BRCA | Primary Solid tumor |
| TCGA-BH-A202 | TCGA-BRCA | Primary Solid tumor |
| TCGA-D8-A1JS | TCGA-BRCA | Primary Solid tumor |
| TCGA-Z7-A8R6 | TCGA-BRCA | Primary Solid tumor |
| TCGA-D8-A1XF | TCGA-BRCA | Primary Solid tumor |
| TCGA-GM-A2DN | TCGA-BRCA | Primary Solid tumor |
| TCGA-BH-A0BC | TCGA-BRCA | Primary Solid tumor |
| TCGA-A2-A04X | TCGA-BRCA | Primary Solid tumor |
| TCGA-E9-A1RC | TCGA-BRCA | Primary Solid tumor |
| TCGA-AO-A1KR | TCGA-BRCA | Primary Solid tumor |
| TCGA-EW-A1OV | TCGA-BRCA | Primary Solid tumor |
| TCGA-C8-A8HQ | TCGA-BRCA | Primary Solid tumor |
| TCGA-OL-A66O | TCGA-BRCA | Primary Solid tumor |
| TCGA-PL-A8LX | TCGA-BRCA | Primary Solid tumor |
| TCGA-A8-A09V | TCGA-BRCA | Primary Solid tumor |
| TCGA-AR-A5QM | TCGA-BRCA | Primary Solid tumor |
| TCGA-A7-A2KD | TCGA-BRCA | Primary Solid tumor |
| TCGA-AQ-A7U7 | TCGA-BRCA | Primary Solid tumor |
| TCGA-BH-A0H7 | TCGA-BRCA | Primary Solid tumor |
| TCGA-LL-A73Y | TCGA-BRCA | Primary Solid tumor |
| TCGA-EW-A1PA | TCGA-BRCA | Primary Solid tumor |
| TCGA-A8-A0A7 | TCGA-BRCA | Primary Solid tumor |
| TCGA-A8-A08B | TCGA-BRCA | Primary Solid tumor |
| TCGA-E2-A154 | TCGA-BRCA | Primary Solid tumor |
| TCGA-A8-A07W | TCGA-BRCA | Primary Solid tumor |
| TCGA-GI-A2C9 | TCGA-BRCA | Primary Solid tumor |
| TCGA-AR-A24R | TCGA-BRCA | Primary Solid tumor |
| TCGA-D8-A1JU | TCGA-BRCA | Primary Solid tumor |
| TCGA-BH-A0H0 | TCGA-BRCA | Primary Solid tumor |
| TCGA-EW-A1PD | TCGA-BRCA | Primary Solid tumor |
| TCGA-E2-A572 | TCGA-BRCA | Primary Solid tumor |
| TCGA-C8-A133 | TCGA-BRCA | Primary Solid tumor |
| TCGA-EW-A1P6 | TCGA-BRCA | Primary Solid tumor |
| TCGA-AC-A2FF | TCGA-BRCA | Primary Solid tumor |

| **TCGA_patient_barcode** | **Project ID** | **Sample_type** |
|---|---|---|
| TCGA-WT-AB41 | TCGA-BRCA | Primary Solid tumor |
| TCGA-C8-A12Q | TCGA-BRCA | Primary Solid tumor |
| TCGA-E9-A1RG | TCGA-BRCA | Primary Solid tumor |
| TCGA-GM-A4E0 | TCGA-BRCA | Primary Solid tumor |
| TCGA-C8-A8HP | TCGA-BRCA | Primary Solid tumor |
| TCGA-AC-A23H | TCGA-BRCA | Primary Solid tumor |
| TCGA-BH-A0BM | TCGA-BRCA | Primary Solid tumor |
| TCGA-A8-A079 | TCGA-BRCA | Primary Solid tumor |
| TCGA-AR-A1AT | TCGA-BRCA | Primary Solid tumor |
| TCGA-A2-A0YG | TCGA-BRCA | Primary Solid tumor |
| TCGA-AC-A6IV | TCGA-BRCA | Primary Solid tumor |
| TCGA-BH-A18Q | TCGA-BRCA | Primary Solid tumor |
| TCGA-E2-A10F | TCGA-BRCA | Primary Solid tumor |
| TCGA-D8-A1JP | TCGA-BRCA | Primary Solid tumor |
| TCGA-C8-A1HF | TCGA-BRCA | Primary Solid tumor |
| TCGA-E2-A1B4 | TCGA-BRCA | Primary Solid tumor |
| TCGA-D8-A146 | TCGA-BRCA | Primary Solid tumor |
| TCGA-AR-A5QP | TCGA-BRCA | Primary Solid tumor |
| TCGA-A8-A08L | TCGA-BRCA | Primary Solid tumor |
| TCGA-AN-A0AT | TCGA-BRCA | Primary Solid tumor |
| TCGA-BH-A0BZ | TCGA-BRCA | Primary Solid tumor |
| TCGA-BH-A0HP | TCGA-BRCA | Primary Solid tumor |
| TCGA-A8-A06R | TCGA-BRCA | Primary Solid tumor |
| TCGA-A8-A08G | TCGA-BRCA | Primary Solid tumor |
| TCGA-A2-A0SV | TCGA-BRCA | Primary Solid tumor |
| TCGA-A2-A1G6 | TCGA-BRCA | Primary Solid tumor |
| TCGA-A2-A0YC | TCGA-BRCA | Primary Solid tumor |
| TCGA-D8-A1XV | TCGA-BRCA | Primary Solid tumor |
| TCGA-EW-A3U0 | TCGA-BRCA | Primary Solid tumor |
| TCGA-A2-A0SU | TCGA-BRCA | Primary Solid tumor |
| TCGA-A2-A4RX | TCGA-BRCA | Primary Solid tumor |
| TCGA-E9-A1N9 | TCGA-BRCA | Primary Solid tumor |
| TCGA-B6-A40C | TCGA-BRCA | Primary Solid tumor |
| TCGA-A8-A09N | TCGA-BRCA | Primary Solid tumor |
| TCGA-AO-A1KP | TCGA-BRCA | Primary Solid tumor |
| TCGA-AC-A7VC | TCGA-BRCA | Primary Solid tumor |
| TCGA-AO-A0JJ | TCGA-BRCA | Primary Solid tumor |
| TCGA-GM-A5PX | TCGA-BRCA | Primary Solid tumor |
| TCGA-AN-A0FT | TCGA-BRCA | Primary Solid tumor |
| TCGA-D8-A27V | TCGA-BRCA | Primary Solid tumor |
| TCGA-B6-A0I1 | TCGA-BRCA | Primary Solid tumor |
| TCGA-A8-A07C | TCGA-BRCA | Primary Solid tumor |
| TCGA-D8-A1JL | TCGA-BRCA | Primary Solid tumor |
| TCGA-D8-A27E | TCGA-BRCA | Primary Solid tumor |
| TCGA-AQ-A54N | TCGA-BRCA | Primary Solid tumor |
| TCGA-AR-A0TP | TCGA-BRCA | Primary Solid tumor |

| TCGA_patient_barcode | Project ID | Sample_type |
|---|---|---|
| TCGA-AO-A0JD | TCGA-BRCA | Primary Solid tumor |
| TCGA-C8-A27A | TCGA-BRCA | Primary Solid tumor |
| TCGA-BH-A1F8 | TCGA-BRCA | Primary Solid tumor |
| TCGA-A8-A090 | TCGA-BRCA | Primary Solid tumor |
| TCGA-BH-A0DT | TCGA-BRCA | Primary Solid tumor |
| TCGA-A8-A07E | TCGA-BRCA | Primary Solid tumor |
| TCGA-A8-A0A6 | TCGA-BRCA | Primary Solid tumor |
| TCGA-AR-A2LN | TCGA-BRCA | Primary Solid tumor |
| TCGA-E2-A1IG | TCGA-BRCA | Primary Solid tumor |
| TCGA-E2-A1LB | TCGA-BRCA | Primary Solid tumor |
| TCGA-EW-A1PH | TCGA-BRCA | Primary Solid tumor |
| TCGA-A7-A6VY | TCGA-BRCA | Primary Solid tumor |
| TCGA-BH-A0C1 | TCGA-BRCA | Primary Solid tumor |
| TCGA-AR-A1AR | TCGA-BRCA | Primary Solid tumor |
| TCGA-A7-A3IY | TCGA-BRCA | Primary Solid tumor |
| TCGA-AN-A0FV | TCGA-BRCA | Primary Solid tumor |
| TCGA-A7-A4SA | TCGA-BRCA | Primary Solid tumor |
| TCGA-AR-A24V | TCGA-BRCA | Primary Solid tumor |
| TCGA-AO-A0J2 | TCGA-BRCA | Primary Solid tumor |
| TCGA-E9-A244 | TCGA-BRCA | Primary Solid tumor |
| TCGA-A2-A0CT | TCGA-BRCA | Primary Solid tumor |
| TCGA-BH-A0BD | TCGA-BRCA | Primary Solid tumor |
| TCGA-LL-A5YL | TCGA-BRCA | Primary Solid tumor |
| TCGA-D8-A1XR | TCGA-BRCA | Primary Solid tumor |
| TCGA-GM-A3NW | TCGA-BRCA | Primary Solid tumor |
| TCGA-C8-A12M | TCGA-BRCA | Primary Solid tumor |
| TCGA-E2-A1BD | TCGA-BRCA | Primary Solid tumor |
| TCGA-AO-A12G | TCGA-BRCA | Primary Solid tumor |
| TCGA-LD-A9QF | TCGA-BRCA | Primary Solid tumor |
| TCGA-LD-A7W6 | TCGA-BRCA | Primary Solid tumor |
| TCGA-BH-A0BL | TCGA-BRCA | Primary Solid tumor |
| TCGA-BH-A0B2 | TCGA-BRCA | Primary Solid tumor |
| TCGA-AN-A0XN | TCGA-BRCA | Primary Solid tumor |
| TCGA-B6-A0WX | TCGA-BRCA | Primary Solid tumor |
| TCGA-AC-A23C | TCGA-BRCA | Primary Solid tumor |
| TCGA-E2-A14W | TCGA-BRCA | Primary Solid tumor |
| TCGA-E9-A1R3 | TCGA-BRCA | Primary Solid tumor |
| TCGA-D8-A1XK | TCGA-BRCA | Primary Solid tumor |
| TCGA-E2-A1LI | TCGA-BRCA | Primary Solid tumor |
| TCGA-AC-A6NO | TCGA-BRCA | Primary Solid tumor |
| TCGA-BH-A42V | TCGA-BRCA | Primary Solid tumor |
| TCGA-AC-A3W7 | TCGA-BRCA | Primary Solid tumor |
| TCGA-OL-A66N | TCGA-BRCA | Primary Solid tumor |
| TCGA-A7-A26H | TCGA-BRCA | Primary Solid tumor |
| TCGA-BH-A0HW | TCGA-BRCA | Primary Solid tumor |
| TCGA-AR-A0U2 | TCGA-BRCA | Primary Solid tumor |

| TCGA_patient_barcode | Project ID | Sample_type |
|---|---|---|
| TCGA-E2-A1B6 | TCGA-BRCA | Primary Solid tumor |
| TCGA-OL-A5RU | TCGA-BRCA | Primary Solid tumor |
| TCGA-A8-A06O | TCGA-BRCA | Primary Solid tumor |
| TCGA-A8-A08S | TCGA-BRCA | Primary Solid tumor |
| TCGA-BH-A0HY | TCGA-BRCA | Primary Solid tumor |
| TCGA-A2-A0CQ | TCGA-BRCA | Primary Solid tumor |
| TCGA-EW-A6S9 | TCGA-BRCA | Primary Solid tumor |
| TCGA-BH-A209 | TCGA-BRCA | Primary Solid tumor |
| TCGA-BH-A0B5 | TCGA-BRCA | Primary Solid tumor |
| TCGA-3C-AALK | TCGA-BRCA | Primary Solid tumor |
| TCGA-A2-A0CZ | TCGA-BRCA | Primary Solid tumor |
| TCGA-D8-A1X6 | TCGA-BRCA | Primary Solid tumor |
| TCGA-A2-A0ST | TCGA-BRCA | Primary Solid tumor |
| TCGA-LL-A441 | TCGA-BRCA | Primary Solid tumor |
| TCGA-AR-A24U | TCGA-BRCA | Primary Solid tumor |
| TCGA-AR-A24T | TCGA-BRCA | Primary Solid tumor |
| TCGA-E9-A2JT | TCGA-BRCA | Primary Solid tumor |
| TCGA-E9-A1R6 | TCGA-BRCA | Primary Solid tumor |
| TCGA-BH-A2L8 | TCGA-BRCA | Primary Solid tumor |
| TCGA-OL-A6VQ | TCGA-BRCA | Primary Solid tumor |
| TCGA-BH-A0RX | TCGA-BRCA | Primary Solid tumor |
| TCGA-A2-A3XW | TCGA-BRCA | Primary Solid tumor |
| TCGA-E2-A15I | TCGA-BRCA | Primary Solid tumor |
| TCGA-E9-A227 | TCGA-BRCA | Primary Solid tumor |
| TCGA-A2-A4RY | TCGA-BRCA | Primary Solid tumor |
| TCGA-C8-A12L | TCGA-BRCA | Primary Solid tumor |
| TCGA-BH-A0H6 | TCGA-BRCA | Primary Solid tumor |
| TCGA-B6-A0IE | TCGA-BRCA | Primary Solid tumor |
| TCGA-BH-A201 | TCGA-BRCA | Primary Solid tumor |
| TCGA-A2-A04R | TCGA-BRCA | Primary Solid tumor |
| TCGA-AR-A1AJ | TCGA-BRCA | Primary Solid tumor |
| TCGA-D8-A1JN | TCGA-BRCA | Primary Solid tumor |
| TCGA-A8-A083 | TCGA-BRCA | Primary Solid tumor |
| TCGA-A8-A09M | TCGA-BRCA | Primary Solid tumor |
| TCGA-E9-A54Y | TCGA-BRCA | Primary Solid tumor |
| TCGA-B6-A0IN | TCGA-BRCA | Primary Solid tumor |
| TCGA-EW-A1J1 | TCGA-BRCA | Primary Solid tumor |
| TCGA-5L-AAT1 | TCGA-BRCA | Primary Solid tumor |
| TCGA-A7-A6VV | TCGA-BRCA | Primary Solid tumor |
| TCGA-B6-A409 | TCGA-BRCA | Primary Solid tumor |
| TCGA-A2-A0SW | TCGA-BRCA | Primary Solid tumor |
| TCGA-E9-A1N3 | TCGA-BRCA | Primary Solid tumor |
| TCGA-BH-A0BT | TCGA-BRCA | Primary Solid tumor |
| TCGA-A2-A25B | TCGA-BRCA | Primary Solid tumor |
| TCGA-A8-A091 | TCGA-BRCA | Primary Solid tumor |
| TCGA-B6-A0IJ | TCGA-BRCA | Primary Solid tumor |

| TCGA_patient_barcode | Project ID | Sample_type |
|---|---|---|
| TCGA-A2-A0YT | TCGA-BRCA | Primary Solid tumor |
| TCGA-A8-A09I | TCGA-BRCA | Primary Solid tumor |
| TCGA-GM-A2DL | TCGA-BRCA | Primary Solid tumor |
| TCGA-GM-A2DC | TCGA-BRCA | Primary Solid tumor |
| TCGA-AR-A1AY | TCGA-BRCA | Primary Solid tumor |
| TCGA-BH-A28Q | TCGA-BRCA | Primary Solid tumor |
| TCGA-A2-A3XX | TCGA-BRCA | Primary Solid tumor |
| TCGA-A7-A5ZV | TCGA-BRCA | Primary Solid tumor |
| TCGA-XX-A899 | TCGA-BRCA | Primary Solid tumor |
| TCGA-E2-A1AZ | TCGA-BRCA | Primary Solid tumor |
| TCGA-A8-A06N | TCGA-BRCA | Primary Solid tumor |
| TCGA-E9-A1NE | TCGA-BRCA | Primary Solid tumor |
| TCGA-BH-A8FZ | TCGA-BRCA | Primary Solid tumor |
| TCGA-D8-A1XT | TCGA-BRCA | Primary Solid tumor |
| TCGA-E9-A22E | TCGA-BRCA | Primary Solid tumor |
| TCGA-3C-AAAU | TCGA-BRCA | Primary Solid tumor |
| TCGA-S3-AA12 | TCGA-BRCA | Primary Solid tumor |
| TCGA-A7-A6VX | TCGA-BRCA | Primary Solid tumor |
| TCGA-A8-A08R | TCGA-BRCA | Primary Solid tumor |
| TCGA-HN-A2OB | TCGA-BRCA | Primary Solid tumor |
| TCGA-A7-A13D | TCGA-BRCA | Primary Solid tumor |
| TCGA-B6-A0IK | TCGA-BRCA | Primary Solid tumor |
| TCGA-A7-A4SC | TCGA-BRCA | Primary Solid tumor |
| TCGA-BH-A0HU | TCGA-BRCA | Primary Solid tumor |
| TCGA-BH-A1FD | TCGA-BRCA | Primary Solid tumor |
| TCGA-BH-A0E0 | TCGA-BRCA | Primary Solid tumor |
| TCGA-A2-A25C | TCGA-BRCA | Primary Solid tumor |
| TCGA-AN-A0XW | TCGA-BRCA | Primary Solid tumor |
| TCGA-D8-A1Y1 | TCGA-BRCA | Primary Solid tumor |
| TCGA-E2-A15R | TCGA-BRCA | Primary Solid tumor |
| TCGA-D8-A1Y2 | TCGA-BRCA | Primary Solid tumor |
| TCGA-C8-A1HI | TCGA-BRCA | Primary Solid tumor |
| TCGA-A2-A0ET | TCGA-BRCA | Primary Solid tumor |
| TCGA-AR-A1AV | TCGA-BRCA | Primary Solid tumor |
| TCGA-A2-A4S3 | TCGA-BRCA | Primary Solid tumor |
| TCGA-B6-A0IQ | TCGA-BRCA | Primary Solid tumor |
| TCGA-A2-A0CX | TCGA-BRCA | Primary Solid tumor |
| TCGA-A7-A3RF | TCGA-BRCA | Primary Solid tumor |
| TCGA-LL-A8F5 | TCGA-BRCA | Primary Solid tumor |
| TCGA-EW-A1J2 | TCGA-BRCA | Primary Solid tumor |
| TCGA-LD-A74U | TCGA-BRCA | Primary Solid tumor |
| TCGA-AR-A1AH | TCGA-BRCA | Primary Solid tumor |
| TCGA-E9-A1QZ | TCGA-BRCA | Primary Solid tumor |
| TCGA-E9-A1RF | TCGA-BRCA | Primary Solid tumor |
| TCGA-B6-A0RM | TCGA-BRCA | Primary Solid tumor |
| TCGA-EW-A1P0 | TCGA-BRCA | Primary Solid tumor |

| TCGA_patient_barcode | Project ID | Sample_type |
|---|---|---|
| TCGA-E2-A1B5 | TCGA-BRCA | Primary Solid tumor |
| TCGA-AC-A3YI | TCGA-BRCA | Primary Solid tumor |
| TCGA-GM-A2D9 | TCGA-BRCA | Primary Solid tumor |
| TCGA-GM-A2DF | TCGA-BRCA | Primary Solid tumor |
| TCGA-D8-A1X9 | TCGA-BRCA | Primary Solid tumor |
| TCGA-AR-A2LM | TCGA-BRCA | Primary Solid tumor |
| TCGA-A7-A6VW | TCGA-BRCA | Primary Solid tumor |
| TCGA-AR-A24H | TCGA-BRCA | Primary Solid tumor |
| TCGA-AN-A041 | TCGA-BRCA | Primary Solid tumor |
| TCGA-AN-A0AS | TCGA-BRCA | Primary Solid tumor |
| TCGA-A8-A08J | TCGA-BRCA | Primary Solid tumor |
| TCGA-A2-A0CO | TCGA-BRCA | Primary Solid tumor |
| TCGA-A8-A08C | TCGA-BRCA | Primary Solid tumor |
| TCGA-A2-A3KC | TCGA-BRCA | Primary Solid tumor |
| TCGA-BH-A1EO | TCGA-BRCA | Primary Solid tumor |
| TCGA-BH-A1FM | TCGA-BRCA | Primary Solid tumor |
| TCGA-D8-A1JF | TCGA-BRCA | Primary Solid tumor |
| TCGA-E2-A14T | TCGA-BRCA | Primary Solid tumor |
| TCGA-A7-A26J | TCGA-BRCA | Primary Solid tumor |
| TCGA-A1-A0SJ | TCGA-BRCA | Primary Solid tumor |
| TCGA-B6-A0X7 | TCGA-BRCA | Primary Solid tumor |
| TCGA-AC-A62X | TCGA-BRCA | Primary Solid tumor |
| TCGA-C8-A273 | TCGA-BRCA | Primary Solid tumor |
| TCGA-BH-A0B0 | TCGA-BRCA | Primary Solid tumor |
| TCGA-BH-A1EN | TCGA-BRCA | Primary Solid tumor |
| TCGA-BH-A0C7 | TCGA-BRCA | Primary Solid tumor |
| TCGA-D8-A73U | TCGA-BRCA | Primary Solid tumor |
| TCGA-A8-A0AD | TCGA-BRCA | Primary Solid tumor |
| TCGA-BH-A28O | TCGA-BRCA | Primary Solid tumor |
| TCGA-S3-AA14 | TCGA-BRCA | Primary Solid tumor |
| TCGA-EW-A1OW | TCGA-BRCA | Primary Solid tumor |
| TCGA-BH-A0HB | TCGA-BRCA | Primary Solid tumor |
| TCGA-E2-A56Z | TCGA-BRCA | Primary Solid tumor |
| TCGA-S3-AA0Z | TCGA-BRCA | Primary Solid tumor |
| TCGA-B6-A0WT | TCGA-BRCA | Primary Solid tumor |
| TCGA-A7-A26J | TCGA-BRCA | Primary Solid tumor |
| TCGA-AO-A03M | TCGA-BRCA | Primary Solid tumor |
| TCGA-EW-A6SB | TCGA-BRCA | Primary Solid tumor |
| TCGA-A8-A09E | TCGA-BRCA | Primary Solid tumor |
| TCGA-AQ-A1H3 | TCGA-BRCA | Primary Solid tumor |
| TCGA-A8-A086 | TCGA-BRCA | Primary Solid tumor |
| TCGA-A2-A3KD | TCGA-BRCA | Primary Solid tumor |
| TCGA-BH-A0DP | TCGA-BRCA | Primary Solid tumor |
| TCGA-D8-A1XJ | TCGA-BRCA | Primary Solid tumor |
| TCGA-E2-A105 | TCGA-BRCA | Primary Solid tumor |
| TCGA-D8-A1JM | TCGA-BRCA | Primary Solid tumor |

| TCGA_patient_barcode | Project ID | Sample_type |
|---|---|---|
| TCGA-AR-A0TX | TCGA-BRCA | Primary Solid tumor |
| TCGA-A8-A09R | TCGA-BRCA | Primary Solid tumor |
| TCGA-A8-A08O | TCGA-BRCA | Primary Solid tumor |
| TCGA-AQ-A04H | TCGA-BRCA | Primary Solid tumor |
| TCGA-EW-A1P4 | TCGA-BRCA | Primary Solid tumor |
| TCGA-C8-A3M8 | TCGA-BRCA | Primary Solid tumor |
| TCGA-EW-A424 | TCGA-BRCA | Primary Solid tumor |
| TCGA-B6-A0IM | TCGA-BRCA | Primary Solid tumor |
| TCGA-E2-A1IH | TCGA-BRCA | Primary Solid tumor |
| TCGA-A2-A0YK | TCGA-BRCA | Primary Solid tumor |
| TCGA-AR-A0TT | TCGA-BRCA | Primary Solid tumor |
| TCGA-AC-A3BB | TCGA-BRCA | Primary Solid tumor |
| TCGA-A1-A0SG | TCGA-BRCA | Primary Solid tumor |
| TCGA-AR-A2LR | TCGA-BRCA | Primary Solid tumor |
| TCGA-AR-A0U3 | TCGA-BRCA | Primary Solid tumor |
| TCGA-GM-A3XG | TCGA-BRCA | Primary Solid tumor |
| TCGA-A2-A0T1 | TCGA-BRCA | Primary Solid tumor |
| TCGA-AO-A12B | TCGA-BRCA | Primary Solid tumor |
| TCGA-A8-A06Y | TCGA-BRCA | Primary Solid tumor |
| TCGA-A2-A3XS | TCGA-BRCA | Primary Solid tumor |
| TCGA-A2-A0T2 | TCGA-BRCA | Primary Solid tumor |
| TCGA-E9-A247 | TCGA-BRCA | Primary Solid tumor |
| TCGA-AC-A23E | TCGA-BRCA | Primary Solid tumor |
| TCGA-D8-A1XB | TCGA-BRCA | Primary Solid tumor |
| TCGA-E2-A150 | TCGA-BRCA | Primary Solid tumor |
| TCGA-C8-A12N | TCGA-BRCA | Primary Solid tumor |
| TCGA-B6-A0RG | TCGA-BRCA | Primary Solid tumor |
| TCGA-A2-A0YF | TCGA-BRCA | Primary Solid tumor |
| TCGA-C8-A1HJ | TCGA-BRCA | Primary Solid tumor |
| TCGA-AR-A1AW | TCGA-BRCA | Primary Solid tumor |
| TCGA-AO-A03O | TCGA-BRCA | Primary Solid tumor |
| TCGA-S3-AA17 | TCGA-BRCA | Primary Solid tumor |
| TCGA-A7-A4SB | TCGA-BRCA | Primary Solid tumor |
| TCGA-E9-A1N6 | TCGA-BRCA | Primary Solid tumor |
| TCGA-AO-A12D | TCGA-BRCA | Primary Solid tumor |
| TCGA-A7-A0CE | TCGA-BRCA | Primary Solid tumor |
| TCGA-E2-A14P | TCGA-BRCA | Primary Solid tumor |
| TCGA-D8-A27H | TCGA-BRCA | Primary Solid tumor |
| TCGA-B6-A0WS | TCGA-BRCA | Primary Solid tumor |
| TCGA-AN-A03X | TCGA-BRCA | Primary Solid tumor |
| TCGA-BH-A0DQ | TCGA-BRCA | Primary Solid tumor |
| TCGA-AO-A0JG | TCGA-BRCA | Primary Solid tumor |
| TCGA-E2-A1II | TCGA-BRCA | Primary Solid tumor |
| TCGA-AR-A0TR | TCGA-BRCA | Primary Solid tumor |
| TCGA-E9-A1NI | TCGA-BRCA | Primary Solid tumor |
| TCGA-E9-A1RI | TCGA-BRCA | Primary Solid tumor |

| TCGA_patient_barcode | Project ID | Sample_type |
|---|---|---|
| TCGA-AC-A5XS | TCGA-BRCA | Primary Solid tumor |
| TCGA-PE-A5DE | TCGA-BRCA | Primary Solid tumor |
| TCGA-AC-A3OD | TCGA-BRCA | Primary Solid tumor |
| TCGA-B6-A0RV | TCGA-BRCA | Primary Solid tumor |
| TCGA-E2-A15K | TCGA-BRCA | Primary Solid tumor |
| TCGA-A2-A0CM | TCGA-BRCA | Primary Solid tumor |
| TCGA-S3-AA11 | TCGA-BRCA | Primary Solid tumor |
| TCGA-D8-A27W | TCGA-BRCA | Primary Solid tumor |
| TCGA-BH-A0DZ | TCGA-BRCA | Primary Solid tumor |
| TCGA-C8-A1HG | TCGA-BRCA | Primary Solid tumor |
| TCGA-D8-A27I | TCGA-BRCA | Primary Solid tumor |
| TCGA-AN-A0AL | TCGA-BRCA | Primary Solid tumor |
| TCGA-AO-A03P | TCGA-BRCA | Primary Solid tumor |
| TCGA-A7-A0DA | TCGA-BRCA | Primary Solid tumor |
| TCGA-E2-A9RU | TCGA-BRCA | Primary Solid tumor |
| TCGA-E2-A14R | TCGA-BRCA | Primary Solid tumor |
| TCGA-GM-A2DK | TCGA-BRCA | Primary Solid tumor |
| TCGA-E2-A15S | TCGA-BRCA | Primary Solid tumor |
| TCGA-B6-A0IC | TCGA-BRCA | Primary Solid tumor |
| TCGA-A2-A1FX | TCGA-BRCA | Primary Solid tumor |
| TCGA-LL-A740 | TCGA-BRCA | Primary Solid tumor |
| TCGA-AN-A04D | TCGA-BRCA | Primary Solid tumor |
| TCGA-LL-A50Y | TCGA-BRCA | Primary Solid tumor |
| TCGA-B6-A0RE | TCGA-BRCA | Primary Solid tumor |
| TCGA-E2-A1LH | TCGA-BRCA | Primary Solid tumor |
| TCGA-C8-A137 | TCGA-BRCA | Primary Solid tumor |
| TCGA-A2-A25F | TCGA-BRCA | Primary Solid tumor |
| TCGA-LL-A5YM | TCGA-BRCA | Primary Solid tumor |
| TCGA-D8-A1JD | TCGA-BRCA | Primary Solid tumor |
| TCGA-A2-A0YM | TCGA-BRCA | Primary Solid tumor |
| TCGA-BH-A0BP | TCGA-BRCA | Primary Solid tumor |
| TCGA-AN-A0FK | TCGA-BRCA | Primary Solid tumor |
| TCGA-E2-A1IE | TCGA-BRCA | Primary Solid tumor |
| TCGA-AN-A03Y | TCGA-BRCA | Primary Solid tumor |
| TCGA-E2-A576 | TCGA-BRCA | Primary Solid tumor |
| TCGA-A2-A0SX | TCGA-BRCA | Primary Solid tumor |
| TCGA-C8-A12X | TCGA-BRCA | Primary Solid tumor |
| TCGA-A7-A26I | TCGA-BRCA | Primary Solid tumor |
| TCGA-BH-A0E6 | TCGA-BRCA | Primary Solid tumor |
| TCGA-A8-A07B | TCGA-BRCA | Primary Solid tumor |
| TCGA-E2-A159 | TCGA-BRCA | Primary Solid tumor |
| TCGA-A2-A04Y | TCGA-BRCA | Primary Solid tumor |
| TCGA-EW-A1OY | TCGA-BRCA | Primary Solid tumor |
| TCGA-E9-A5FK | TCGA-BRCA | Primary Solid tumor |
| TCGA-AO-A128 | TCGA-BRCA | Primary Solid tumor |
| TCGA-AR-A0TV | TCGA-BRCA | Primary Solid tumor |

| TCGA_patient_barcode | Project ID | Sample_type |
|---|---|---|
| TCGA-D8-A1J8 | TCGA-BRCA | Primary Solid tumor |
| TCGA-E2-A573 | TCGA-BRCA | Primary Solid tumor |
| TCGA-BH-A1FG | TCGA-BRCA | Primary Solid tumor |
| TCGA-BH-A204 | TCGA-BRCA | Primary Solid tumor |
| TCGA-E9-A1R0 | TCGA-BRCA | Primary Solid tumor |
| TCGA-E2-A158 | TCGA-BRCA | Primary Solid tumor |
| TCGA-EW-A3E8 | TCGA-BRCA | Primary Solid tumor |
| TCGA-GM-A2DM | TCGA-BRCA | Primary Solid tumor |
| TCGA-A8-A094 | TCGA-BRCA | Primary Solid tumor |
| TCGA-B6-A0IH | TCGA-BRCA | Primary Solid tumor |
| TCGA-E9-A22H | TCGA-BRCA | Primary Solid tumor |
| TCGA-AR-A250 | TCGA-BRCA | Primary Solid tumor |
| TCGA-E2-A1L7 | TCGA-BRCA | Primary Solid tumor |
| TCGA-E9-A1ND | TCGA-BRCA | Primary Solid tumor |
| TCGA-BH-A0E1 | TCGA-BRCA | Primary Solid tumor |
| TCGA-BH-A1ET | TCGA-BRCA | Primary Solid tumor |
| TCGA-AO-A03R | TCGA-BRCA | Primary Solid tumor |
| TCGA-C8-A12P | TCGA-BRCA | Primary Solid tumor |
| TCGA-A8-A08H | TCGA-BRCA | Primary Solid tumor |
| TCGA-A8-A08P | TCGA-BRCA | Primary Solid tumor |
| TCGA-A2-A04V | TCGA-BRCA | Primary Solid tumor |
| TCGA-BH-A0HO | TCGA-BRCA | Primary Solid tumor |
| TCGA-AC-A3TN | TCGA-BRCA | Primary Solid tumor |
| TCGA-OL-A6VR | TCGA-BRCA | Primary Solid tumor |
| TCGA-A8-A09W | TCGA-BRCA | Primary Solid tumor |
| TCGA-AC-A3W5 | TCGA-BRCA | Primary Solid tumor |
| TCGA-A8-A07I | TCGA-BRCA | Primary Solid tumor |
| TCGA-D8-A1JA | TCGA-BRCA | Primary Solid tumor |
| TCGA-A2-A04T | TCGA-BRCA | Primary Solid tumor |
| TCGA-AR-A24O | TCGA-BRCA | Primary Solid tumor |
| TCGA-5T-A9QA | TCGA-BRCA | Primary Solid tumor |
| TCGA-E9-A22B | TCGA-BRCA | Primary Solid tumor |
| TCGA-E2-A1L9 | TCGA-BRCA | Primary Solid tumor |
| TCGA-A2-A0T4 | TCGA-BRCA | Primary Solid tumor |
| TCGA-E9-A5FL | TCGA-BRCA | Primary Solid tumor |
| TCGA-AR-A2LH | TCGA-BRCA | Primary Solid tumor |
| TCGA-D8-A1JI | TCGA-BRCA | Primary Solid tumor |
| TCGA-C8-A1HM | TCGA-BRCA | Primary Solid tumor |
| TCGA-E9-A1N4 | TCGA-BRCA | Primary Solid tumor |
| TCGA-A1-A0SH | TCGA-BRCA | Primary Solid tumor |
| TCGA-B6-A0I2 | TCGA-BRCA | Primary Solid tumor |
| TCGA-S3-A6ZF | TCGA-BRCA | Primary Solid tumor |
| TCGA-E2-A15L | TCGA-BRCA | Primary Solid tumor |
| TCGA-D8-A1XC | TCGA-BRCA | Primary Solid tumor |
| TCGA-BH-A1FC | TCGA-BRCA | Primary Solid tumor |
| TCGA-E2-A108 | TCGA-BRCA | Primary Solid tumor |

| TCGA_patient_barcode | Project ID | Sample_type |
|---|---|---|
| TCGA-EW-A1P7 | TCGA-BRCA | Primary Solid tumor |
| TCGA-4H-AAAK | TCGA-BRCA | Primary Solid tumor |
| TCGA-E2-A153 | TCGA-BRCA | Primary Solid tumor |
| TCGA-C8-A12U | TCGA-BRCA | Primary Solid tumor |
| TCGA-AR-A24Q | TCGA-BRCA | Primary Solid tumor |
| TCGA-AR-A1AL | TCGA-BRCA | Primary Solid tumor |
| TCGA-AN-A0XU | TCGA-BRCA | Primary Solid tumor |
| TCGA-A2-A0CY | TCGA-BRCA | Primary Solid tumor |
| TCGA-AN-A046 | TCGA-BRCA | Primary Solid tumor |
| TCGA-BH-A42T | TCGA-BRCA | Primary Solid tumor |
| TCGA-BH-A208 | TCGA-BRCA | Primary Solid tumor |
| TCGA-A2-A25A | TCGA-BRCA | Primary Solid tumor |
| TCGA-B6-A408 | TCGA-BRCA | Primary Solid tumor |
| TCGA-A8-A075 | TCGA-BRCA | Primary Solid tumor |
| TCGA-E9-A3HO | TCGA-BRCA | Primary Solid tumor |
| TCGA-OL-A66J | TCGA-BRCA | Primary Solid tumor |
| TCGA-E2-A109 | TCGA-BRCA | Primary Solid tumor |
| TCGA-D8-A1XS | TCGA-BRCA | Primary Solid tumor |
| TCGA-AC-A3W6 | TCGA-BRCA | Primary Solid tumor |
| TCGA-BH-A18P | TCGA-BRCA | Primary Solid tumor |
| TCGA-S3-A6ZG | TCGA-BRCA | Primary Solid tumor |
| TCGA-BH-A203 | TCGA-BRCA | Primary Solid tumor |
| TCGA-A8-A07S | TCGA-BRCA | Primary Solid tumor |
| TCGA-AC-A5EH | TCGA-BRCA | Primary Solid tumor |
| TCGA-A7-A13E | TCGA-BRCA | Primary Solid tumor |
| TCGA-E2-A1BC | TCGA-BRCA | Primary Solid tumor |
| TCGA-A8-A09G | TCGA-BRCA | Primary Solid tumor |
| TCGA-D8-A1JC | TCGA-BRCA | Primary Solid tumor |
| TCGA-A7-A5ZX | TCGA-BRCA | Primary Solid tumor |
| TCGA-GM-A3XL | TCGA-BRCA | Primary Solid tumor |
| TCGA-AC-A8OP | TCGA-BRCA | Primary Solid tumor |
| TCGA-AO-A12C | TCGA-BRCA | Primary Solid tumor |
| TCGA-AN-A0AK | TCGA-BRCA | Primary Solid tumor |
| TCGA-AO-A1KS | TCGA-BRCA | Primary Solid tumor |
| TCGA-E9-A3Q9 | TCGA-BRCA | Primary Solid tumor |
| TCGA-AR-A24M | TCGA-BRCA | Primary Solid tumor |
| TCGA-BH-A18G | TCGA-BRCA | Primary Solid tumor |
| TCGA-B6-A401 | TCGA-BRCA | Primary Solid tumor |
| TCGA-BH-A0DL | TCGA-BRCA | Primary Solid tumor |
| TCGA-B6-A0RT | TCGA-BRCA | Primary Solid tumor |
| TCGA-LL-A5YO | TCGA-BRCA | Primary Solid tumor |
| TCGA-AR-A1AU | TCGA-BRCA | Primary Solid tumor |
| TCGA-OL-A5RY | TCGA-BRCA | Primary Solid tumor |
| TCGA-GM-A3NY | TCGA-BRCA | Primary Solid tumor |
| TCGA-E9-A6HE | TCGA-BRCA | Primary Solid tumor |
| TCGA-BH-A0E9 | TCGA-BRCA | Primary Solid tumor |

| TCGA_patient_barcode | Project ID | Sample_type |
|---|---|---|
| TCGA-A2-A4S0 | TCGA-BRCA | Primary Solid tumor |
| TCGA-D8-A1XM | TCGA-BRCA | Primary Solid tumor |
| TCGA-A1-A0SK | TCGA-BRCA | Primary Solid tumor |
| TCGA-AN-A04A | TCGA-BRCA | Primary Solid tumor |
| TCGA-BH-A1F2 | TCGA-BRCA | Primary Solid tumor |
| TCGA-OL-A66P | TCGA-BRCA | Primary Solid tumor |
| TCGA-BH-A1ES | TCGA-BRCA | Primary Solid tumor |
| TCGA-D8-A3Z5 | TCGA-BRCA | Primary Solid tumor |
| TCGA-A2-A0CR | TCGA-BRCA | Primary Solid tumor |
| TCGA-D8-A27F | TCGA-BRCA | Primary Solid tumor |
| TCGA-A2-A1G0 | TCGA-BRCA | Primary Solid tumor |
| TCGA-EW-A1P3 | TCGA-BRCA | Primary Solid tumor |
| TCGA-A8-A07P | TCGA-BRCA | Primary Solid tumor |
| TCGA-LL-A440 | TCGA-BRCA | Primary Solid tumor |
| TCGA-C8-A1HL | TCGA-BRCA | Primary Solid tumor |
| TCGA-EW-A1OX | TCGA-BRCA | Primary Solid tumor |
| TCGA-GM-A3XN | TCGA-BRCA | Primary Solid tumor |
| TCGA-E9-A295 | TCGA-BRCA | Primary Solid tumor |
| TCGA-AO-A03L | TCGA-BRCA | Primary Solid tumor |
| TCGA-A2-A0T0 | TCGA-BRCA | Primary Solid tumor |
| TCGA-A7-A13F | TCGA-BRCA | Primary Solid tumor |
| TCGA-A8-A06P | TCGA-BRCA | Primary Solid tumor |
| TCGA-D8-A27K | TCGA-BRCA | Primary Solid tumor |
| TCGA-B6-A0I8 | TCGA-BRCA | Primary Solid tumor |
| TCGA-AC-A2FE | TCGA-BRCA | Primary Solid tumor |
| TCGA-OL-A6VO | TCGA-BRCA | Primary Solid tumor |
| TCGA-A2-A0D0 | TCGA-BRCA | Primary Solid tumor |
| TCGA-A8-A093 | TCGA-BRCA | Primary Solid tumor |
| TCGA-AO-A0JA | TCGA-BRCA | Primary Solid tumor |
| TCGA-AC-A2QI | TCGA-BRCA | Primary Solid tumor |
| TCGA-AO-A03V | TCGA-BRCA | Primary Solid tumor |
| TCGA-OL-A5D8 | TCGA-BRCA | Primary Solid tumor |
| TCGA-B6-A0RU | TCGA-BRCA | Primary Solid tumor |
| TCGA-GM-A2DO | TCGA-BRCA | Primary Solid tumor |
| TCGA-BH-A0HN | TCGA-BRCA | Primary Solid tumor |
| TCGA-AN-A0FJ | TCGA-BRCA | Primary Solid tumor |
| TCGA-AN-A0FF | TCGA-BRCA | Primary Solid tumor |
| TCGA-E2-A15D | TCGA-BRCA | Primary Solid tumor |
| TCGA-GM-A2DH | TCGA-BRCA | Primary Solid tumor |
| TCGA-BH-A0HL | TCGA-BRCA | Primary Solid tumor |
| TCGA-A8-A08T | TCGA-BRCA | Primary Solid tumor |
| TCGA-D8-A1JT | TCGA-BRCA | Primary Solid tumor |
| TCGA-EW-A1PG | TCGA-BRCA | Primary Solid tumor |
| TCGA-AO-A126 | TCGA-BRCA | Primary Solid tumor |
| TCGA-A7-A26E | TCGA-BRCA | Primary Solid tumor |
| TCGA-A2-A0D1 | TCGA-BRCA | Primary Solid tumor |

| TCGA_patient_barcode | Project ID | Sample_type |
|---|---|---|
| TCGA-C8-A131 | TCGA-BRCA | Primary Solid tumor |
| TCGA-C8-A26X | TCGA-BRCA | Primary Solid tumor |
| TCGA-BH-A18J | TCGA-BRCA | Primary Solid tumor |
| TCGA-C8-A132 | TCGA-BRCA | Primary Solid tumor |
| TCGA-A2-A0SY | TCGA-BRCA | Primary Solid tumor |
| TCGA-AQ-A04J | TCGA-BRCA | Primary Solid tumor |
| TCGA-C8-A12O | TCGA-BRCA | Primary Solid tumor |
| TCGA-B6-A1KC | TCGA-BRCA | Primary Solid tumor |
| TCGA-BH-A8G0 | TCGA-BRCA | Primary Solid tumor |
| TCGA-AR-A2LO | TCGA-BRCA | Primary Solid tumor |
| TCGA-AR-A252 | TCGA-BRCA | Primary Solid tumor |
| TCGA-D8-A73X | TCGA-BRCA | Primary Solid tumor |
| TCGA-B6-A0RH | TCGA-BRCA | Primary Solid tumor |
| TCGA-AC-A62V | TCGA-BRCA | Primary Solid tumor |
| TCGA-D8-A147 | TCGA-BRCA | Primary Solid tumor |
| TCGA-A2-A0CW | TCGA-BRCA | Primary Solid tumor |
| TCGA-A7-A426 | TCGA-BRCA | Primary Solid tumor |
| TCGA-AR-A2LK | TCGA-BRCA | Primary Solid tumor |
| TCGA-EW-A1IY | TCGA-BRCA | Primary Solid tumor |
| TCGA-EW-A2FV | TCGA-BRCA | Primary Solid tumor |
| TCGA-A7-A0DC | TCGA-BRCA | Primary Solid tumor |
| TCGA-E2-A3DX | TCGA-BRCA | Primary Solid tumor |
| TCGA-E9-A22D | TCGA-BRCA | Primary Solid tumor |
| TCGA-AC-A2FK | TCGA-BRCA | Primary Solid tumor |
| TCGA-BH-A0BG | TCGA-BRCA | Primary Solid tumor |
| TCGA-AQ-A54O | TCGA-BRCA | Primary Solid tumor |
| TCGA-A7-A13H | TCGA-BRCA | Primary Solid tumor |
| TCGA-AO-A0J9 | TCGA-BRCA | Primary Solid tumor |
| TCGA-E9-A1R5 | TCGA-BRCA | Primary Solid tumor |
| TCGA-A8-A09Z | TCGA-BRCA | Primary Solid tumor |
| TCGA-BH-A0W7 | TCGA-BRCA | Primary Solid tumor |
| TCGA-A2-A0T7 | TCGA-BRCA | Primary Solid tumor |
| TCGA-D8-A140 | TCGA-BRCA | Primary Solid tumor |
| TCGA-E2-A1L8 | TCGA-BRCA | Primary Solid tumor |
| TCGA-OL-A5S0 | TCGA-BRCA | Primary Solid tumor |
| TCGA-D8-A1Y3 | TCGA-BRCA | Primary Solid tumor |
| TCGA-BH-A0H9 | TCGA-BRCA | Primary Solid tumor |
| TCGA-LL-A7SZ | TCGA-BRCA | Primary Solid tumor |
| TCGA-A7-A3J0 | TCGA-BRCA | Primary Solid tumor |
| TCGA-EW-A1P5 | TCGA-BRCA | Primary Solid tumor |
| TCGA-BH-A18R | TCGA-BRCA | Primary Solid tumor |
| TCGA-GM-A2DI | TCGA-BRCA | Primary Solid tumor |
| TCGA-E9-A3X8 | TCGA-BRCA | Primary Solid tumor |
| TCGA-BH-A1EV | TCGA-BRCA | Primary Solid tumor |
| TCGA-BH-A18T | TCGA-BRCA | Primary Solid tumor |
| TCGA-BH-A0DH | TCGA-BRCA | Primary Solid tumor |

| TCGA_patient_barcode | Project ID | Sample_type |
|---|---|---|
| TCGA-D8-A1XZ | TCGA-BRCA | Primary Solid tumor |
| TCGA-BH-A8FY | TCGA-BRCA | Primary Solid tumor |
| TCGA-C8-A26Z | TCGA-BRCA | Primary Solid tumor |
| TCGA-D8-A3Z6 | TCGA-BRCA | Primary Solid tumor |
| TCGA-LL-A442 | TCGA-BRCA | Primary Solid tumor |
| TCGA-BH-A0H5 | TCGA-BRCA | Primary Solid tumor |
| TCGA-D8-A27L | TCGA-BRCA | Primary Solid tumor |
| TCGA-AC-A6IW | TCGA-BRCA | Primary Solid tumor |
| TCGA-AC-A23G | TCGA-BRCA | Primary Solid tumor |
| TCGA-D8-A1JJ | TCGA-BRCA | Primary Solid tumor |
| TCGA-A8-A084 | TCGA-BRCA | Primary Solid tumor |
| TCGA-LL-A6FQ | TCGA-BRCA | Primary Solid tumor |
| TCGA-AR-A24K | TCGA-BRCA | Primary Solid tumor |
| TCGA-AO-A12H | TCGA-BRCA | Primary Solid tumor |
| TCGA-E2-A14N | TCGA-BRCA | Primary Solid tumor |
| TCGA-E9-A1N5 | TCGA-BRCA | Primary Solid tumor |
| TCGA-B6-A0RS | TCGA-BRCA | Primary Solid tumor |
| TCGA-BH-A0BV | TCGA-BRCA | Primary Solid tumor |
| TCGA-EW-A2FW | TCGA-BRCA | Primary Solid tumor |
| TCGA-B6-A40B | TCGA-BRCA | Primary Solid tumor |
| TCGA-A8-A07J | TCGA-BRCA | Primary Solid tumor |
| TCGA-AC-A3HN | TCGA-BRCA | Primary Solid tumor |
| TCGA-BH-A18N | TCGA-BRCA | Primary Solid tumor |
| TCGA-AR-A0TS | TCGA-BRCA | Primary Solid tumor |
| TCGA-D8-A1JE | TCGA-BRCA | Primary Solid tumor |
| TCGA-C8-A130 | TCGA-BRCA | Primary Solid tumor |
| TCGA-LL-A9Q3 | TCGA-BRCA | Primary Solid tumor |
| TCGA-A2-A3XZ | TCGA-BRCA | Primary Solid tumor |
| TCGA-D8-A1XL | TCGA-BRCA | Primary Solid tumor |
| TCGA-AN-A0AR | TCGA-BRCA | Primary Solid tumor |
| TCGA-BH-A18U | TCGA-BRCA | Primary Solid tumor |
| TCGA-B6-A0WW | TCGA-BRCA | Primary Solid tumor |
| TCGA-A7-A0D9 | TCGA-BRCA | Primary Solid tumor |
| TCGA-A2-A25E | TCGA-BRCA | Primary Solid tumor |
| TCGA-AC-A2FM | TCGA-BRCA | Primary Solid tumor |
| TCGA-JL-A3YW | TCGA-BRCA | Primary Solid tumor |
| TCGA-PL-A8LY | TCGA-BRCA | Primary Solid tumor |
| TCGA-B6-A0RN | TCGA-BRCA | Primary Solid tumor |
| TCGA-A2-A0EO | TCGA-BRCA | Primary Solid tumor |
| TCGA-AQ-A1H2 | TCGA-BRCA | Primary Solid tumor |
| TCGA-EW-A1OZ | TCGA-BRCA | Primary Solid tumor |
| TCGA-OL-A66I | TCGA-BRCA | Primary Solid tumor |
| TCGA-BH-A0B4 | TCGA-BRCA | Primary Solid tumor |
| TCGA-BH-A0BQ | TCGA-BRCA | Primary Solid tumor |
| TCGA-C8-A1HN | TCGA-BRCA | Primary Solid tumor |
| TCGA-BH-A0W3 | TCGA-BRCA | Primary Solid tumor |

| TCGA_patient_barcode | Project ID | Sample_type |
|---|---|---|
| TCGA-AN-A0FY | TCGA-BRCA | Primary Solid tumor |
| TCGA-E2-A15H | TCGA-BRCA | Primary Solid tumor |
| TCGA-E2-A14U | TCGA-BRCA | Primary Solid tumor |
| TCGA-E2-A14Z | TCGA-BRCA | Primary Solid tumor |
| TCGA-B6-A1KN | TCGA-BRCA | Primary Solid tumor |
| TCGA-BH-A0AU | TCGA-BRCA | Primary Solid tumor |
| TCGA-E2-A15J | TCGA-BRCA | Primary Solid tumor |
| TCGA-E2-A1LK | TCGA-BRCA | Primary Solid tumor |
| TCGA-A8-A092 | TCGA-BRCA | Primary Solid tumor |
| TCGA-A8-A0A2 | TCGA-BRCA | Primary Solid tumor |
| TCGA-A7-A26F | TCGA-BRCA | Primary Solid tumor |
| TCGA-BH-A18F | TCGA-BRCA | Primary Solid tumor |
| TCGA-BH-A0B3 | TCGA-BRCA | Primary Solid tumor |
| TCGA-E2-A10A | TCGA-BRCA | Primary Solid tumor |
| TCGA-BH-A1FB | TCGA-BRCA | Primary Solid tumor |
| TCGA-GM-A2DD | TCGA-BRCA | Primary Solid tumor |
| TCGA-LQ-A4E4 | TCGA-BRCA | Primary Solid tumor |
| TCGA-D8-A1XW | TCGA-BRCA | Primary Solid tumor |
| TCGA-E9-A3QA | TCGA-BRCA | Primary Solid tumor |
| TCGA-E9-A1RE | TCGA-BRCA | Primary Solid tumor |
| TCGA-E2-A1IK | TCGA-BRCA | Primary Solid tumor |
| TCGA-E2-A1IU | TCGA-BRCA | Primary Solid tumor |
| TCGA-A2-A259 | TCGA-BRCA | Primary Solid tumor |
| TCGA-AC-A2QJ | TCGA-BRCA | Primary Solid tumor |
| TCGA-AN-A0XP | TCGA-BRCA | Primary Solid tumor |
| TCGA-AC-A2FG | TCGA-BRCA | Primary Solid tumor |
| TCGA-BH-A42U | TCGA-BRCA | Primary Solid tumor |
| TCGA-D8-A4Z1 | TCGA-BRCA | Primary Solid tumor |
| TCGA-EW-A1J3 | TCGA-BRCA | Primary Solid tumor |
| TCGA-LD-A66U | TCGA-BRCA | Primary Solid tumor |
| TCGA-E2-A1LA | TCGA-BRCA | Primary Solid tumor |
| TCGA-E2-A1LG | TCGA-BRCA | Primary Solid tumor |
| TCGA-BH-A1F5 | TCGA-BRCA | Primary Solid tumor |
| TCGA-C8-A26V | TCGA-BRCA | Primary Solid tumor |
| TCGA-E9-A1R2 | TCGA-BRCA | Primary Solid tumor |
| TCGA-AC-A5XU | TCGA-BRCA | Primary Solid tumor |
| TCGA-A7-A5ZW | TCGA-BRCA | Primary Solid tumor |
| TCGA-BH-A0E7 | TCGA-BRCA | Primary Solid tumor |
| TCGA-A7-A26J | TCGA-BRCA | Primary Solid tumor |
| TCGA-BH-A0H3 | TCGA-BRCA | Primary Solid tumor |
| TCGA-BH-A0DX | TCGA-BRCA | Primary Solid tumor |
| TCGA-D8-A1XG | TCGA-BRCA | Primary Solid tumor |
| TCGA-A7-A0CD | TCGA-BRCA | Primary Solid tumor |
| TCGA-A2-A0T3 | TCGA-BRCA | Primary Solid tumor |
| TCGA-E9-A1R7 | TCGA-BRCA | Primary Solid tumor |
| TCGA-BH-A0B6 | TCGA-BRCA | Primary Solid tumor |

| **TCGA_patient_barcode** | **Project ID** | **Sample_type** |
|---|---|---|
| TCGA-A2-A0CV | TCGA-BRCA | Primary Solid tumor |
| TCGA-E2-A2P6 | TCGA-BRCA | Primary Solid tumor |
| TCGA-AN-A0FS | TCGA-BRCA | Primary Solid tumor |
| TCGA-A7-A3IZ | TCGA-BRCA | Primary Solid tumor |
| TCGA-C8-A1HE | TCGA-BRCA | Primary Solid tumor |
| TCGA-D8-A27T | TCGA-BRCA | Primary Solid tumor |
| TCGA-A8-A09D | TCGA-BRCA | Primary Solid tumor |
| TCGA-BH-A18L | TCGA-BRCA | Primary Solid tumor |
| TCGA-A2-A4S2 | TCGA-BRCA | Primary Solid tumor |
| TCGA-OL-A5DA | TCGA-BRCA | Primary Solid tumor |
| TCGA-AR-A0TQ | TCGA-BRCA | Primary Solid tumor |
| TCGA-B6-A0IG | TCGA-BRCA | Primary Solid tumor |
| TCGA-W8-A86G | TCGA-BRCA | Primary Solid tumor |
| TCGA-OL-A5D7 | TCGA-BRCA | Primary Solid tumor |
| TCGA-A7-A0DB | TCGA-BRCA | Primary Solid tumor |
| TCGA-D8-A27P | TCGA-BRCA | Primary Solid tumor |
| TCGA-AR-A1AI | TCGA-BRCA | Primary Solid tumor |
| TCGA-E2-A1IF | TCGA-BRCA | Primary Solid tumor |
| TCGA-E2-A1IL | TCGA-BRCA | Primary Solid tumor |
| TCGA-A2-A0EN | TCGA-BRCA | Primary Solid tumor |
| TCGA-D8-A13Y | TCGA-BRCA | Primary Solid tumor |
| TCGA-BH-A0W4 | TCGA-BRCA | Primary Solid tumor |
| TCGA-E2-A1LL | TCGA-BRCA | Primary Solid tumor |
| TCGA-B6-A0RO | TCGA-BRCA | Primary Solid tumor |
| TCGA-A8-A085 | TCGA-BRCA | Primary Solid tumor |
| TCGA-E2-A14Q | TCGA-BRCA | Primary Solid tumor |
| TCGA-AC-A2FO | TCGA-BRCA | Primary Solid tumor |
| TCGA-BH-A0DK | TCGA-BRCA | Primary Solid tumor |
| TCGA-A8-A082 | TCGA-BRCA | Primary Solid tumor |
| TCGA-E2-A14X | TCGA-BRCA | Primary Solid tumor |
| TCGA-AR-A5QN | TCGA-BRCA | Primary Solid tumor |
| TCGA-C8-A274 | TCGA-BRCA | Primary Solid tumor |
| TCGA-A2-A3Y0 | TCGA-BRCA | Primary Solid tumor |
| TCGA-BH-A0HI | TCGA-BRCA | Primary Solid tumor |
| TCGA-A2-A0EY | TCGA-BRCA | Primary Solid tumor |
| TCGA-E9-A24A | TCGA-BRCA | Primary Solid tumor |
| TCGA-B6-A0WY | TCGA-BRCA | Primary Solid tumor |
| TCGA-D8-A1XQ | TCGA-BRCA | Primary Solid tumor |
| TCGA-C8-A12W | TCGA-BRCA | Primary Solid tumor |
| TCGA-D8-A1JB | TCGA-BRCA | Primary Solid tumor |
| TCGA-A2-A0T5 | TCGA-BRCA | Primary Solid tumor |
| TCGA-LL-A6FP | TCGA-BRCA | Primary Solid tumor |
| TCGA-BH-A0B9 | TCGA-BRCA | Primary Solid tumor |
| TCGA-A7-A0CH | TCGA-BRCA | Primary Solid tumor |
| TCGA-E9-A22G | TCGA-BRCA | Primary Solid tumor |
| TCGA-D8-A13Z | TCGA-BRCA | Primary Solid tumor |

| TCGA_patient_barcode | Project ID | Sample_type |
|---|---|---|
| TCGA-A8-A06Z | TCGA-BRCA | Primary Solid tumor |
| TCGA-A2-A1G4 | TCGA-BRCA | Primary Solid tumor |
| TCGA-PE-A5DD | TCGA-BRCA | Primary Solid tumor |
| TCGA-AC-A2FB | TCGA-BRCA | Primary Solid tumor |
| TCGA-C8-A1HK | TCGA-BRCA | Primary Solid tumor |
| TCGA-AN-A0XL | TCGA-BRCA | Primary Solid tumor |
| TCGA-A1-A0SO | TCGA-BRCA | Primary Solid tumor |
| TCGA-AR-A0U4 | TCGA-BRCA | Primary Solid tumor |
| TCGA-AR-A1AM | TCGA-BRCA | Primary Solid tumor |
| TCGA-E2-A15A | TCGA-BRCA | Primary Solid tumor |
| TCGA-E2-A15P | TCGA-BRCA | Primary Solid tumor |
| TCGA-A2-A04Q | TCGA-BRCA | Primary Solid tumor |
| TCGA-D8-A1JH | TCGA-BRCA | Primary Solid tumor |
| TCGA-A2-A0EW | TCGA-BRCA | Primary Solid tumor |
| TCGA-BH-A18M | TCGA-BRCA | Primary Solid tumor |
| TCGA-A2-A0YI | TCGA-BRCA | Primary Solid tumor |
| TCGA-AO-A0J4 | TCGA-BRCA | Primary Solid tumor |
| TCGA-E2-A15F | TCGA-BRCA | Primary Solid tumor |
| TCGA-A2-A1FV | TCGA-BRCA | Primary Solid tumor |
| TCGA-A7-A0CJ | TCGA-BRCA | Primary Solid tumor |
| TCGA-AC-A8OR | TCGA-BRCA | Primary Solid tumor |
| TCGA-A2-A0YJ | TCGA-BRCA | Primary Solid tumor |
| TCGA-B6-A0I6 | TCGA-BRCA | Primary Solid tumor |
| TCGA-A8-A097 | TCGA-BRCA | Primary Solid tumor |
| TCGA-B6-A0RQ | TCGA-BRCA | Primary Solid tumor |
| TCGA-AR-A1AK | TCGA-BRCA | Primary Solid tumor |
| TCGA-BH-A0DD | TCGA-BRCA | Primary Solid tumor |
| TCGA-E9-A245 | TCGA-BRCA | Primary Solid tumor |
| TCGA-GI-A2C8 | TCGA-BRCA | Primary Solid tumor |
| TCGA-BH-A6R9 | TCGA-BRCA | Primary Solid tumor |
| TCGA-AC-A2BM | TCGA-BRCA | Primary Solid tumor |
| TCGA-A8-A09Q | TCGA-BRCA | Primary Solid tumor |
| TCGA-LL-A73Z | TCGA-BRCA | Primary Solid tumor |
| TCGA-AN-A0FN | TCGA-BRCA | Primary Solid tumor |
| TCGA-BH-A1EY | TCGA-BRCA | Primary Solid tumor |
| TCGA-E9-A226 | TCGA-BRCA | Primary Solid tumor |
| TCGA-AR-A0U0 | TCGA-BRCA | Primary Solid tumor |
| TCGA-AO-A129 | TCGA-BRCA | Primary Solid tumor |
| TCGA-C8-A26Y | TCGA-BRCA | Primary Solid tumor |
| TCGA-AR-A2LQ | TCGA-BRCA | Primary Solid tumor |
| TCGA-D8-A1XD | TCGA-BRCA | Primary Solid tumor |
| TCGA-E2-A570 | TCGA-BRCA | Primary Solid tumor |
| TCGA-E2-A574 | TCGA-BRCA | Primary Solid tumor |
| TCGA-D8-A73W | TCGA-BRCA | Primary Solid tumor |
| TCGA-B6-A2IU | TCGA-BRCA | Primary Solid tumor |
| TCGA-5X-AA5U | TCGA-OV | Primary Solid tumor |

| TCGA_patient_barcode | Project ID | Sample_type |
|---|---|---|
| TCGA-25-2400 | TCGA-OV | Primary Solid tumor |
| TCGA-24-2290 | TCGA-OV | Primary Solid tumor |
| TCGA-09-2054 | TCGA-OV | Primary Solid tumor |
| TCGA-13-1489 | TCGA-OV | Primary Solid tumor |
| TCGA-61-2088 | TCGA-OV | Primary Solid tumor |
| TCGA-24-1923 | TCGA-OV | Primary Solid tumor |
| TCGA-13-0726 | TCGA-OV | Primary Solid tumor |
| TCGA-23-1022 | TCGA-OV | Primary Solid tumor |
| TCGA-31-1956 | TCGA-OV | Primary Solid tumor |
| TCGA-13-0800 | TCGA-OV | Primary Solid tumor |
| TCGA-25-1326 | TCGA-OV | Primary Solid tumor |
| TCGA-13-1403 | TCGA-OV | Primary Solid tumor |
| TCGA-25-1322 | TCGA-OV | Primary Solid tumor |
| TCGA-24-1558 | TCGA-OV | Primary Solid tumor |
| TCGA-24-2288 | TCGA-OV | Primary Solid tumor |
| TCGA-25-1626 | TCGA-OV | Primary Solid tumor |
| TCGA-24-2035 | TCGA-OV | Primary Solid tumor |
| TCGA-57-1584 | TCGA-OV | Primary Solid tumor |
| TCGA-61-2012 | TCGA-OV | Primary Solid tumor |
| TCGA-24-1847 | TCGA-OV | Primary Solid tumor |
| TCGA-61-2111 | TCGA-OV | Primary Solid tumor |
| TCGA-20-0987 | TCGA-OV | Primary Solid tumor |
| TCGA-09-2056 | TCGA-OV | Primary Solid tumor |
| TCGA-24-1546 | TCGA-OV | Primary Solid tumor |
| TCGA-30-1891 | TCGA-OV | Primary Solid tumor |
| TCGA-09-1662 | TCGA-OV | Primary Solid tumor |
| TCGA-31-1951 | TCGA-OV | Primary Solid tumor |
| TCGA-24-1427 | TCGA-OV | Primary Solid tumor |
| TCGA-20-1687 | TCGA-OV | Primary Solid tumor |
| TCGA-13-1483 | TCGA-OV | Primary Solid tumor |
| TCGA-29-1705 | TCGA-OV | Primary Solid tumor |
| TCGA-24-2024 | TCGA-OV | Primary Solid tumor |
| TCGA-24-1843 | TCGA-OV | Primary Solid tumor |
| TCGA-23-1122 | TCGA-OV | Primary Solid tumor |
| TCGA-13-0886 | TCGA-OV | Primary Solid tumor |
| TCGA-24-1464 | TCGA-OV | Primary Solid tumor |
| TCGA-13-0906 | TCGA-OV | Primary Solid tumor |
| TCGA-24-2254 | TCGA-OV | Primary Solid tumor |
| TCGA-29-2414 | TCGA-OV | Primary Solid tumor |
| TCGA-13-1487 | TCGA-OV | Primary Solid tumor |
| TCGA-24-2281 | TCGA-OV | Primary Solid tumor |
| TCGA-24-1422 | TCGA-OV | Primary Solid tumor |
| TCGA-30-1853 | TCGA-OV | Primary Solid tumor |
| TCGA-25-1627 | TCGA-OV | Primary Solid tumor |
| TCGA-25-1635 | TCGA-OV | Primary Solid tumor |
| TCGA-24-2297 | TCGA-OV | Primary Solid tumor |

| TCGA_patient_barcode | Project ID | Sample_type |
|---|---|---|
| TCGA-04-1356 | TCGA-OV | Primary Solid tumor |
| TCGA-13-0891 | TCGA-OV | Primary Solid tumor |
| TCGA-25-2409 | TCGA-OV | Primary Solid tumor |
| TCGA-61-2008 | TCGA-OV | Primary Solid tumor |
| TCGA-59-2348 | TCGA-OV | Primary Solid tumor |
| TCGA-09-1673 | TCGA-OV | Primary Solid tumor |
| TCGA-24-1418 | TCGA-OV | Primary Solid tumor |
| TCGA-24-0970 | TCGA-OV | Primary Solid tumor |
| TCGA-09-2048 | TCGA-OV | Primary Solid tumor |
| TCGA-24-1845 | TCGA-OV | Primary Solid tumor |
| TCGA-23-1119 | TCGA-OV | Primary Solid tumor |
| TCGA-36-1570 | TCGA-OV | Primary Solid tumor |
| TCGA-24-0966 | TCGA-OV | Primary Solid tumor |
| TCGA-57-1994 | TCGA-OV | Primary Solid tumor |
| TCGA-09-1667 | TCGA-OV | Primary Solid tumor |
| TCGA-20-1683 | TCGA-OV | Primary Solid tumor |
| TCGA-13-1507 | TCGA-OV | Primary Solid tumor |
| TCGA-24-2033 | TCGA-OV | Primary Solid tumor |
| TCGA-24-1553 | TCGA-OV | Primary Solid tumor |
| TCGA-24-2271 | TCGA-OV | Primary Solid tumor |
| TCGA-36-1568 | TCGA-OV | Primary Solid tumor |
| TCGA-61-1737 | TCGA-OV | Primary Solid tumor |
| TCGA-24-2262 | TCGA-OV | Primary Solid tumor |
| TCGA-09-1669 | TCGA-OV | Primary Solid tumor |
| TCGA-24-2020 | TCGA-OV | Primary Solid tumor |
| TCGA-25-1870 | TCGA-OV | Primary Solid tumor |
| TCGA-61-1741 | TCGA-OV | Primary Solid tumor |
| TCGA-61-1911 | TCGA-OV | Primary Solid tumor |
| TCGA-13-0730 | TCGA-OV | Primary Solid tumor |
| TCGA-25-1328 | TCGA-OV | Primary Solid tumor |
| TCGA-24-2293 | TCGA-OV | Primary Solid tumor |
| TCGA-13-2060 | TCGA-OV | Primary Solid tumor |
| TCGA-24-1565 | TCGA-OV | Primary Solid tumor |
| TCGA-13-1495 | TCGA-OV | Primary Solid tumor |
| TCGA-23-2084 | TCGA-OV | Primary Solid tumor |
| TCGA-24-1435 | TCGA-OV | Primary Solid tumor |
| TCGA-23-1116 | TCGA-OV | Primary Solid tumor |
| TCGA-25-1315 | TCGA-OV | Primary Solid tumor |
| TCGA-36-1581 | TCGA-OV | Primary Solid tumor |
| TCGA-10-0931 | TCGA-OV | Primary Solid tumor |
| TCGA-24-1563 | TCGA-OV | Primary Solid tumor |
| TCGA-25-1320 | TCGA-OV | Primary Solid tumor |
| TCGA-13-0900 | TCGA-OV | Primary Solid tumor |
| TCGA-24-2027 | TCGA-OV | Primary Solid tumor |
| TCGA-61-1725 | TCGA-OV | Primary Solid tumor |
| TCGA-04-1343 | TCGA-OV | Primary Solid tumor |

| TCGA_patient_barcode | Project ID | Sample_type |
|---|---|---|
| TCGA-61-1998 | TCGA-OV | Primary Solid tumor |
| TCGA-23-1123 | TCGA-OV | Primary Solid tumor |
| TCGA-61-1995 | TCGA-OV | Primary Solid tumor |
| TCGA-13-1407 | TCGA-OV | Primary Solid tumor |
| TCGA-13-1510 | TCGA-OV | Primary Solid tumor |
| TCGA-13-1505 | TCGA-OV | Primary Solid tumor |
| TCGA-25-1317 | TCGA-OV | Primary Solid tumor |
| TCGA-61-1910 | TCGA-OV | Primary Solid tumor |
| TCGA-04-1331 | TCGA-OV | Primary Solid tumor |
| TCGA-25-1329 | TCGA-OV | Primary Solid tumor |
| TCGA-29-1781 | TCGA-OV | Primary Solid tumor |
| TCGA-25-1634 | TCGA-OV | Primary Solid tumor |
| TCGA-29-1777 | TCGA-OV | Primary Solid tumor |
| TCGA-09-2053 | TCGA-OV | Primary Solid tumor |
| TCGA-24-1567 | TCGA-OV | Primary Solid tumor |
| TCGA-WR-A838 | TCGA-OV | Primary Solid tumor |
| TCGA-13-A5FT | TCGA-OV | Primary Solid tumor |
| TCGA-61-1724 | TCGA-OV | Primary Solid tumor |
| TCGA-29-2425 | TCGA-OV | Primary Solid tumor |
| TCGA-61-2109 | TCGA-OV | Primary Solid tumor |
| TCGA-04-1332 | TCGA-OV | Primary Solid tumor |
| TCGA-04-1648 | TCGA-OV | Primary Solid tumor |
| TCGA-13-0923 | TCGA-OV | Primary Solid tumor |
| TCGA-04-1347 | TCGA-OV | Primary Solid tumor |
| TCGA-13-0727 | TCGA-OV | Primary Solid tumor |
| TCGA-24-1471 | TCGA-OV | Primary Solid tumor |
| TCGA-20-0991 | TCGA-OV | Primary Solid tumor |
| TCGA-24-1844 | TCGA-OV | Primary Solid tumor |
| TCGA-61-2097 | TCGA-OV | Primary Solid tumor |
| TCGA-36-1574 | TCGA-OV | Primary Solid tumor |
| TCGA-25-2391 | TCGA-OV | Primary Solid tumor |
| TCGA-59-2363 | TCGA-OV | Primary Solid tumor |
| TCGA-36-1576 | TCGA-OV | Primary Solid tumor |
| TCGA-10-0927 | TCGA-OV | Primary Solid tumor |
| TCGA-29-1690 | TCGA-OV | Primary Solid tumor |
| TCGA-25-1313 | TCGA-OV | Primary Solid tumor |
| TCGA-29-2427 | TCGA-OV | Primary Solid tumor |
| TCGA-09-0367 | TCGA-OV | Primary Solid tumor |
| TCGA-13-0885 | TCGA-OV | Primary Solid tumor |
| TCGA-30-1860 | TCGA-OV | Primary Solid tumor |
| TCGA-29-1697 | TCGA-OV | Primary Solid tumor |
| TCGA-23-1109 | TCGA-OV | Primary Solid tumor |
| TCGA-30-1866 | TCGA-OV | Primary Solid tumor |
| TCGA-24-2267 | TCGA-OV | Primary Solid tumor |
| TCGA-24-1103 | TCGA-OV | Primary Solid tumor |
| TCGA-04-1365 | TCGA-OV | Primary Solid tumor |

| **TCGA_patient_barcode** | **Project ID** | **Sample_type** |
|---|---|---|
| TCGA-13-1512 | TCGA-OV | Primary Solid tumor |
| TCGA-24-1423 | TCGA-OV | Primary Solid tumor |
| TCGA-61-2009 | TCGA-OV | Primary Solid tumor |
| TCGA-13-0883 | TCGA-OV | Primary Solid tumor |
| TCGA-24-1603 | TCGA-OV | Primary Solid tumor |
| TCGA-24-1430 | TCGA-OV | Primary Solid tumor |
| TCGA-10-0928 | TCGA-OV | Primary Solid tumor |
| TCGA-29-1688 | TCGA-OV | Primary Solid tumor |
| TCGA-24-2280 | TCGA-OV | Primary Solid tumor |
| TCGA-25-2401 | TCGA-OV | Primary Solid tumor |
| TCGA-29-1703 | TCGA-OV | Primary Solid tumor |
| TCGA-59-A5PD | TCGA-OV | Primary Solid tumor |
| TCGA-25-1633 | TCGA-OV | Primary Solid tumor |
| TCGA-13-1492 | TCGA-OV | Primary Solid tumor |
| TCGA-10-0937 | TCGA-OV | Primary Solid tumor |
| TCGA-23-1021 | TCGA-OV | Primary Solid tumor |
| TCGA-10-0938 | TCGA-OV | Primary Solid tumor |
| TCGA-04-1364 | TCGA-OV | Primary Solid tumor |
| TCGA-31-1946 | TCGA-OV | Primary Solid tumor |
| TCGA-04-1514 | TCGA-OV | Primary Solid tumor |
| TCGA-29-1784 | TCGA-OV | Primary Solid tumor |
| TCGA-23-1118 | TCGA-OV | Primary Solid tumor |
| TCGA-04-1341 | TCGA-OV | Primary Solid tumor |
| TCGA-13-1404 | TCGA-OV | Primary Solid tumor |
| TCGA-30-1857 | TCGA-OV | Primary Solid tumor |
| TCGA-25-1323 | TCGA-OV | Primary Solid tumor |
| TCGA-24-1549 | TCGA-OV | Primary Solid tumor |
| TCGA-61-1914 | TCGA-OV | Primary Solid tumor |
| TCGA-24-2298 | TCGA-OV | Primary Solid tumor |
| TCGA-61-2113 | TCGA-OV | Primary Solid tumor |
| TCGA-61-1918 | TCGA-OV | Primary Solid tumor |
| TCGA-24-1544 | TCGA-OV | Primary Solid tumor |
| TCGA-13-1509 | TCGA-OV | Primary Solid tumor |
| TCGA-25-2042 | TCGA-OV | Primary Solid tumor |
| TCGA-13-1405 | TCGA-OV | Primary Solid tumor |
| TCGA-13-0901 | TCGA-OV | Primary Solid tumor |
| TCGA-36-1571 | TCGA-OV | Primary Solid tumor |
| TCGA-36-1580 | TCGA-OV | Primary Solid tumor |
| TCGA-24-1104 | TCGA-OV | Primary Solid tumor |
| TCGA-13-1408 | TCGA-OV | Primary Solid tumor |
| TCGA-31-1950 | TCGA-OV | Primary Solid tumor |
| TCGA-09-1670 | TCGA-OV | Primary Solid tumor |
| TCGA-29-1776 | TCGA-OV | Primary Solid tumor |
| TCGA-13-0887 | TCGA-OV | Primary Solid tumor |
| TCGA-13-0725 | TCGA-OV | Primary Solid tumor |
| TCGA-09-1668 | TCGA-OV | Primary Solid tumor |

| TCGA_patient_barcode | Project ID | Sample_type |
|---|---|---|
| TCGA-24-1552 | TCGA-OV | Primary Solid tumor |
| TCGA-13-0768 | TCGA-OV | Primary Solid tumor |
| TCGA-13-0924 | TCGA-OV | Primary Solid tumor |
| TCGA-24-2026 | TCGA-OV | Primary Solid tumor |
| TCGA-13-0893 | TCGA-OV | Primary Solid tumor |
| TCGA-04-1362 | TCGA-OV | Primary Solid tumor |
| TCGA-13-0897 | TCGA-OV | Primary Solid tumor |
| TCGA-25-2399 | TCGA-OV | Primary Solid tumor |
| TCGA-24-1470 | TCGA-OV | Primary Solid tumor |
| TCGA-10-0933 | TCGA-OV | Primary Solid tumor |
| TCGA-24-2289 | TCGA-OV | Primary Solid tumor |
| TCGA-24-1557 | TCGA-OV | Primary Solid tumor |
| TCGA-23-1026 | TCGA-OV | Primary Solid tumor |
| TCGA-29-1701 | TCGA-OV | Primary Solid tumor |
| TCGA-29-1768 | TCGA-OV | Primary Solid tumor |
| TCGA-29-1693 | TCGA-OV | Primary Solid tumor |
| TCGA-04-1519 | TCGA-OV | Primary Solid tumor |
| TCGA-04-1655 | TCGA-OV | Primary Solid tumor |
| TCGA-59-2354 | TCGA-OV | Primary Solid tumor |
| TCGA-29-1763 | TCGA-OV | Primary Solid tumor |
| TCGA-20-1686 | TCGA-OV | Primary Solid tumor |
| TCGA-24-1431 | TCGA-OV | Primary Solid tumor |
| TCGA-24-1924 | TCGA-OV | Primary Solid tumor |
| TCGA-13-1409 | TCGA-OV | Primary Solid tumor |
| TCGA-04-1361 | TCGA-OV | Primary Solid tumor |
| TCGA-25-1319 | TCGA-OV | Primary Solid tumor |
| TCGA-13-1410 | TCGA-OV | Primary Solid tumor |
| TCGA-09-2044 | TCGA-OV | Primary Solid tumor |
| TCGA-24-1842 | TCGA-OV | Primary Solid tumor |
| TCGA-25-1628 | TCGA-OV | Primary Solid tumor |
| TCGA-09-1666 | TCGA-OV | Primary Solid tumor |
| TCGA-04-1338 | TCGA-OV | Primary Solid tumor |
| TCGA-13-0797 | TCGA-OV | Primary Solid tumor |
| TCGA-24-0979 | TCGA-OV | Primary Solid tumor |
| TCGA-25-2393 | TCGA-OV | Primary Solid tumor |
| TCGA-29-A5NZ | TCGA-OV | Primary Solid tumor |
| TCGA-OY-A56Q | TCGA-OV | Primary Solid tumor |
| TCGA-23-1027 | TCGA-OV | Primary Solid tumor |
| TCGA-04-1536 | TCGA-OV | Primary Solid tumor |
| TCGA-61-2110 | TCGA-OV | Primary Solid tumor |
| TCGA-23-1028 | TCGA-OV | Primary Solid tumor |
| TCGA-61-2002 | TCGA-OV | Primary Solid tumor |
| TCGA-13-0888 | TCGA-OV | Primary Solid tumor |
| TCGA-61-1721 | TCGA-OV | Primary Solid tumor |
| TCGA-29-1778 | TCGA-OV | Primary Solid tumor |
| TCGA-29-1785 | TCGA-OV | Primary Solid tumor |

| TCGA_patient_barcode | Project ID | Sample_type |
| --- | --- | --- |
| TCGA-30-1892 | TCGA-OV | Primary Solid tumor |
| TCGA-09-0366 | TCGA-OV | Primary Solid tumor |
| TCGA-VG-A8LO | TCGA-OV | Primary Solid tumor |
| TCGA-13-0762 | TCGA-OV | Primary Solid tumor |
| TCGA-24-1434 | TCGA-OV | Primary Solid tumor |
| TCGA-24-1416 | TCGA-OV | Primary Solid tumor |
| TCGA-57-1582 | TCGA-OV | Primary Solid tumor |
| TCGA-25-1630 | TCGA-OV | Primary Solid tumor |
| TCGA-25-1312 | TCGA-OV | Primary Solid tumor |
| TCGA-61-2000 | TCGA-OV | Primary Solid tumor |
| TCGA-61-2092 | TCGA-OV | Primary Solid tumor |
| TCGA-61-1907 | TCGA-OV | Primary Solid tumor |
| TCGA-25-1316 | TCGA-OV | Primary Solid tumor |
| TCGA-04-1530 | TCGA-OV | Primary Solid tumor |
| TCGA-23-1107 | TCGA-OV | Primary Solid tumor |
| TCGA-04-1542 | TCGA-OV | Primary Solid tumor |
| TCGA-23-1110 | TCGA-OV | Primary Solid tumor |
| TCGA-57-1585 | TCGA-OV | Primary Solid tumor |
| TCGA-13-0720 | TCGA-OV | Primary Solid tumor |
| TCGA-24-1551 | TCGA-OV | Primary Solid tumor |
| TCGA-24-1850 | TCGA-OV | Primary Solid tumor |
| TCGA-09-0364 | TCGA-OV | Primary Solid tumor |
| TCGA-29-2428 | TCGA-OV | Primary Solid tumor |
| TCGA-04-1350 | TCGA-OV | Primary Solid tumor |
| TCGA-61-1733 | TCGA-OV | Primary Solid tumor |
| TCGA-24-1562 | TCGA-OV | Primary Solid tumor |
| TCGA-09-2051 | TCGA-OV | Primary Solid tumor |
| TCGA-59-2352 | TCGA-OV | Primary Solid tumor |
| TCGA-29-1774 | TCGA-OV | Primary Solid tumor |
| TCGA-29-1694 | TCGA-OV | Primary Solid tumor |
| TCGA-57-1586 | TCGA-OV | Primary Solid tumor |
| TCGA-61-2104 | TCGA-OV | Primary Solid tumor |
| TCGA-04-1651 | TCGA-OV | Primary Solid tumor |
| TCGA-24-1417 | TCGA-OV | Primary Solid tumor |
| TCGA-23-2078 | TCGA-OV | Primary Solid tumor |
| TCGA-24-1413 | TCGA-OV | Primary Solid tumor |
| TCGA-31-1959 | TCGA-OV | Primary Solid tumor |
| TCGA-30-1861 | TCGA-OV | Primary Solid tumor |
| TCGA-61-1900 | TCGA-OV | Primary Solid tumor |
| TCGA-24-1604 | TCGA-OV | Primary Solid tumor |
| TCGA-25-2398 | TCGA-OV | Primary Solid tumor |
| TCGA-25-2404 | TCGA-OV | Primary Solid tumor |
| TCGA-29-1783 | TCGA-OV | Primary Solid tumor |
| TCGA-13-1485 | TCGA-OV | Primary Solid tumor |
| TCGA-36-1569 | TCGA-OV | Primary Solid tumor |
| TCGA-24-1846 | TCGA-OV | Primary Solid tumor |

| **TCGA_patient_barcode** | **Project ID** | **Sample_type** |
|---|---|---|
| TCGA-24-0968 | TCGA-OV | Primary Solid tumor |
| TCGA-24-1419 | TCGA-OV | Primary Solid tumor |
| TCGA-24-2036 | TCGA-OV | Primary Solid tumor |
| TCGA-23-2077 | TCGA-OV | Primary Solid tumor |
| TCGA-13-1497 | TCGA-OV | Primary Solid tumor |
| TCGA-24-1469 | TCGA-OV | Primary Solid tumor |
| TCGA-57-1993 | TCGA-OV | Primary Solid tumor |
| TCGA-25-1321 | TCGA-OV | Primary Solid tumor |
| TCGA-30-1862 | TCGA-OV | Primary Solid tumor |
| TCGA-23-1114 | TCGA-OV | Primary Solid tumor |
| TCGA-24-1424 | TCGA-OV | Primary Solid tumor |
| TCGA-13-1477 | TCGA-OV | Primary Solid tumor |
| TCGA-24-1425 | TCGA-OV | Primary Solid tumor |
| TCGA-29-1762 | TCGA-OV | Primary Solid tumor |
| TCGA-13-0714 | TCGA-OV | Primary Solid tumor |
| TCGA-13-1499 | TCGA-OV | Primary Solid tumor |
| TCGA-61-2102 | TCGA-OV | Primary Solid tumor |
| TCGA-24-1428 | TCGA-OV | Primary Solid tumor |
| TCGA-04-1357 | TCGA-OV | Primary Solid tumor |
| TCGA-24-1560 | TCGA-OV | Primary Solid tumor |
| TCGA-59-2351 | TCGA-OV | Primary Solid tumor |
| TCGA-13-0908 | TCGA-OV | Primary Solid tumor |
| TCGA-61-1738 | TCGA-OV | Primary Solid tumor |
| TCGA-24-1930 | TCGA-OV | Primary Solid tumor |
| TCGA-13-1411 | TCGA-OV | Primary Solid tumor |
| TCGA-61-1728 | TCGA-OV | Primary Solid tumor |
| TCGA-25-2392 | TCGA-OV | Primary Solid tumor |
| TCGA-10-0936 | TCGA-OV | Primary Solid tumor |
| TCGA-25-1631 | TCGA-OV | Primary Solid tumor |
| TCGA-09-1659 | TCGA-OV | Primary Solid tumor |
| TCGA-24-0982 | TCGA-OV | Primary Solid tumor |
| TCGA-09-2045 | TCGA-OV | Primary Solid tumor |
| TCGA-25-1623 | TCGA-OV | Primary Solid tumor |
| TCGA-23-1113 | TCGA-OV | Primary Solid tumor |
| TCGA-13-0884 | TCGA-OV | Primary Solid tumor |
| TCGA-13-1501 | TCGA-OV | Primary Solid tumor |
| TCGA-25-1877 | TCGA-OV | Primary Solid tumor |
| TCGA-13-0766 | TCGA-OV | Primary Solid tumor |
| TCGA-23-1029 | TCGA-OV | Primary Solid tumor |
| TCGA-59-2350 | TCGA-OV | Primary Solid tumor |
| TCGA-30-1714 | TCGA-OV | Primary Solid tumor |
| TCGA-13-0795 | TCGA-OV | Primary Solid tumor |
| TCGA-13-0765 | TCGA-OV | Primary Solid tumor |
| TCGA-23-1030 | TCGA-OV | Primary Solid tumor |
| TCGA-13-1506 | TCGA-OV | Primary Solid tumor |
| TCGA-29-1769 | TCGA-OV | Primary Solid tumor |

| TCGA_patient_barcode | Project ID | Sample_type |
|---|---|---|
| TCGA-24-1616 | TCGA-OV | Primary Solid tumor |
| TCGA-25-2396 | TCGA-OV | Primary Solid tumor |
| TCGA-13-0804 | TCGA-OV | Primary Solid tumor |
| TCGA-13-0920 | TCGA-OV | Primary Solid tumor |
| TCGA-29-1710 | TCGA-OV | Primary Solid tumor |
| TCGA-13-0905 | TCGA-OV | Primary Solid tumor |
| TCGA-24-1467 | TCGA-OV | Primary Solid tumor |
| TCGA-24-2023 | TCGA-OV | Primary Solid tumor |
| TCGA-61-2098 | TCGA-OV | Primary Solid tumor |
| TCGA-31-1944 | TCGA-OV | Primary Solid tumor |
| TCGA-57-1583 | TCGA-OV | Primary Solid tumor |
| TCGA-24-1928 | TCGA-OV | Primary Solid tumor |
| TCGA-30-1718 | TCGA-OV | Primary Solid tumor |
| TCGA-23-1111 | TCGA-OV | Primary Solid tumor |
| TCGA-13-0916 | TCGA-OV | Primary Solid tumor |
| TCGA-23-1120 | TCGA-OV | Primary Solid tumor |
| TCGA-24-1474 | TCGA-OV | Primary Solid tumor |
| TCGA-24-2261 | TCGA-OV | Primary Solid tumor |
| TCGA-61-1736 | TCGA-OV | Primary Solid tumor |
| TCGA-13-0911 | TCGA-OV | Primary Solid tumor |
| TCGA-29-1766 | TCGA-OV | Primary Solid tumor |
| TCGA-09-0369 | TCGA-OV | Primary Solid tumor |
| TCGA-13-1496 | TCGA-OV | Primary Solid tumor |
| TCGA-25-1318 | TCGA-OV | Primary Solid tumor |
| TCGA-36-1577 | TCGA-OV | Primary Solid tumor |
| TCGA-29-1711 | TCGA-OV | Primary Solid tumor |
| TCGA-29-1770 | TCGA-OV | Primary Solid tumor |
| TCGA-24-1550 | TCGA-OV | Primary Solid tumor |
| TCGA-23-1023 | TCGA-OV | Primary Solid tumor |
| TCGA-25-1632 | TCGA-OV | Primary Solid tumor |
| TCGA-13-1488 | TCGA-OV | Primary Solid tumor |
| TCGA-13-0724 | TCGA-OV | Primary Solid tumor |
| TCGA-59-2355 | TCGA-OV | Primary Solid tumor |
| TCGA-29-1696 | TCGA-OV | Primary Solid tumor |
| TCGA-61-2003 | TCGA-OV | Primary Solid tumor |
| TCGA-20-1682 | TCGA-OV | Primary Solid tumor |
| TCGA-13-1511 | TCGA-OV | Primary Solid tumor |
| TCGA-61-2101 | TCGA-OV | Primary Solid tumor |
| TCGA-29-1695 | TCGA-OV | Primary Solid tumor |
| TCGA-24-1105 | TCGA-OV | Primary Solid tumor |
| TCGA-23-1024 | TCGA-OV | Primary Solid tumor |
| TCGA-29-1761 | TCGA-OV | Primary Solid tumor |
| TCGA-29-1691 | TCGA-OV | Primary Solid tumor |
| TCGA-09-1665 | TCGA-OV | Primary Solid tumor |
| TCGA-24-1426 | TCGA-OV | Primary Solid tumor |
| TCGA-24-2038 | TCGA-OV | Primary Solid tumor |

| TCGA_patient_barcode | Project ID | Sample_type |
|---|---|---|
| TCGA-09-1661 | TCGA-OV | Primary Solid tumor |
| TCGA-23-1809 | TCGA-OV | Primary Solid tumor |
| TCGA-61-1919 | TCGA-OV | Primary Solid tumor |
| TCGA-13-1498 | TCGA-OV | Primary Solid tumor |
| TCGA-31-1953 | TCGA-OV | Primary Solid tumor |
| TCGA-AX-A06H | TCGA-UCEC | Primary Solid tumor |
| TCGA-A5-A0GN | TCGA-UCEC | Primary Solid tumor |
| TCGA-A5-A0VO | TCGA-UCEC | Primary Solid tumor |
| TCGA-B5-A1N2 | TCGA-UCEC | Primary Solid tumor |
| TCGA-BG-A187 | TCGA-UCEC | Primary Solid tumor |
| TCGA-B5-A11Z | TCGA-UCEC | Primary Solid tumor |
| TCGA-DF-A2KU | TCGA-UCEC | Primary Solid tumor |
| TCGA-D1-A101 | TCGA-UCEC | Primary Solid tumor |
| TCGA-EC-A24G | TCGA-UCEC | Primary Solid tumor |
| TCGA-D1-A15W | TCGA-UCEC | Primary Solid tumor |
| TCGA-AX-A2IN | TCGA-UCEC | Primary Solid tumor |
| TCGA-D1-A2G7 | TCGA-UCEC | Primary Solid tumor |
| TCGA-D1-A16G | TCGA-UCEC | Primary Solid tumor |
| TCGA-AP-A3K1 | TCGA-UCEC | Primary Solid tumor |
| TCGA-AX-A0IZ | TCGA-UCEC | Primary Solid tumor |
| TCGA-BK-A13C | TCGA-UCEC | Primary Solid tumor |
| TCGA-BG-A0M0 | TCGA-UCEC | Primary Solid tumor |
| TCGA-D1-A179 | TCGA-UCEC | Primary Solid tumor |
| TCGA-BS-A0VI | TCGA-UCEC | Primary Solid tumor |
| TCGA-FI-A3PV | TCGA-UCEC | Primary Solid tumor |
| TCGA-D1-A17N | TCGA-UCEC | Primary Solid tumor |
| TCGA-PG-A916 | TCGA-UCEC | Primary Solid tumor |
| TCGA-BS-A0U9 | TCGA-UCEC | Primary Solid tumor |
| TCGA-BG-A0VZ | TCGA-UCEC | Primary Solid tumor |
| TCGA-BG-A0M9 | TCGA-UCEC | Primary Solid tumor |
| TCGA-AP-A0LM | TCGA-UCEC | Primary Solid tumor |
| TCGA-A5-A0GU | TCGA-UCEC | Primary Solid tumor |
| TCGA-EY-A1GR | TCGA-UCEC | Primary Solid tumor |
| TCGA-AX-A0IW | TCGA-UCEC | Primary Solid tumor |
| TCGA-BG-A0M2 | TCGA-UCEC | Primary Solid tumor |
| TCGA-AX-A1CI | TCGA-UCEC | Primary Solid tumor |
| TCGA-EO-A3KX | TCGA-UCEC | Primary Solid tumor |
| TCGA-AJ-A3EM | TCGA-UCEC | Primary Solid tumor |
| TCGA-AJ-A3NG | TCGA-UCEC | Primary Solid tumor |
| TCGA-A5-A7WK | TCGA-UCEC | Primary Solid tumor |
| TCGA-B5-A0K2 | TCGA-UCEC | Primary Solid tumor |
| TCGA-BG-A0MI | TCGA-UCEC | Primary Solid tumor |
| TCGA-BG-A18C | TCGA-UCEC | Primary Solid tumor |
| TCGA-AX-A05S | TCGA-UCEC | Primary Solid tumor |
| TCGA-BG-A0W2 | TCGA-UCEC | Primary Solid tumor |
| TCGA-AP-A0LD | TCGA-UCEC | Primary Solid tumor |

| TCGA_patient_barcode | Project ID | Sample_type |
|---|---|---|
| TCGA-AX-A2HG | TCGA-UCEC | Primary Solid tumor |
| TCGA-H5-A2HR | TCGA-UCEC | Primary Solid tumor |
| TCGA-D1-A169 | TCGA-UCEC | Primary Solid tumor |
| TCGA-EC-A1NJ | TCGA-UCEC | Primary Solid tumor |
| TCGA-AJ-A23M | TCGA-UCEC | Primary Solid tumor |
| TCGA-AX-A1CC | TCGA-UCEC | Primary Solid tumor |
| TCGA-SL-A6J9 | TCGA-UCEC | Primary Solid tumor |
| TCGA-AX-A3G6 | TCGA-UCEC | Primary Solid tumor |
| TCGA-AX-A3FZ | TCGA-UCEC | Primary Solid tumor |
| TCGA-D1-A16Q | TCGA-UCEC | Primary Solid tumor |
| TCGA-D1-A16J | TCGA-UCEC | Primary Solid tumor |
| TCGA-B5-A0K1 | TCGA-UCEC | Primary Solid tumor |
| TCGA-AP-A1E4 | TCGA-UCEC | Primary Solid tumor |
| TCGA-B5-A0KB | TCGA-UCEC | Primary Solid tumor |
| TCGA-B5-A0K6 | TCGA-UCEC | Primary Solid tumor |
| TCGA-PG-A915 | TCGA-UCEC | Primary Solid tumor |
| TCGA-PG-A5BC | TCGA-UCEC | Primary Solid tumor |
| TCGA-E6-A1M0 | TCGA-UCEC | Primary Solid tumor |
| TCGA-AJ-A3NH | TCGA-UCEC | Primary Solid tumor |
| TCGA-EO-A1Y7 | TCGA-UCEC | Primary Solid tumor |
| TCGA-B5-A11S | TCGA-UCEC | Primary Solid tumor |
| TCGA-AP-A059 | TCGA-UCEC | Primary Solid tumor |
| TCGA-PG-A6IB | TCGA-UCEC | Primary Solid tumor |
| TCGA-BG-A0M4 | TCGA-UCEC | Primary Solid tumor |
| TCGA-AX-A1CJ | TCGA-UCEC | Primary Solid tumor |
| TCGA-AX-A2H4 | TCGA-UCEC | Primary Solid tumor |
| TCGA-BS-A0TI | TCGA-UCEC | Primary Solid tumor |
| TCGA-EO-A3L0 | TCGA-UCEC | Primary Solid tumor |
| TCGA-B5-A3F9 | TCGA-UCEC | Primary Solid tumor |
| TCGA-AJ-A2QO | TCGA-UCEC | Primary Solid tumor |
| TCGA-D1-A0ZO | TCGA-UCEC | Primary Solid tumor |
| TCGA-AX-A05Z | TCGA-UCEC | Primary Solid tumor |
| TCGA-D1-A17L | TCGA-UCEC | Primary Solid tumor |
| TCGA-A5-A0GQ | TCGA-UCEC | Primary Solid tumor |
| TCGA-AX-A3G1 | TCGA-UCEC | Primary Solid tumor |
| TCGA-BK-A0CC | TCGA-UCEC | Primary Solid tumor |
| TCGA-D1-A1O8 | TCGA-UCEC | Primary Solid tumor |
| TCGA-DI-A1NN | TCGA-UCEC | Primary Solid tumor |
| TCGA-B5-A0K9 | TCGA-UCEC | Primary Solid tumor |
| TCGA-FI-A2CY | TCGA-UCEC | Primary Solid tumor |
| TCGA-AP-A1DR | TCGA-UCEC | Primary Solid tumor |
| TCGA-B5-A11Q | TCGA-UCEC | Primary Solid tumor |
| TCGA-BS-A0U7 | TCGA-UCEC | Primary Solid tumor |
| TCGA-A5-A0VQ | TCGA-UCEC | Primary Solid tumor |
| TCGA-BS-A0TE | TCGA-UCEC | Primary Solid tumor |
| TCGA-BS-A0T9 | TCGA-UCEC | Primary Solid tumor |

| **TCGA_patient_barcode** | **Project ID** | **Sample_type** |
|---|---|---|
| TCGA-AW-A1PO | TCGA-UCEC | Primary Solid tumor |
| TCGA-AJ-A3I9 | TCGA-UCEC | Primary Solid tumor |
| TCGA-AJ-A8CV | TCGA-UCEC | Primary Solid tumor |
| TCGA-B5-A121 | TCGA-UCEC | Primary Solid tumor |
| TCGA-AX-A1CN | TCGA-UCEC | Primary Solid tumor |
| TCGA-BK-A139 | TCGA-UCEC | Primary Solid tumor |
| TCGA-EY-A1G7 | TCGA-UCEC | Primary Solid tumor |
| TCGA-BK-A6W3 | TCGA-UCEC | Primary Solid tumor |
| TCGA-FI-A2CX | TCGA-UCEC | Primary Solid tumor |
| TCGA-DI-A2QU | TCGA-UCEC | Primary Solid tumor |
| TCGA-AP-A0LT | TCGA-UCEC | Primary Solid tumor |
| TCGA-EO-A3AS | TCGA-UCEC | Primary Solid tumor |
| TCGA-E6-A1LZ | TCGA-UCEC | Primary Solid tumor |
| TCGA-AP-A1DO | TCGA-UCEC | Primary Solid tumor |
| TCGA-AX-A2HC | TCGA-UCEC | Primary Solid tumor |
| TCGA-EC-A1QX | TCGA-UCEC | Primary Solid tumor |
| TCGA-BG-A2AE | TCGA-UCEC | Primary Solid tumor |
| TCGA-5S-A9Q8 | TCGA-UCEC | Primary Solid tumor |
| TCGA-AP-A0LS | TCGA-UCEC | Primary Solid tumor |
| TCGA-D1-A1O5 | TCGA-UCEC | Primary Solid tumor |
| TCGA-EY-A2OQ | TCGA-UCEC | Primary Solid tumor |
| TCGA-AX-A1C9 | TCGA-UCEC | Primary Solid tumor |
| TCGA-D1-A16R | TCGA-UCEC | Primary Solid tumor |
| TCGA-AX-A0IS | TCGA-UCEC | Primary Solid tumor |
| TCGA-EY-A1G8 | TCGA-UCEC | Primary Solid tumor |
| TCGA-EY-A1GW | TCGA-UCEC | Primary Solid tumor |
| TCGA-BG-A0RY | TCGA-UCEC | Primary Solid tumor |
| TCGA-D1-A1NY | TCGA-UCEC | Primary Solid tumor |
| TCGA-B5-A3S1 | TCGA-UCEC | Primary Solid tumor |
| TCGA-A5-A2K5 | TCGA-UCEC | Primary Solid tumor |
| TCGA-AX-A062 | TCGA-UCEC | Primary Solid tumor |
| TCGA-BS-A0WQ | TCGA-UCEC | Primary Solid tumor |
| TCGA-FI-A2EY | TCGA-UCEC | Primary Solid tumor |
| TCGA-A5-A0R8 | TCGA-UCEC | Primary Solid tumor |
| TCGA-A5-A0G3 | TCGA-UCEC | Primary Solid tumor |
| TCGA-AP-A054 | TCGA-UCEC | Primary Solid tumor |
| TCGA-A5-A0GX | TCGA-UCEC | Primary Solid tumor |
| TCGA-DI-A2QY | TCGA-UCEC | Primary Solid tumor |
| TCGA-EY-A1GC | TCGA-UCEC | Primary Solid tumor |
| TCGA-A5-A0GI | TCGA-UCEC | Primary Solid tumor |
| TCGA-D1-A17S | TCGA-UCEC | Primary Solid tumor |
| TCGA-EY-A5W2 | TCGA-UCEC | Primary Solid tumor |
| TCGA-D1-A1NU | TCGA-UCEC | Primary Solid tumor |
| TCGA-BG-A18A | TCGA-UCEC | Primary Solid tumor |
| TCGA-BK-A26L | TCGA-UCEC | Primary Solid tumor |
| TCGA-D1-A0ZU | TCGA-UCEC | Primary Solid tumor |

| TCGA_patient_barcode | Project ID | Sample_type |
| --- | --- | --- |
| TCGA-AP-A1E1 | TCGA-UCEC | Primary Solid tumor |
| TCGA-KP-A3VZ | TCGA-UCEC | Primary Solid tumor |
| TCGA-AP-A05O | TCGA-UCEC | Primary Solid tumor |
| TCGA-AJ-A3BF | TCGA-UCEC | Primary Solid tumor |
| TCGA-BG-A0VT | TCGA-UCEC | Primary Solid tumor |
| TCGA-AP-A0L9 | TCGA-UCEC | Primary Solid tumor |
| TCGA-D1-A0ZP | TCGA-UCEC | Primary Solid tumor |
| TCGA-EY-A1GT | TCGA-UCEC | Primary Solid tumor |
| TCGA-BK-A0CC | TCGA-UCEC | Primary Solid tumor |
| TCGA-D1-A16Y | TCGA-UCEC | Primary Solid tumor |
| TCGA-A5-A0R7 | TCGA-UCEC | Primary Solid tumor |
| TCGA-BG-A186 | TCGA-UCEC | Primary Solid tumor |
| TCGA-BS-A0TD | TCGA-UCEC | Primary Solid tumor |
| TCGA-EY-A2ON | TCGA-UCEC | Primary Solid tumor |
| TCGA-AJ-A3OJ | TCGA-UCEC | Primary Solid tumor |
| TCGA-BS-A0TJ | TCGA-UCEC | Primary Solid tumor |
| TCGA-K6-A3WQ | TCGA-UCEC | Primary Solid tumor |
| TCGA-D1-A3JP | TCGA-UCEC | Primary Solid tumor |
| TCGA-QS-A744 | TCGA-UCEC | Primary Solid tumor |
| TCGA-BK-A4ZD | TCGA-UCEC | Primary Solid tumor |
| TCGA-D1-A160 | TCGA-UCEC | Primary Solid tumor |
| TCGA-A5-A3LP | TCGA-UCEC | Primary Solid tumor |
| TCGA-D1-A176 | TCGA-UCEC | Primary Solid tumor |
| TCGA-AJ-A3BG | TCGA-UCEC | Primary Solid tumor |
| TCGA-AX-A3FT | TCGA-UCEC | Primary Solid tumor |
| TCGA-AX-A1C7 | TCGA-UCEC | Primary Solid tumor |
| TCGA-D1-A16D | TCGA-UCEC | Primary Solid tumor |
| TCGA-EO-A3KU | TCGA-UCEC | Primary Solid tumor |
| TCGA-D1-A1NZ | TCGA-UCEC | Primary Solid tumor |
| TCGA-AP-A1DM | TCGA-UCEC | Primary Solid tumor |
| TCGA-D1-A103 | TCGA-UCEC | Primary Solid tumor |
| TCGA-FI-A2D4 | TCGA-UCEC | Primary Solid tumor |
| TCGA-BK-A0CC | TCGA-UCEC | Primary Solid tumor |
| TCGA-BS-A0TC | TCGA-UCEC | Primary Solid tumor |
| TCGA-B5-A1MS | TCGA-UCEC | Primary Solid tumor |
| TCGA-DF-A2KZ | TCGA-UCEC | Primary Solid tumor |
| TCGA-B5-A11P | TCGA-UCEC | Primary Solid tumor |
| TCGA-A5-A0GW | TCGA-UCEC | Primary Solid tumor |
| TCGA-EO-A2CH | TCGA-UCEC | Primary Solid tumor |
| TCGA-A5-A3LO | TCGA-UCEC | Primary Solid tumor |
| TCGA-EO-A3B1 | TCGA-UCEC | Primary Solid tumor |
| TCGA-AJ-A3NC | TCGA-UCEC | Primary Solid tumor |
| TCGA-B5-A11U | TCGA-UCEC | Primary Solid tumor |
| TCGA-D1-A168 | TCGA-UCEC | Primary Solid tumor |
| TCGA-AP-A0LV | TCGA-UCEC | Primary Solid tumor |
| TCGA-A5-A0VP | TCGA-UCEC | Primary Solid tumor |

| TCGA_patient_barcode | Project ID | Sample_type |
|---|---|---|
| TCGA-BK-A0C9 | TCGA-UCEC | Primary Solid tumor |
| TCGA-D1-A16N | TCGA-UCEC | Primary Solid tumor |
| TCGA-D1-A17Q | TCGA-UCEC | Primary Solid tumor |
| TCGA-SJ-A6ZJ | TCGA-UCEC | Primary Solid tumor |
| TCGA-AX-A06D | TCGA-UCEC | Primary Solid tumor |
| TCGA-BG-A0MS | TCGA-UCEC | Primary Solid tumor |
| TCGA-AX-A2IO | TCGA-UCEC | Primary Solid tumor |
| TCGA-A5-A0GH | TCGA-UCEC | Primary Solid tumor |
| TCGA-AX-A2HJ | TCGA-UCEC | Primary Solid tumor |
| TCGA-QF-A5YS | TCGA-UCEC | Primary Solid tumor |
| TCGA-AX-A3G4 | TCGA-UCEC | Primary Solid tumor |
| TCGA-B5-A0K3 | TCGA-UCEC | Primary Solid tumor |
| TCGA-B5-A11R | TCGA-UCEC | Primary Solid tumor |
| TCGA-D1-A17K | TCGA-UCEC | Primary Solid tumor |
| TCGA-D1-A17T | TCGA-UCEC | Primary Solid tumor |
| TCGA-5B-A90C | TCGA-UCEC | Primary Solid tumor |
| TCGA-A5-A0G1 | TCGA-UCEC | Primary Solid tumor |
| TCGA-EY-A549 | TCGA-UCEC | Primary Solid tumor |
| TCGA-FI-A2D0 | TCGA-UCEC | Primary Solid tumor |
| TCGA-BS-A0V7 | TCGA-UCEC | Primary Solid tumor |
| TCGA-B5-A1MW | TCGA-UCEC | Primary Solid tumor |
| TCGA-D1-A17D | TCGA-UCEC | Primary Solid tumor |
| TCGA-BS-A0TA | TCGA-UCEC | Primary Solid tumor |
| TCGA-PG-A917 | TCGA-UCEC | Primary Solid tumor |
| TCGA-AX-A3FS | TCGA-UCEC | Primary Solid tumor |
| TCGA-A5-A2K4 | TCGA-UCEC | Primary Solid tumor |
| TCGA-D1-A1O0 | TCGA-UCEC | Primary Solid tumor |
| TCGA-B5-A0JS | TCGA-UCEC | Primary Solid tumor |
| TCGA-SL-A6JA | TCGA-UCEC | Primary Solid tumor |
| TCGA-EO-A22T | TCGA-UCEC | Primary Solid tumor |
| TCGA-B5-A11V | TCGA-UCEC | Primary Solid tumor |
| TCGA-AP-A051 | TCGA-UCEC | Primary Solid tumor |
| TCGA-AJ-A2QL | TCGA-UCEC | Primary Solid tumor |
| TCGA-A5-A1OK | TCGA-UCEC | Primary Solid tumor |
| TCGA-B5-A11W | TCGA-UCEC | Primary Solid tumor |
| TCGA-D1-A0ZQ | TCGA-UCEC | Primary Solid tumor |
| TCGA-DF-A2L0 | TCGA-UCEC | Primary Solid tumor |
| TCGA-B5-A11N | TCGA-UCEC | Primary Solid tumor |
| TCGA-EY-A2OM | TCGA-UCEC | Primary Solid tumor |
| TCGA-D1-A16I | TCGA-UCEC | Primary Solid tumor |
| TCGA-AJ-A8CT | TCGA-UCEC | Primary Solid tumor |
| TCGA-AX-A06B | TCGA-UCEC | Primary Solid tumor |
| TCGA-E6-A2P9 | TCGA-UCEC | Primary Solid tumor |
| TCGA-BK-A56F | TCGA-UCEC | Primary Solid tumor |
| TCGA-B5-A0K7 | TCGA-UCEC | Primary Solid tumor |
| TCGA-AX-A0J1 | TCGA-UCEC | Primary Solid tumor |

| TCGA_patient_barcode | Project ID | Sample_type |
|---|---|---|
| TCGA-AJ-A3NF | TCGA-UCEC | Primary Solid tumor |
| TCGA-AJ-A3EJ | TCGA-UCEC | Primary Solid tumor |
| TCGA-BK-A26L | TCGA-UCEC | Primary Solid tumor |
| TCGA-BS-A0UF | TCGA-UCEC | Primary Solid tumor |
| TCGA-EY-A2OO | TCGA-UCEC | Primary Solid tumor |
| TCGA-BS-A0UM | TCGA-UCEC | Primary Solid tumor |
| TCGA-AP-A0LJ | TCGA-UCEC | Primary Solid tumor |
| TCGA-EO-A1Y8 | TCGA-UCEC | Primary Solid tumor |
| TCGA-AX-A05T | TCGA-UCEC | Primary Solid tumor |
| TCGA-AP-A0LI | TCGA-UCEC | Primary Solid tumor |
| TCGA-D1-A0ZS | TCGA-UCEC | Primary Solid tumor |
| TCGA-AX-A1CF | TCGA-UCEC | Primary Solid tumor |
| TCGA-B5-A0JZ | TCGA-UCEC | Primary Solid tumor |
| TCGA-A5-A0GA | TCGA-UCEC | Primary Solid tumor |
| TCGA-AJ-A23N | TCGA-UCEC | Primary Solid tumor |
| TCGA-A5-A0GP | TCGA-UCEC | Primary Solid tumor |
| TCGA-AX-A1CP | TCGA-UCEC | Primary Solid tumor |
| TCGA-BS-A0UV | TCGA-UCEC | Primary Solid tumor |
| TCGA-DF-A2KS | TCGA-UCEC | Primary Solid tumor |
| TCGA-AP-A05J | TCGA-UCEC | Primary Solid tumor |
| TCGA-AJ-A8CW | TCGA-UCEC | Primary Solid tumor |
| TCGA-A5-A1OF | TCGA-UCEC | Primary Solid tumor |
| TCGA-AX-A1C5 | TCGA-UCEC | Primary Solid tumor |
| TCGA-B5-A1MV | TCGA-UCEC | Primary Solid tumor |
| TCGA-D1-A174 | TCGA-UCEC | Primary Solid tumor |
| TCGA-D1-A2G5 | TCGA-UCEC | Primary Solid tumor |
| TCGA-FI-A2EX | TCGA-UCEC | Primary Solid tumor |
| TCGA-AP-A05A | TCGA-UCEC | Primary Solid tumor |
| TCGA-EY-A1GE | TCGA-UCEC | Primary Solid tumor |
| TCGA-A5-A7WJ | TCGA-UCEC | Primary Solid tumor |
| TCGA-A5-A1OH | TCGA-UCEC | Primary Solid tumor |
| TCGA-B5-A0JU | TCGA-UCEC | Primary Solid tumor |
| TCGA-AX-A3FX | TCGA-UCEC | Primary Solid tumor |
| TCGA-D1-A163 | TCGA-UCEC | Primary Solid tumor |
| TCGA-B5-A3FA | TCGA-UCEC | Primary Solid tumor |
| TCGA-AP-A1DV | TCGA-UCEC | Primary Solid tumor |
| TCGA-BG-A0MT | TCGA-UCEC | Primary Solid tumor |
| TCGA-E6-A2P8 | TCGA-UCEC | Primary Solid tumor |
| TCGA-AJ-A3OK | TCGA-UCEC | Primary Solid tumor |
| TCGA-FI-A2EW | TCGA-UCEC | Primary Solid tumor |
| TCGA-D1-A165 | TCGA-UCEC | Primary Solid tumor |
| TCGA-BG-A222 | TCGA-UCEC | Primary Solid tumor |
| TCGA-PG-A914 | TCGA-UCEC | Primary Solid tumor |
| TCGA-AX-A1CE | TCGA-UCEC | Primary Solid tumor |
| TCGA-D1-A17F | TCGA-UCEC | Primary Solid tumor |
| TCGA-EO-A3AV | TCGA-UCEC | Primary Solid tumor |

| TCGA_patient_barcode | Project ID | Sample_type |
|---|---|---|
| TCGA-D1-A17B | TCGA-UCEC | Primary Solid tumor |
| TCGA-E6-A8L9 | TCGA-UCEC | Primary Solid tumor |
| TCGA-4E-A92E | TCGA-UCEC | Primary Solid tumor |
| TCGA-AP-A1DK | TCGA-UCEC | Primary Solid tumor |
| TCGA-AP-A05P | TCGA-UCEC | Primary Solid tumor |
| TCGA-EO-A3KW | TCGA-UCEC | Primary Solid tumor |
| TCGA-B5-A0K0 | TCGA-UCEC | Primary Solid tumor |
| TCGA-A5-A1OG | TCGA-UCEC | Primary Solid tumor |
| TCGA-AJ-A2QN | TCGA-UCEC | Primary Solid tumor |
| TCGA-AX-A1CK | TCGA-UCEC | Primary Solid tumor |
| TCGA-BG-A0LW | TCGA-UCEC | Primary Solid tumor |
| TCGA-B5-A11H | TCGA-UCEC | Primary Solid tumor |
| TCGA-AX-A2HH | TCGA-UCEC | Primary Solid tumor |
| TCGA-B5-A5OE | TCGA-UCEC | Primary Solid tumor |
| TCGA-BG-A0M6 | TCGA-UCEC | Primary Solid tumor |
| TCGA-B5-A11Y | TCGA-UCEC | Primary Solid tumor |
| TCGA-AJ-A3IA | TCGA-UCEC | Primary Solid tumor |
| TCGA-D1-A1NS | TCGA-UCEC | Primary Solid tumor |
| TCGA-AX-A2H2 | TCGA-UCEC | Primary Solid tumor |
| TCGA-D1-A162 | TCGA-UCEC | Primary Solid tumor |
| TCGA-AP-A0LG | TCGA-UCEC | Primary Solid tumor |
| TCGA-DF-A2KN | TCGA-UCEC | Primary Solid tumor |
| TCGA-AX-A1C4 | TCGA-UCEC | Primary Solid tumor |
| TCGA-BK-A139 | TCGA-UCEC | Primary Solid tumor |
| TCGA-AX-A3G9 | TCGA-UCEC | Primary Solid tumor |
| TCGA-B5-A0JR | TCGA-UCEC | Primary Solid tumor |
| TCGA-BG-A221 | TCGA-UCEC | Primary Solid tumor |
| TCGA-FI-A2EU | TCGA-UCEC | Primary Solid tumor |
| TCGA-A5-AB3J | TCGA-UCEC | Primary Solid tumor |
| TCGA-A5-A0G2 | TCGA-UCEC | Primary Solid tumor |
| TCGA-FI-A2D2 | TCGA-UCEC | Primary Solid tumor |
| TCGA-AP-A056 | TCGA-UCEC | Primary Solid tumor |
| TCGA-D1-A17R | TCGA-UCEC | Primary Solid tumor |
| TCGA-AX-A060 | TCGA-UCEC | Primary Solid tumor |
| TCGA-BS-A0UT | TCGA-UCEC | Primary Solid tumor |
| TCGA-AX-A2H7 | TCGA-UCEC | Primary Solid tumor |
| TCGA-A5-A2K3 | TCGA-UCEC | Primary Solid tumor |
| TCGA-AP-A05H | TCGA-UCEC | Primary Solid tumor |
| TCGA-AX-A3FV | TCGA-UCEC | Primary Solid tumor |
| TCGA-D1-A16F | TCGA-UCEC | Primary Solid tumor |
| TCGA-JU-AAVI | TCGA-UCEC | Primary Solid tumor |
| TCGA-AX-A06L | TCGA-UCEC | Primary Solid tumor |
| TCGA-BG-A3PP | TCGA-UCEC | Primary Solid tumor |
| TCGA-B5-A1MU | TCGA-UCEC | Primary Solid tumor |
| TCGA-A5-A0RA | TCGA-UCEC | Primary Solid tumor |
| TCGA-BG-A0MK | TCGA-UCEC | Primary Solid tumor |

| TCGA_patient_barcode | Project ID | Sample_type |
|---|---|---|
| TCGA-A5-A0GB | TCGA-UCEC | Primary Solid tumor |
| TCGA-AX-A2HA | TCGA-UCEC | Primary Solid tumor |
| TCGA-EY-A1GH | TCGA-UCEC | Primary Solid tumor |
| TCGA-AJ-A3EL | TCGA-UCEC | Primary Solid tumor |
| TCGA-EY-A3QX | TCGA-UCEC | Primary Solid tumor |
| TCGA-B5-A11F | TCGA-UCEC | Primary Solid tumor |
| TCGA-EY-A1GX | TCGA-UCEC | Primary Solid tumor |
| TCGA-D1-A15V | TCGA-UCEC | Primary Solid tumor |
| TCGA-AP-A0LO | TCGA-UCEC | Primary Solid tumor |
| TCGA-D1-A1NX | TCGA-UCEC | Primary Solid tumor |
| TCGA-B5-A0JV | TCGA-UCEC | Primary Solid tumor |
| TCGA-AP-A5FX | TCGA-UCEC | Primary Solid tumor |
| TCGA-AJ-A3EK | TCGA-UCEC | Primary Solid tumor |
| TCGA-AX-A1C8 | TCGA-UCEC | Primary Solid tumor |
| TCGA-AX-A0IU | TCGA-UCEC | Primary Solid tumor |
| TCGA-A5-A0R6 | TCGA-UCEC | Primary Solid tumor |
| TCGA-BG-A0LX | TCGA-UCEC | Primary Solid tumor |
| TCGA-B5-A11E | TCGA-UCEC | Primary Solid tumor |
| TCGA-BG-A0W1 | TCGA-UCEC | Primary Solid tumor |
| TCGA-AP-A05N | TCGA-UCEC | Primary Solid tumor |
| TCGA-BS-A0U5 | TCGA-UCEC | Primary Solid tumor |
| TCGA-QF-A5YT | TCGA-UCEC | Primary Solid tumor |
| TCGA-AJ-A5DV | TCGA-UCEC | Primary Solid tumor |
| TCGA-AX-A3G3 | TCGA-UCEC | Primary Solid tumor |
| TCGA-EY-A210 | TCGA-UCEC | Primary Solid tumor |
| TCGA-EY-A1GO | TCGA-UCEC | Primary Solid tumor |
| TCGA-QS-A8F1 | TCGA-UCEC | Primary Solid tumor |
| TCGA-AJ-A2QK | TCGA-UCEC | Primary Solid tumor |
| TCGA-AJ-A3BI | TCGA-UCEC | Primary Solid tumor |
| TCGA-BK-A0CA | TCGA-UCEC | Primary Solid tumor |
| TCGA-AX-A064 | TCGA-UCEC | Primary Solid tumor |
| TCGA-AP-A053 | TCGA-UCEC | Primary Solid tumor |
| TCGA-DI-A1NO | TCGA-UCEC | Primary Solid tumor |
| TCGA-FI-A3PX | TCGA-UCEC | Primary Solid tumor |
| TCGA-BS-A0V4 | TCGA-UCEC | Primary Solid tumor |
| TCGA-AP-A1DQ | TCGA-UCEC | Primary Solid tumor |
| TCGA-AP-A1E0 | TCGA-UCEC | Primary Solid tumor |
| TCGA-A5-A0GM | TCGA-UCEC | Primary Solid tumor |
| TCGA-AX-A0J0 | TCGA-UCEC | Primary Solid tumor |
| TCGA-AX-A3G8 | TCGA-UCEC | Primary Solid tumor |
| TCGA-A5-A0G9 | TCGA-UCEC | Primary Solid tumor |
| TCGA-BG-A0MG | TCGA-UCEC | Primary Solid tumor |
| TCGA-B5-A0K4 | TCGA-UCEC | Primary Solid tumor |
| TCGA-PG-A7D5 | TCGA-UCEC | Primary Solid tumor |
| TCGA-SJ-A6ZI | TCGA-UCEC | Primary Solid tumor |
| TCGA-FI-A2F9 | TCGA-UCEC | Primary Solid tumor |

| TCGA_patient_barcode | Project ID | Sample_type |
|---|---|---|
| TCGA-B5-A0JY | TCGA-UCEC | Primary Solid tumor |
| TCGA-EO-A22Y | TCGA-UCEC | Primary Solid tumor |
| TCGA-B5-A1MX | TCGA-UCEC | Primary Solid tumor |
| TCGA-FI-A2F8 | TCGA-UCEC | Primary Solid tumor |
| TCGA-AX-A1CA | TCGA-UCEC | Primary Solid tumor |
| TCGA-EY-A1GQ | TCGA-UCEC | Primary Solid tumor |
| TCGA-DI-A1BY | TCGA-UCEC | Primary Solid tumor |
| TCGA-BG-A2AD | TCGA-UCEC | Primary Solid tumor |
| TCGA-EO-A3B0 | TCGA-UCEC | Primary Solid tumor |
| TCGA-D1-A16X | TCGA-UCEC | Primary Solid tumor |
| TCGA-EY-A1H0 | TCGA-UCEC | Primary Solid tumor |
| TCGA-B5-A3FD | TCGA-UCEC | Primary Solid tumor |
| TCGA-BG-A0YU | TCGA-UCEC | Primary Solid tumor |
| TCGA-EO-A3AY | TCGA-UCEC | Primary Solid tumor |
| TCGA-EY-A548 | TCGA-UCEC | Primary Solid tumor |
| TCGA-D1-A16E | TCGA-UCEC | Primary Solid tumor |
| TCGA-AJ-A3NE | TCGA-UCEC | Primary Solid tumor |
| TCGA-B5-A1MR | TCGA-UCEC | Primary Solid tumor |
| TCGA-B5-A11X | TCGA-UCEC | Primary Solid tumor |
| TCGA-AX-A3GI | TCGA-UCEC | Primary Solid tumor |
| TCGA-AP-A0LL | TCGA-UCEC | Primary Solid tumor |
| TCGA-BG-A18B | TCGA-UCEC | Primary Solid tumor |
| TCGA-EY-A1GF | TCGA-UCEC | Primary Solid tumor |
| TCGA-EY-A1GD | TCGA-UCEC | Primary Solid tumor |
| TCGA-AP-A1DH | TCGA-UCEC | Primary Solid tumor |
| TCGA-B5-A11J | TCGA-UCEC | Primary Solid tumor |
| TCGA-EO-A22S | TCGA-UCEC | Primary Solid tumor |
| TCGA-BG-A0VX | TCGA-UCEC | Primary Solid tumor |
| TCGA-E6-A1LX | TCGA-UCEC | Primary Solid tumor |
| TCGA-A5-A0GD | TCGA-UCEC | Primary Solid tumor |
| TCGA-D1-A17H | TCGA-UCEC | Primary Solid tumor |
| TCGA-AX-A2H8 | TCGA-UCEC | Primary Solid tumor |
| TCGA-AX-A05W | TCGA-UCEC | Primary Solid tumor |
| TCGA-D1-A1O7 | TCGA-UCEC | Primary Solid tumor |
| TCGA-B5-A5OD | TCGA-UCEC | Primary Solid tumor |
| TCGA-QS-A5YR | TCGA-UCEC | Primary Solid tumor |
| TCGA-B5-A0JN | TCGA-UCEC | Primary Solid tumor |
| TCGA-D1-A17A | TCGA-UCEC | Primary Solid tumor |
| TCGA-BG-A0VW | TCGA-UCEC | Primary Solid tumor |
| TCGA-BG-A0MC | TCGA-UCEC | Primary Solid tumor |
| TCGA-D1-A2G0 | TCGA-UCEC | Primary Solid tumor |
| TCGA-BG-A0M7 | TCGA-UCEC | Primary Solid tumor |
| TCGA-D1-A177 | TCGA-UCEC | Primary Solid tumor |
| TCGA-B5-A3FH | TCGA-UCEC | Primary Solid tumor |
| TCGA-KJ-A3U4 | TCGA-UCEC | Primary Solid tumor |
| TCGA-B5-A3FB | TCGA-UCEC | Primary Solid tumor |

| **TCGA_patient_barcode** | **Project ID** | **Sample_type** |
|---|---|---|
| TCGA-B5-A11I | TCGA-UCEC | Primary Solid tumor |
| TCGA-EO-A3AZ | TCGA-UCEC | Primary Solid tumor |
| TCGA-BG-A0YV | TCGA-UCEC | Primary Solid tumor |
| TCGA-EY-A3L3 | TCGA-UCEC | Primary Solid tumor |
| TCGA-BK-A6W4 | TCGA-UCEC | Primary Solid tumor |
| TCGA-AX-A05Y | TCGA-UCEC | Primary Solid tumor |
| TCGA-DF-A2KY | TCGA-UCEC | Primary Solid tumor |
| TCGA-BG-A3EW | TCGA-UCEC | Primary Solid tumor |
| TCGA-AX-A2HD | TCGA-UCEC | Primary Solid tumor |
| TCGA-AJ-A3TW | TCGA-UCEC | Primary Solid tumor |
| TCGA-D1-A16O | TCGA-UCEC | Primary Solid tumor |
| TCGA-FI-A2D5 | TCGA-UCEC | Primary Solid tumor |
| TCGA-A5-A0GR | TCGA-UCEC | Primary Solid tumor |
| TCGA-D1-A0ZN | TCGA-UCEC | Primary Solid tumor |
| TCGA-BS-A0TG | TCGA-UCEC | Primary Solid tumor |
| TCGA-BG-A0MO | TCGA-UCEC | Primary Solid tumor |
| TCGA-D1-A15X | TCGA-UCEC | Primary Solid tumor |
| TCGA-B5-A0K8 | TCGA-UCEC | Primary Solid tumor |
| TCGA-DI-A1BU | TCGA-UCEC | Primary Solid tumor |
| TCGA-EY-A1GI | TCGA-UCEC | Primary Solid tumor |
| TCGA-EY-A215 | TCGA-UCEC | Primary Solid tumor |
| TCGA-D1-A3DG | TCGA-UCEC | Primary Solid tumor |
| TCGA-EO-A22U | TCGA-UCEC | Primary Solid tumor |
| TCGA-AX-A3FW | TCGA-UCEC | Primary Solid tumor |
| TCGA-AP-A1DP | TCGA-UCEC | Primary Solid tumor |
| TCGA-A5-A0GG | TCGA-UCEC | Primary Solid tumor |
| TCGA-AP-A05D | TCGA-UCEC | Primary Solid tumor |
| TCGA-KP-A3W3 | TCGA-UCEC | Primary Solid tumor |
| TCGA-B5-A5OC | TCGA-UCEC | Primary Solid tumor |
| TCGA-BG-A0MU | TCGA-UCEC | Primary Solid tumor |
| TCGA-D1-A0ZV | TCGA-UCEC | Primary Solid tumor |
| TCGA-EO-A22X | TCGA-UCEC | Primary Solid tumor |
| TCGA-B5-A11O | TCGA-UCEC | Primary Solid tumor |
| TCGA-QS-A5YQ | TCGA-UCEC | Primary Solid tumor |
| TCGA-EY-A54A | TCGA-UCEC | Primary Solid tumor |
| TCGA-D1-A3DH | TCGA-UCEC | Primary Solid tumor |
| TCGA-B5-A0JX | TCGA-UCEC | Primary Solid tumor |
| TCGA-B5-A0JT | TCGA-UCEC | Primary Solid tumor |
| TCGA-A5-A0GJ | TCGA-UCEC | Primary Solid tumor |
| TCGA-BG-A220 | TCGA-UCEC | Primary Solid tumor |
| TCGA-EY-A1GP | TCGA-UCEC | Primary Solid tumor |
| TCGA-D1-A102 | TCGA-UCEC | Primary Solid tumor |
| TCGA-BS-A0UL | TCGA-UCEC | Primary Solid tumor |
| TCGA-BG-A0MA | TCGA-UCEC | Primary Solid tumor |
| TCGA-EO-A1Y5 | TCGA-UCEC | Primary Solid tumor |
| TCGA-EY-A1GL | TCGA-UCEC | Primary Solid tumor |

| TCGA_patient_barcode | Project ID | Sample_type |
|---|---|---|
| TCGA-A5-A2K7 | TCGA-UCEC | Primary Solid tumor |
| TCGA-DI-A2QT | TCGA-UCEC | Primary Solid tumor |
| TCGA-AJ-A5DW | TCGA-UCEC | Primary Solid tumor |
| TCGA-AP-A052 | TCGA-UCEC | Primary Solid tumor |
| TCGA-AJ-A3BH | TCGA-UCEC | Primary Solid tumor |
| TCGA-B5-A11G | TCGA-UCEC | Primary Solid tumor |
| TCGA-EY-A1GV | TCGA-UCEC | Primary Solid tumor |
| TCGA-D1-A175 | TCGA-UCEC | Primary Solid tumor |
| TCGA-AX-A2H5 | TCGA-UCEC | Primary Solid tumor |
| TCGA-B5-A3FC | TCGA-UCEC | Primary Solid tumor |
| TCGA-BS-A0UA | TCGA-UCEC | Primary Solid tumor |
| TCGA-AP-A0L8 | TCGA-UCEC | Primary Solid tumor |
| TCGA-D1-A161 | TCGA-UCEC | Primary Solid tumor |
| TCGA-BK-A139 | TCGA-UCEC | Primary Solid tumor |
| TCGA-D1-A17U | TCGA-UCEC | Primary Solid tumor |
| TCGA-BS-A0V6 | TCGA-UCEC | Primary Solid tumor |
| TCGA-EY-A1GU | TCGA-UCEC | Primary Solid tumor |
| TCGA-EO-A2CG | TCGA-UCEC | Primary Solid tumor |
| TCGA-AJ-A2QM | TCGA-UCEC | Primary Solid tumor |
| TCGA-AP-A0LF | TCGA-UCEC | Primary Solid tumor |
| TCGA-D1-A17M | TCGA-UCEC | Primary Solid tumor |
| TCGA-A5-A0R9 | TCGA-UCEC | Primary Solid tumor |
| TCGA-A5-A0GV | TCGA-UCEC | Primary Solid tumor |
| TCGA-B5-A1MZ | TCGA-UCEC | Primary Solid tumor |
| TCGA-D1-A1NW | TCGA-UCEC | Primary Solid tumor |
| TCGA-AJ-A3BD | TCGA-UCEC | Primary Solid tumor |
| TCGA-AJ-A23O | TCGA-UCEC | Primary Solid tumor |
| TCGA-DF-A2KR | TCGA-UCEC | Primary Solid tumor |
| TCGA-KP-A3W1 | TCGA-UCEC | Primary Solid tumor |
| TCGA-D1-A2G6 | TCGA-UCEC | Primary Solid tumor |
| TCGA-BG-A0M3 | TCGA-UCEC | Primary Solid tumor |
| TCGA-AX-A06J | TCGA-UCEC | Primary Solid tumor |
| TCGA-KP-A3W4 | TCGA-UCEC | Primary Solid tumor |
| TCGA-FI-A2D6 | TCGA-UCEC | Primary Solid tumor |
| TCGA-BG-A0MQ | TCGA-UCEC | Primary Solid tumor |
| TCGA-BG-A0VV | TCGA-UCEC | Primary Solid tumor |
| TCGA-D1-A16V | TCGA-UCEC | Primary Solid tumor |
| TCGA-EO-A22R | TCGA-UCEC | Primary Solid tumor |
| TCGA-BG-A0MH | TCGA-UCEC | Primary Solid tumor |
| TCGA-EY-A2OP | TCGA-UCEC | Primary Solid tumor |
| TCGA-B5-A11L | TCGA-UCEC | Primary Solid tumor |
| TCGA-D1-A0ZR | TCGA-UCEC | Primary Solid tumor |
| TCGA-EY-A72D | TCGA-UCEC | Primary Solid tumor |
| TCGA-B5-A11M | TCGA-UCEC | Primary Solid tumor |
| TCGA-EY-A1GS | TCGA-UCEC | Primary Solid tumor |
| TCGA-BK-A0CB | TCGA-UCEC | Primary Solid tumor |

| TCGA_patient_barcode | Project ID | Sample_type |
|---|---|---|
| TCGA-KP-A3W0 | TCGA-UCEC | Primary Solid tumor |
| TCGA-DF-A2KV | TCGA-UCEC | Primary Solid tumor |
| TCGA-D1-A17C | TCGA-UCEC | Primary Solid tumor |
| TCGA-D1-A15Z | TCGA-UCEC | Primary Solid tumor |
| TCGA-EY-A547 | TCGA-UCEC | Primary Solid tumor |
| TCGA-D1-A16B | TCGA-UCEC | Primary Solid tumor |
| TCGA-BS-A0V8 | TCGA-UCEC | Primary Solid tumor |
| TCGA-AX-A05U | TCGA-UCEC | Primary Solid tumor |
| TCGA-AP-A0LE | TCGA-UCEC | Primary Solid tumor |
| TCGA-EY-A212 | TCGA-UCEC | Primary Solid tumor |
| TCGA-D1-A3DA | TCGA-UCEC | Primary Solid tumor |
| TCGA-AJ-A3BK | TCGA-UCEC | Primary Solid tumor |
| TCGA-AP-A1E3 | TCGA-UCEC | Primary Solid tumor |
| TCGA-BS-A0UJ | TCGA-UCEC | Primary Solid tumor |
| TCGA-AX-A3GB | TCGA-UCEC | Primary Solid tumor |
| TCGA-BK-A26L | TCGA-UCEC | Primary Solid tumor |
| TCGA-AX-A2HF | TCGA-UCEC | Primary Solid tumor |
| TCGA-A5-A2K2 | TCGA-UCEC | Primary Solid tumor |
| TCGA-D1-A3JQ | TCGA-UCEC | Primary Solid tumor |
| TCGA-BG-A0M8 | TCGA-UCEC | Primary Solid tumor |
| TCGA-EY-A1GM | TCGA-UCEC | Primary Solid tumor |
| TCGA-EY-A4KR | TCGA-UCEC | Primary Solid tumor |
| TCGA-D1-A16S | TCGA-UCEC | Primary Solid tumor |
| TCGA-AX-A1CR | TCGA-UCEC | Primary Solid tumor |
| TCGA-AJ-A3QS | TCGA-UCEC | Primary Solid tumor |
| TCGA-2E-A9G8 | TCGA-UCEC | Primary Solid tumor |
| TCGA-A5-A0GE | TCGA-UCEC | Primary Solid tumor |
| TCGA-AP-A0LP | TCGA-UCEC | Primary Solid tumor |
| TCGA-B5-A1MY | TCGA-UCEC | Primary Solid tumor |
| TCGA-A5-A1OJ | TCGA-UCEC | Primary Solid tumor |
| TCGA-EY-A1GK | TCGA-UCEC | Primary Solid tumor |
| TCGA-BS-A0U8 | TCGA-UCEC | Primary Solid tumor |
| TCGA-BK-A0CA | TCGA-UCEC | Primary Solid tumor |
| TCGA-AP-A0LN | TCGA-UCEC | Primary Solid tumor |
| TCGA-BG-A2L7 | TCGA-UCEC | Primary Solid tumor |
| TCGA-BK-A13B | TCGA-UCEC | Primary Solid tumor |
| TCGA-BK-A0CA | TCGA-UCEC | Primary Solid tumor |
| TCGA-AX-A2HK | TCGA-UCEC | Primary Solid tumor |
| TCGA-AX-A06F | TCGA-UCEC | Primary Solid tumor |
| TCGA-D1-A0ZZ | TCGA-UCEC | Primary Solid tumor |
| TCGA-AJ-A3OL | TCGA-UCEC | Primary Solid tumor |
| TCGA-AX-A063 | TCGA-UCEC | Primary Solid tumor |
| TCGA-DI-A0WH | TCGA-UCEC | Primary Solid tumor |
| TCGA-D1-A167 | TCGA-UCEC | Primary Solid tumor |
| TCGA-EO-A3AU | TCGA-UCEC | Primary Solid tumor |
| TCGA-EY-A214 | TCGA-UCEC | Primary Solid tumor |

| TCGA_patient_barcode | Project ID | Sample_type |
|---|---|---|
| TCGA-A5-A0G5 | TCGA-UCEC | Primary Solid tumor |
| TCGA-AP-A0LH | TCGA-UCEC | Primary Solid tumor |
| TCGA-FI-A2F4 | TCGA-UCEC | Primary Solid tumor |
| TCGA-AX-A3G7 | TCGA-UCEC | Primary Solid tumor |
| TCGA-E9-A1NA | TCGA-BRCA | Solid tissue normal |
| TCGA-BH-A18L | TCGA-BRCA | Solid tissue normal |
| TCGA-BH-A1F0 | TCGA-BRCA | Solid tissue normal |
| TCGA-BH-A208 | TCGA-BRCA | Solid tissue normal |
| TCGA-E2-A15M | TCGA-BRCA | Solid tissue normal |
| TCGA-A7-A0CH | TCGA-BRCA | Solid tissue normal |
| TCGA-E2-A1BC | TCGA-BRCA | Solid tissue normal |
| TCGA-BH-A18P | TCGA-BRCA | Solid tissue normal |
| TCGA-BH-A0B7 | TCGA-BRCA | Solid tissue normal |
| TCGA-BH-A18M | TCGA-BRCA | Solid tissue normal |
| TCGA-BH-A0DZ | TCGA-BRCA | Solid tissue normal |
| TCGA-E2-A1LB | TCGA-BRCA | Solid tissue normal |
| TCGA-BH-A1FU | TCGA-BRCA | Solid tissue normal |
| TCGA-E2-A1LH | TCGA-BRCA | Solid tissue normal |
| TCGA-A7-A0DB | TCGA-BRCA | Solid tissue normal |
| TCGA-BH-A203 | TCGA-BRCA | Solid tissue normal |
| TCGA-BH-A0H7 | TCGA-BRCA | Solid tissue normal |
| TCGA-BH-A0BA | TCGA-BRCA | Solid tissue normal |
| TCGA-BH-A0DT | TCGA-BRCA | Solid tissue normal |
| TCGA-A7-A13G | TCGA-BRCA | Solid tissue normal |
| TCGA-E9-A1RF | TCGA-BRCA | Solid tissue normal |
| TCGA-BH-A0DK | TCGA-BRCA | Solid tissue normal |
| TCGA-AC-A2FM | TCGA-BRCA | Solid tissue normal |
| TCGA-BH-A0DV | TCGA-BRCA | Solid tissue normal |
| TCGA-BH-A1FJ | TCGA-BRCA | Solid tissue normal |
| TCGA-BH-A18N | TCGA-BRCA | Solid tissue normal |
| TCGA-BH-A0DP | TCGA-BRCA | Solid tissue normal |
| TCGA-A7-A0D9 | TCGA-BRCA | Solid tissue normal |
| TCGA-BH-A1F2 | TCGA-BRCA | Solid tissue normal |
| TCGA-BH-A0BM | TCGA-BRCA | Solid tissue normal |
| TCGA-E9-A1RD | TCGA-BRCA | Solid tissue normal |
| TCGA-BH-A0DG | TCGA-BRCA | Solid tissue normal |
| TCGA-BH-A1FB | TCGA-BRCA | Solid tissue normal |
| TCGA-BH-A18R | TCGA-BRCA | Solid tissue normal |
| TCGA-E2-A153 | TCGA-BRCA | Solid tissue normal |
| TCGA-A7-A0CE | TCGA-BRCA | Solid tissue normal |
| TCGA-BH-A0DO | TCGA-BRCA | Solid tissue normal |
| TCGA-E9-A1RH | TCGA-BRCA | Solid tissue normal |
| TCGA-E9-A1N4 | TCGA-BRCA | Solid tissue normal |
| TCGA-E9-A1NF | TCGA-BRCA | Solid tissue normal |
| TCGA-BH-A0HK | TCGA-BRCA | Solid tissue normal |
| TCGA-BH-A1FC | TCGA-BRCA | Solid tissue normal |

| **TCGA_patient_barcode** | **Project ID** | **Sample_type** |
|---|---|---|
| TCGA-BH-A0BW | TCGA-BRCA | Solid tissue normal |
| TCGA-BH-A1EO | TCGA-BRCA | Solid tissue normal |
| TCGA-BH-A0B8 | TCGA-BRCA | Solid tissue normal |
| TCGA-E9-A1RI | TCGA-BRCA | Solid tissue normal |
| TCGA-BH-A0BJ | TCGA-BRCA | Solid tissue normal |
| TCGA-A7-A13E | TCGA-BRCA | Solid tissue normal |
| TCGA-BH-A0AY | TCGA-BRCA | Solid tissue normal |
| TCGA-A7-A0DC | TCGA-BRCA | Solid tissue normal |
| TCGA-BH-A1F8 | TCGA-BRCA | Solid tissue normal |
| TCGA-BH-A18J | TCGA-BRCA | Solid tissue normal |
| TCGA-E2-A1LS | TCGA-BRCA | Solid tissue normal |
| TCGA-BH-A0DQ | TCGA-BRCA | Solid tissue normal |
| TCGA-BH-A0BS | TCGA-BRCA | Solid tissue normal |
| TCGA-E9-A1RB | TCGA-BRCA | Solid tissue normal |
| TCGA-BH-A1EU | TCGA-BRCA | Solid tissue normal |
| TCGA-BH-A0E1 | TCGA-BRCA | Solid tissue normal |
| TCGA-AC-A23H | TCGA-BRCA | Solid tissue normal |
| TCGA-BH-A0HA | TCGA-BRCA | Solid tissue normal |
| TCGA-BH-A1F6 | TCGA-BRCA | Solid tissue normal |
| TCGA-BH-A0AZ | TCGA-BRCA | Solid tissue normal |
| TCGA-BH-A18S | TCGA-BRCA | Solid tissue normal |
| TCGA-BH-A0DH | TCGA-BRCA | Solid tissue normal |
| TCGA-BH-A1ET | TCGA-BRCA | Solid tissue normal |
| TCGA-E9-A1RC | TCGA-BRCA | Solid tissue normal |
| TCGA-BH-A0C3 | TCGA-BRCA | Solid tissue normal |
| TCGA-BH-A1EN | TCGA-BRCA | Solid tissue normal |
| TCGA-E9-A1N9 | TCGA-BRCA | Solid tissue normal |
| TCGA-BH-A18U | TCGA-BRCA | Solid tissue normal |
| TCGA-BH-A0BV | TCGA-BRCA | Solid tissue normal |
| TCGA-E2-A1L7 | TCGA-BRCA | Solid tissue normal |
| TCGA-BH-A1FE | TCGA-BRCA | Solid tissue normal |
| TCGA-BH-A0BC | TCGA-BRCA | Solid tissue normal |
| TCGA-BH-A0B3 | TCGA-BRCA | Solid tissue normal |
| TCGA-E9-A1R7 | TCGA-BRCA | Solid tissue normal |
| TCGA-BH-A18K | TCGA-BRCA | Solid tissue normal |
| TCGA-BH-A1FD | TCGA-BRCA | Solid tissue normal |
| TCGA-E9-A1N5 | TCGA-BRCA | Solid tissue normal |
| TCGA-BH-A18Q | TCGA-BRCA | Solid tissue normal |
| TCGA-AC-A2FB | TCGA-BRCA | Solid tissue normal |
| TCGA-E2-A15I | TCGA-BRCA | Solid tissue normal |
| TCGA-E2-A1IG | TCGA-BRCA | Solid tissue normal |
| TCGA-BH-A18V | TCGA-BRCA | Solid tissue normal |
| TCGA-BH-A0DD | TCGA-BRCA | Solid tissue normal |
| TCGA-BH-A0BZ | TCGA-BRCA | Solid tissue normal |
| TCGA-BH-A1FM | TCGA-BRCA | Solid tissue normal |
| TCGA-BH-A1EV | TCGA-BRCA | Solid tissue normal |

| TCGA_patient_barcode | Project ID | Sample_type |
|---|---|---|
| TCGA-AC-A2FF | TCGA-BRCA | Solid tissue normal |
| TCGA-E2-A158 | TCGA-BRCA | Solid tissue normal |
| TCGA-BH-A1FG | TCGA-BRCA | Solid tissue normal |
| TCGA-BH-A0BQ | TCGA-BRCA | Solid tissue normal |
| TCGA-BH-A1FH | TCGA-BRCA | Solid tissue normal |
| TCGA-BH-A0H9 | TCGA-BRCA | Solid tissue normal |
| TCGA-E2-A15K | TCGA-BRCA | Solid tissue normal |
| TCGA-BH-A0AU | TCGA-BRCA | Solid tissue normal |
| TCGA-BH-A0E0 | TCGA-BRCA | Solid tissue normal |
| TCGA-BH-A1FR | TCGA-BRCA | Solid tissue normal |
| TCGA-BH-A0B5 | TCGA-BRCA | Solid tissue normal |
| TCGA-BH-A0DL | TCGA-BRCA | Solid tissue normal |
| TCGA-GI-A2C9 | TCGA-BRCA | Solid tissue normal |
| TCGA-BH-A0H5 | TCGA-BRCA | Solid tissue normal |
| TCGA-BH-A1EW | TCGA-BRCA | Solid tissue normal |
| TCGA-BH-A204 | TCGA-BRCA | Solid tissue normal |
| TCGA-BH-A1FN | TCGA-BRCA | Solid tissue normal |
| TCGA-BH-A0BT | TCGA-BRCA | Solid tissue normal |
| TCGA-E9-A1NG | TCGA-BRCA | Solid tissue normal |
| TCGA-E9-A1N6 | TCGA-BRCA | Solid tissue normal |
| TCGA-BH-A209 | TCGA-BRCA | Solid tissue normal |
| TCGA-A7-A13F | TCGA-BRCA | Solid tissue normal |
| TCGA-E9-A1ND | TCGA-BRCA | Solid tissue normal |
| TCGA-GI-A2C8 | TCGA-BRCA | Solid tissue normal |
| TCGA-BH-A0C0 | TCGA-BRCA | Solid tissue normal |
| TCGA-AX-A2HD | TCGA-UCEC | Solid tissue normal |
| TCGA-AX-A2H8 | TCGA-UCEC | Solid tissue normal |
| TCGA-AX-A1CI | TCGA-UCEC | Solid tissue normal |
| TCGA-AX-A1CF | TCGA-UCEC | Solid tissue normal |
| TCGA-BG-A2AD | TCGA-UCEC | Solid tissue normal |
| TCGA-FL-A1YN | TCGA-UCEC | Solid tissue normal |
| TCGA-FL-A1YV | TCGA-UCEC | Solid tissue normal |
| TCGA-AX-A2HA | TCGA-UCEC | Solid tissue normal |
| TCGA-BK-A0CB | TCGA-UCEC | Solid tissue normal |
| TCGA-FL-A3WE | TCGA-UCEC | Solid tissue normal |
| TCGA-FL-A1YI | TCGA-UCEC | Solid tissue normal |
| TCGA-AJ-A2QL | TCGA-UCEC | Solid tissue normal |
| TCGA-DI-A2QY | TCGA-UCEC | Solid tissue normal |
| TCGA-AX-A2HC | TCGA-UCEC | Solid tissue normal |
| TCGA-AJ-A3NC | TCGA-UCEC | Solid tissue normal |
| TCGA-FL-A1YT | TCGA-UCEC | Solid tissue normal |
| TCGA-E6-A1M0 | TCGA-UCEC | Solid tissue normal |
| TCGA-BK-A13C | TCGA-UCEC | Solid tissue normal |
| TCGA-FL-A1YU | TCGA-UCEC | Solid tissue normal |
| TCGA-AJ-A3NE | TCGA-UCEC | Solid tissue normal |
| TCGA-AX-A0IZ | TCGA-UCEC | Solid tissue normal |

| TCGA_patient_barcode | Project ID | Sample_type |
|---|---|---|
| TCGA-AJ-A3NH | TCGA-UCEC | Solid tissue normal |
| TCGA-FL-A1YH | TCGA-UCEC | Solid tissue normal |
| TCGA-FL-A1YF | TCGA-UCEC | Solid tissue normal |
| TCGA-FL-A1YQ | TCGA-UCEC | Solid tissue normal |
| TCGA-BG-A3EW | TCGA-UCEC | Solid tissue normal |
| TCGA-BG-A3PP | TCGA-UCEC | Solid tissue normal |
| TCGA-BK-A4ZD | TCGA-UCEC | Solid tissue normal |
| TCGA-AX-A05Y | TCGA-UCEC | Solid tissue normal |
| TCGA-AX-A0J0 | TCGA-UCEC | Solid tissue normal |
| TCGA-FL-A1YM | TCGA-UCEC | Solid tissue normal |
| TCGA-FL-A1YG | TCGA-UCEC | Solid tissue normal |
| TCGA-FL-A1YL | TCGA-UCEC | Solid tissue normal |
| TCGA-AX-A1CK | TCGA-UCEC | Solid tissue normal |
| TCGA-DI-A2QU | TCGA-UCEC | Solid tissue normal |

**Supplementary Table 2.** Enrichment score values of the common 1642 signature genes between the three female cancers and normal samples. Threshold ≥1.3 [-log(p–value)] Fisher's Exact Test p–value.

| **Canonical Pathways** | **Enrichment score** |
|---|---|
| Kinetochore Metaphase Signaling Pathway | 19.13931761 |
| Role of CHK Proteins in Cell Cycle Checkpoint Control | 7.576513144 |
| Role of BRCA1 in DNA Damage Response | 7.150578895 |
| Cell Cycle Control of Chromosomal Replication | 6.908158406 |
| Mitotic Roles of Polo-Like Kinase | 6.491957902 |
| Estrogen-mediated S-phase Entry | 6.319902359 |
| Agranulocyte Adhesion and Diapedesis | 5.936186052 |
| Molecular Mechanisms of Cancer | 5.722276488 |
| ILK Signaling | 5.182795888 |
| Cell Cycle: G2/M DNA Damage Checkpoint Regulation | 4.716056577 |
| Cell Cycle Regulation by BTG Family Proteins | 4.582944085 |
| Hereditary Breast Cancer Signaling | 4.514828915 |
| Cyclins and Cell Cycle Regulation | 4.506532796 |
| Senescence Pathway | 4.471757483 |
| ATM Signaling | 3.991004946 |
| Granulocyte Adhesion and Diapedesis | 3.950478824 |
| Interferon Signaling | 3.939399786 |
| Pancreatic Adenocarcinoma Signaling | 3.801645996 |
| DNA damage-induced 14-3-3σ Signaling | 3.752188303 |
| Regulation Of The Epithelial Mesenchymal Transition By Growth Factors Pathway | 3.632307627 |
| RhoA Signaling | 3.547785669 |
| Signaling by Rho Family GTPases | 3.449964811 |
| RAR Activation | 3.409779613 |
| Mismatch Repair in Eukaryotes | 3.329223554 |
| Hepatic Fibrosis Signaling Pathway | 3.295452951 |
| Production of Nitric Oxide and Reactive Oxygen Species in Macrophages | 3.263695241 |
| IL-7 Signaling Pathway | 3.08429672 |
| Role of Macrophages. Fibroblasts and Endothelial Cells in Rheumatoid Arthritis | 3.037840189 |
| Atherosclerosis Signaling | 2.9454672 |
| IL-17A Signaling in Gastric Cells | 2.931679003 |
| Nitric Oxide Signaling in the Cardiovascular System | 2.926755336 |
| Oxidative Phosphorylation | 2.896794493 |
| GP6 Signaling Pathway | 2.876315179 |
| Acute Phase Response Signaling | 2.869671528 |
| IL-8 Signaling | 2.861299253 |
| Protein Kinase A Signaling | 2.84126427 |
| Mitochondrial Dysfunction | 2.792883687 |
| Inhibition of Angiogenesis by TSP1 | 2.740593425 |
| Dendritic Cell Maturation | 2.737972575 |
| B Cell Receptor Signaling | 2.674232944 |

| | |
|---|---|
| Corticotropin Releasing Hormone Signaling | 2.632824219 |
| Adrenomedullin signaling pathway | 2.629223777 |
| Leukocyte Extravasation Signaling | 2.629223777 |
| UVB-Induced MAPK Signaling | 2.583315143 |
| CXCR4 Signaling | 2.582381101 |
| Axonal Guidance Signaling | 2.54441526 |
| p53 Signaling | 2.541105829 |
| Osteoarthritis Pathway | 2.532674642 |
| Sperm Motility | 2.500184014 |
| Apelin Cardiomyocyte Signaling Pathway | 2.497707174 |
| Growth Hormone Signaling | 2.495259095 |
| Cholecystokinin/Gastrin-mediated Signaling | 2.480267239 |
| Glioma Signaling | 2.445287561 |
| Inhibition of Matrix Metalloproteases | 2.343801285 |
| NER Pathway | 2.33155495 |
| Sumoylation Pathway | 2.33155495 |
| HER-2 Signaling in Breast Cancer | 2.315692791 |
| Glioblastoma Multiforme Signaling | 2.314191465 |
| Systemic Lupus Erythematosus In B Cell Signaling Pathway | 2.31049896 |
| Erythropoietin Signaling | 2.248407889 |
| Cell Cycle: G1/S Checkpoint Regulation | 2.232905014 |
| Glucocorticoid Receptor Signaling | 2.231995144 |
| Tight Junction Signaling | 2.224202625 |
| 14-3-3-mediated Signaling | 2.190255063 |
| BER pathway | 2.146463128 |
| Glycolysis I | 2.128967502 |
| Role of JAK2 in Hormone-like Cytokine Signaling | 2.119111497 |
| IL-3 Signaling | 2.113008049 |
| GADD45 Signaling | 2.097326701 |
| Sertoli Cell-Sertoli Cell Junction Signaling | 2.052555208 |
| Thrombin Signaling | 2.031605911 |
| Prolactin Signaling | 2.027545773 |
| IL-12 Signaling and Production in Macrophages | 2.025928817 |
| Role of Osteoblasts. Osteoclasts and Chondrocytes in Rheumatoid Arthritis | 2.012808412 |
| Thrombopoietin Signaling | 1.973787719 |
| Phospholipase C Signaling | 1.949727139 |
| STAT3 Pathway | 1.933026727 |
| Estrogen Receptor Signaling | 1.931897182 |
| Phagosome Formation | 1.913585668 |
| IL-6 Signaling | 1.913585668 |
| Glioma Invasiveness Signaling | 1.911043361 |
| Fcγ Receptor-mediated Phagocytosis in Macrophages and Monocytes | 1.90871435 |
| EGF Signaling | 1.908076358 |
| Apelin Endothelial Signaling Pathway | 1.896507074 |
| RhoGDI Signaling | 1.895167213 |
| Aldosterone Signaling in Epithelial Cells | 1.893464738 |

| | |
|---|---|
| DNA Double-Strand Break Repair by Homologous Recombination | 1.888760488 |
| PTEN Signaling | 1.882426439 |
| FXR/RXR Activation | 1.882426439 |
| Regulation of the Epithelial-Mesenchymal Transition Pathway | 1.874519629 |
| Oxidized GTP and dGTP Detoxification | 1.865785698 |
| L-serine Degradation | 1.865785698 |
| NADH Repair | 1.865785698 |
| Docosahexaenoic Acid (DHA) Signaling | 1.852892306 |
| P2Y Purigenic Receptor Signaling Pathway | 1.851749201 |
| α-Adrenergic Signaling | 1.836481622 |
| HIF1α Signaling | 1.833783653 |
| Role of PKR in Interferon Induction and Antiviral Response | 1.832169152 |
| Role of Tissue Factor in Cancer | 1.832169152 |
| Breast Cancer Regulation by Stathmin1 | 1.831146048 |
| GDNF Family Ligand-Receptor Interactions | 1.828450725 |
| Pyrimidine Deoxyribonucleotides De Novo Biosynthesis I | 1.817039284 |
| Renin-Angiotensin Signaling | 1.800791865 |
| White Adipose Tissue Browning Pathway | 1.791807082 |
| GNRH Signaling | 1.790297303 |
| UVA-Induced MAPK Signaling | 1.766974685 |
| Semaphorin Neuronal Repulsive Signaling Pathway | 1.7625259 |
| Agrin Interactions at Neuromuscular Junction | 1.749579507 |
| Hepatic Fibrosis / Hepatic Stellate Cell Activation | 1.74760953 |
| Cellular Effects of Sildenafil (Viagra) | 1.733694097 |
| Type II Diabetes Mellitus Signaling | 1.731534315 |
| Thyroid Cancer Signaling | 1.711473368 |
| BEX2 Signaling Pathway | 1.711473368 |
| IL-15 Production | 1.709708709 |
| Role of Pattern Recognition Receptors in Recognition of Bacteria and Viruses | 1.706279824 |
| Endothelin-1 Signaling | 1.70073317 |
| Pathogenesis of Multiple Sclerosis | 1.696751353 |
| Synaptic Long Term Depression | 1.67771237 |
| Opioid Signaling Pathway | 1.676592443 |
| IL-22 Signaling | 1.658671774 |
| UVC-Induced MAPK Signaling | 1.645440216 |
| IL-9 Signaling | 1.625095019 |
| Gαq Signaling | 1.605539062 |
| Chronic Myeloid Leukemia Signaling | 1.604346523 |
| LPS-stimulated MAPK Signaling | 1.602180708 |
| Proline Biosynthesis I | 1.5850592 |
| IGF-1 Signaling | 1.57361987 |
| PPAR Signaling | 1.57361987 |
| Iron homeostasis signaling pathway | 1.569722994 |
| Systemic Lupus Erythematosus Signaling | 1.567575428 |
| HOTAIR Regulatory Pathway | 1.557208147 |
| Actin Cytoskeleton Signaling | 1.552518207 |

| | |
|---|---|
| ErbB Signaling | 1.551963782 |
| FGF Signaling | 1.533282274 |
| Sirtuin Signaling Pathway | 1.525312899 |
| Relaxin Signaling | 1.525053371 |
| Estrogen-Dependent Breast Cancer Signaling | 1.51873673 |
| Apelin Liver Signaling Pathway | 1.518330652 |
| Hepatic Cholestasis | 1.510843663 |
| Virus Entry via Endocytic Pathways | 1.484809403 |
| Angiopoietin Signaling | 1.483223091 |
| Gap Junction Signaling | 1.482464271 |
| PXR/RXR Activation | 1.471798593 |
| FAT10 Cancer Signaling Pathway | 1.431749587 |
| Aryl Hydrocarbon Receptor Signaling | 1.42014168 |
| RANK Signaling in Osteoclasts | 1.404262124 |
| Notch Signaling | 1.403976313 |
| AMPK Signaling | 1.399717562 |
| VDR/RXR Activation | 1.381683347 |
| HGF Signaling | 1.373827786 |
| Inflammasome pathway | 1.346634234 |
| NAD biosynthesis II (from tryptophan) | 1.345868234 |
| JAK/Stat Signaling | 1.317922159 |
| Renal Cell Carcinoma Signaling | 1.317922159 |
| Prostate Cancer Signaling | 1.314601239 |
| Maturity Onset Diabetes of Young (MODY) Signaling | 1.311893572 |
| CREB Signaling in Neurons | 1.307176349 |
| April Mediated Signaling | 1.306949012 |

**Supplementary table 3.** IPA networks underlying the 1642 Ensembl IDs ranked for score statistics.

| ID | Molecules in Network | Score | Focus Molecules | Top Diseases and Functions |
|---|---|---|---|---|
| 1 | ABRACL,ASPM,B4GALT3,CDC14B,CDC42EP3,CDCA2,CDCA7,CDKN3,CHST3,EPSTI1,**ERBB2**,ESPL1,GBE1,GINS1,HEPH,HSD17B11,LIG1,MFAP2,MXD3,MYO10,NCAPD2,NCAPG,NCAPH,NUP210,PHLDB1,PLAAT4,POLE2,POLQ,POLR3K,PORCN,RAD51AP1,RFC2,SMTN,TSPAN13,UNC5B | 38 | 35 | Cancer, Cardiovascular Disease, DNA Replication, Recombination, and Repair |
| 2 | BATF2,Cadherin,CCNF,CDC45,CDC7,CDT1,CHEK1,CIT,DBF4,DONSON,E2F8,ECT2,ESCO2,FBXO31,GINS4,H2AZ1,HSH2D,HSPB6,KIF14,KIF23,KIF4A,LAMA3,LDH (family),LRWD1,MCM2,MCM4,P38 MAPK,PBK,PRC1,RACGAP1,S100A11,SCRIB,TIMELESS,TMEM184A,WDHD1 | 31 | 32 | Cell Cycle, Cellular Movement, DNA Replication, Recombination, and Repair |
| 3 | 14-3-3, ADCY5,AGRN,CDC25C,CDC6,CENPO,CHAF1A,CHAF1B,CHTF18,CLMP,CRIP2,CYRIA,DLEU2,E2f,ENO3,Histone h3,KIF20A,KNTC1,MCM10,MEIS2,NCAPG2,NSD2,NUSAP1,OIP5,PCNA,PRR11,RPS6KA3,SDSL,SETD7,SMC4,TAF10,TYMS,WDR76,ZFP36,ZWILCH | 31 | 32 | Cell Death and Survival, Cellular Assembly and Organization, DNA Replication, Recombination, and Repair |
| 4 | ATF3,AURKA,AURKAIP1,BBC3,BRCA2,C2,CDK1,CEP55,CLSPN,cytochrome C,DAB2,ERCC6L,FADS2,Gamma tubulin,H1-2,H2AX,INMT,KIF2C,KIFC1,KRT8,LIPG,MMD,PARPBP,PC,PHLDA2,PLIN4,PLK1,PPARA,RAD51,RAD54B,RAD54L,Ras homolog,RBP7,SGO2,SPON1 | 31 | 32 | Cellular Assembly and Organization, Cellular Compromise, DNA Replication, Recombination, and Repair |
| 5 | ACTG2,ADAM33,ADAMTS1,Alpha catenin,Cathepsin,CDH24,CDH6,CG,CIP2A,CLDN11,COL15A1,CRLF1,CTSV,DTL,EDN2,FGL2,FHL1,FSTL1,GGCT,GJB2,H2BC12,ICAM2,LIMCH1,MFAP5,MMP11,MSI1,NKD1,PFKFB3,S100A16,SACS,SLC12A8,SLC16A3,SLC22A18,TNFAIP2,TTN | 31 | 32 | Hereditary Disorder, Organismal Injury and Abnormalities, Skeletal and Muscular Disorders |
| 6 | AGR2,ALYREF,ATP5IF1,CAT,CRABP2,CXCL12,DDX39A,ECI1,ESM1,FOS,GSR,HADH,HMMR,Importin beta,KIF22,LAMP3,LGALS3BP,MAPK8IP2,NEIL3,NR2F6,PALLD,PFN1,PLD1,Plk,QKI,RGMA,SLPI,Sos,STX11,STXBP2,TPX2,trypsin,UGP2,UNC13D,VAMP8 | 29 | 31 | Cellular Compromise, Hematological Disease, Inflammatory Response |
| 7 | BRIP1,CCNB1,CCNB2,CENPF,CKS1B,CKS2,COMP,DEPDC1,DLGAP5,EBF1,FKBP4,FOXM1,FOXO1,GPX3,GTSE1,KIF11,KIF18A,MAP1LC3,MRPL47,MRPL55,MRPS12,MYBL1,NDN,NDUFA7,NEK2,OTUB1,OVOL1,PDK4,PEPCK,PNPLA2,Rb,RFC4,RPA,UBE2T,ZNF521 | 29 | 31 | Cellular Assembly and Organization, DNA Replication, Recombination, and Repair, Nutritional Disease |
| 8 | Alp,COL1A1,COL1A2,COX6A1,COX6B1,DENND2A,EZH1,EZH2,FBN1,Gsk3,HCAR1,IDH2,Insulin,ITGA9,LDB2,LIPE,LYPD6B,MEF2,MYH3,NDUFA3,NDUFB9,NDUFS6,NDUFS8,NR4A1,P3H2,PER1,PHKG1,PRKCA,RFXANK,RUNX2,SPINT2,SPP1,STARD10,UQCRQ,ZFPM2 | 29 | 31 | Developmental Disorder, Hereditary Disorder, Metabolic Disease |
| 9 | AKT3,ALDH1A1,arginase,ARHGAP31,ARTN,BMPR1B,Cdc2,CFD,CHL1,Cyclin B,E2F7,FOXO6,GDF11,HSPB8,IGFBP6,JUN,LAMB3,MALAT1,MAZ,MBNL1,MCTP1,MST1R,MYL9,N- | 28 | 30 | Cellular Development, Cellular Movement, Nervous |

| | | | | |
|---|---|---|---|---|
| | cor,PARD6B,PKIB,PPARG,PPP1R16B,RGL1,SGPP2,SMAD1/5/9,SMAD9,TARBP2,XYLT1,ZNF703 | | | System Development and Function |
| 10 | Actin,AHNAK,ATP6AP1,CCL28,CDK4/6,CLDN3,Cyclin E,DCTPP1,EFNA3,EFNA4,EGLN,FILIP1,FLNA,FLNC,GSN,JPT1,KPNA2,KRT15,LPP,LTBP4,NR3C1,PLS1,PPFIA4,PYGL,RNA polymerase II,SCNN1A,SDS,SGK1,SMARCA4,SORBS1,STON1,TOP2A,TPI1,UBE2C,YBX2 | 28 | 30 | Cancer, Organismal Injury and Abnormalities, Reproductive System Disease |
| 11 | ADCY4,ADGRL4,ADRB,CCNA2,CCNE1,CD36,CDCA3,CEP131,Collagen(s),DNMT1,GIMAP4,HGH1,Histone h4,IgG1,IL12 (family),KIF24,KLF2,KLF4,MEF2C,MITF,MKI67,MTBP,MYCBP2,PIK3R1,PLAGL1,RASGRP2,RGS19,S100A14,SDC1,SIAH2,SLC16A7,SNCAIP,TCF4,TFRC,TICRR | 28 | 30 | Cellular Development, Cellular Growth and Proliferation, Gene Expression |
| 12 | 26sProteasome,ADRM1,AOC3,APBA2,CNTN1,CPNE8,Creb,ELMOD3,ENPP2,FNDC1,FOLR2,GIMAP8,GSTM2,GULP1,ILF2,INHBA,INTS7,Lh,LOXL4,MYCT1,NFASC,PSENEN,PTGIS,RHOF,RNASEH2A,RORA,S100A8,S100A9,Secretase gamma,SEMA3F,SLC6A12,Smad2/3,SMPDL3B,UCHL1,VCAN | 28 | 30 | Cardiovascular Disease, Cell-To-Cell Signaling and Interaction, Inflammatory Response |
| 13 | ACKR3,ADM2,CLEC5A,Collagen Alpha1,Collagen type I (complex),COX5B,CXXC5,CYBA,CYGB,DDR2,FBLN5,FMOD,GPC3,IL11RA,IL33,Jnk,KRT10,LOXL1,MARCKSL1,MCOLN2,NFAT (complex),NOD2,OLR1,OSBP2,POSTN,POU2F3,Rbp,RIPK2,SH3BP5,SLC52A2,Sod,STRA6,TNXB,TWIST1,WDR62 | 26 | 29 | Cardiovascular System Development and Function, Cell-To-Cell Signaling and Interaction, Cellular Movement |
| 14 | CENPA,CRIP1,DLL4,ELF3,ESAM,Fcgr3,FLT4,FZD7,GADD45GIP1,Histone h2a,HJURP,IL12 (complex),LTB,LYVE1,LYZ,MEX3A,MFNG,MTHFD2,Notch,NOTCH4,PDE3B,PI3K p85,PMP22,PPM1L,PRX,PSAT1,SGCB,SLC11A1,SLC7A5,SMOOTH MUSCLE ACTIN,SNRPB,SSPN,TMEM121,TRIB3,ZNF106 | 26 | 29 | Hereditary Disorder, Neurological Disease, Organismal Injury and Abnormalities |
| 15 | Alpha tubulin,AMOTL1,AMOTL2,BETA TUBULIN,CCN1,CCT3,CD24,DNA-PK,Erm,ESRP1,ESRP2,ETS1,FN1,IGSF8,KRT19,LAMA4,LATS2,LRRK2,MCAM,MYADM,NPR1,PCGF2,PMAIP1,PP1 protein complex group,PPP1R15A,RDH10,SFRP1,SLC34A2,SLC9A3R1,TLR7/8,TPPP,TUBA1C,VGLL3,ZEB1,ZEB2 | 26 | 29 | Cancer, Cellular Movement, Organismal Injury and Abnormalities |
| 16 | ABCC6,AChR,BLM,BST2,Calmodulin,CCNE2,CYC1,DGAT2,EGR1,EPCAM,FANCA,FANCB,FANCD2,FANCI,FOSB,GPR132,HDL,HDL-cholesterol,IFI44L,IRS2,KAT2B,KCNQ1OT1,LRIG3,LRP1,NMDA Receptor,NUDT7,PDZK1IP1,RABEP2,RMI1,SPINDOC,SQLE,SWI-SNF,TSPAN2,VLDLR,XRCC2 | 26 | 29 | Cancer, Metabolic Disease, Organismal Injury and Abnormalities |
| 17 | APC (complex),ATP7B,AURKB,BUB1,BUB1B,Calcineurin A,CDC20,CDCA5,CDCA8,CENPE,COL9A2,Complement,DSN1,ERK,EYA2,GNAZ,HAS3,INCENP,ITGA1,KLF10,KNL1,L-type Calcium Channel,MAD2L1,NDC80,NUF2,protein phosphatase,RGS5,SGO1,SPC24,SPC25,TACC3,TMSB4,TRIP13,TTK,ZWINT | 24 | 28 | Cell Cycle, Cellular Assembly and Organization, DNA Replication, Recombination, and Repair |
| 18 | ACVRL1,BCAM,CD34,CDH5,CLDN5,EZR,Fc gamma receptor,Fibrin,GAS2L3,HPN,KRT17,LAMA2,LAMA5,LY6E,MECOM,MICB,Mmp,MMP9,Pgk,PLAU,PLAUR,PRSS8,PTPN14,PVT1,SCUBE3,SELL,SMAD1/5,SPDEF,SPINT1,SULF1,SYK/ZAP,TEK,TFPI,Wnt,XPR1 | 24 | 28 | Cardiovascular System Development and Function, Organismal Development, Tissue Development |

| | | | | |
|---|---|---|---|---|
| 19 | ABCB9,CALHM6,CMPK2,CYTOR,GBP5,HELZ2,IFI27,Ifn,IFN type 1,Ifnar,IL1RN,Interferon alpha,IRF7,IRF9,ISG15,ISG20,JAK1/2,KCTD12,LGALS9,MHC Class I (complex),MHC CLASS I(family),MX1,OAS1,OAS2,OAS3,OASL,PARP12,PSMB9,PSME2,RSAD2,SASH1,SLAMF8,STAT1,TAP1,TAPBP | 24 | 28 | Dermatological Diseases and Conditions, Immunological Disease, Organismal Injury and Abnormalities |
| 20 | ABCB1,ABCG2,ATAD3A,ATP13A2,cSrc,CDCP1,CSPG4,EGFR,ETV4,EXOSC4,EXOSC5,GAS1,GPRC5A,HSPBP1,HSPE1,Laminin (complex),LDL cholesterol,LMNB2,MAC,ORC1,ORC6,PDGF BB,PIP4P2,PLC gamma,PLPP3,PTK6,Rac,RECQL4,SCAMP3,SCARF1,SPART,SPRY2,UBE2S,USP2,WNT7B | 24 | 28 | Cancer, Cellular Response to Therapeutics, Organismal Injury and Abnormalities |
| 21 | ABHD6,ACO1,AKAP12,AMY2B,ANGPTL4,ARHGEF6,ATAD2,CACNG4,CEMIP,CFB,CITED2,CNIH2,CNN1,Collagen type II,CP,CPT1,CYBRD1,E2F1,E2F2,EPAS1,FAM13A,FBLN2,Ferritin,FLT1,GIPC1,GRIA,hemoglobin,KDR,RBM38,ROBO4,RRM2,SEMA6A,SMARCA2,SRC (family),Vegf | 24 | 28 | Cancer, Ophthalmic Disease, Organismal Injury and Abnormalities |
| 22 | ADAM19,Akt,Angiotensin II receptor type 1,ANGPTL2,APLNR,ARRDC3,C1QTNF6,CD93,CDON,CIB1,ECSCR,HIC1,HSPB1,JINK1/2,LAMTOR2,LAPTM4B,LINGO1,MAP3K5,MAPK10,MEG3,MT1X,NADPH oxidase,Nfat (family),NUAK1,PI3K (family),Ppp2c,PREX2,PRKD1,RAC3,SEMA6D,SERCA,SHCBP1,SPARCL1,TIE1,TMC6 | 23 | 27 | Cellular Movement, Dermatological Diseases and Conditions, Infectious Diseases |
| 23 | AMPK,APOC1,BIRC5,CEBPA,COL5A2,creatine kinase,CRYAB,FBXO32,FUT2,GOT,IKBKE,LMNB1,MTMR10,MTORC1,Nr1h,NR5A2,p70 S6k,PIGQ,PPP2R1B,PRKAA,PROCR,PTCH1,RAB25,SAMD5,SCD,SERINC1,SH3D19,SLC50A1,SPOCK2,TEAD1,TNFSF12,UCP3,VIM,VLDL-cholesterol,ZFPM1 | 23 | 27 | Cellular Response to Therapeutics, Developmental Disorder, Endocrine System Development and Function |
| 24 | ADGRF5,C1q,C1R,CALC,CALCRL,CAV2,CAVIN2,chemokine,DHCR7,DOK3,EHD2,ENPEP,EPN3,G0S2,GJC1,GPIIB-IIIA,GRK5,Hat,HMGA1,IDO1,IFI6,IFN Beta,MMP28,NFkB (complex),NFKBIE,OTUB2,PDGF-DD,RAMP1,SHARPIN,SIGIRR,TEAD,TNFRSF12A,TRAIP,TUBB6,TXNIP | 21 | 26 | Cancer, Cellular Assembly and Organization, Organismal Injury and Abnormalities |
| 25 | BOP1,CAMK1,CBX7,CCR7,CD5,Cofilin,CTSD,CXCL11,CXCL9,F Actin,FERMT2,Focal adhesion kinase,FRY,GPSM2,IFIT1,IgD,IL4I1,Integrin,KCNK5,LILRB4,LIMK1,Mek,MX2,MYLK,NFE2L3,NME1,PASK,PI3K (complex),PTPN6,RND3,Sfk,SORBS2,SPTBN2,TCR,TXNDC17 | 21 | 26 | Cancer, Hematological Disease, Organismal Injury and Abnormalities |

**Supplementary Table 4.** The top–scoring network molecule annotations, IPA Knowledge Base.

| Symbol | Entrez Gene Name | Ensembl | Expr Log Ratio | Family |
|---|---|---|---|---|
| ABRACL | ABRA C-terminal like | ENSG00000146386 | 1.30 | other |
| ASPM | assembly factor for spindle microtubules | ENSG00000066279 | 2.98 | other |
| B4GALT3 | beta-1,4-galactosyltransferase 3 | ENSG00000158850 | 0.94 | enzyme |
| CDC14B | cell division cycle 14B | ENSG00000081377 | -1.39 | phosphatase |
| CDC42EP3 | CDC42 effector protein 3 | ENSG00000163171 | -1.22 | other |
| CDCA2 | cell division cycle associated 2 | ENSG00000184661 | 2.07 | other |
| CDCA7 | cell division cycle associated 7 | ENSG00000144354 | 1.02 | other |
| CDKN3 | cyclin dependent kinase inhibitor 3 | ENSG00000100526 | 2.267 | phosphatase |
| CHST3 | carbohydrate sulfotransferase 3 | ENSG00000122863 | -1.47 | enzyme |
| EPSTI1 | epithelial stromal interaction 1 | ENSG00000133106 | 0.72 | other |
| ERBB2 | erb-b2 receptor tyrosine kinase 2 | ENSG00000141736 | 0.84 | kinase |
| ESPL1 | extra spindle pole bodies like 1,separase | ENSG00000135476 | 2.69 | peptidase |
| GBE1 | 1,4-alpha-glucan branching enzyme 1 | ENSG00000114480 | -1.02 | enzyme |
| GINS1 | GINS complex subunit 1 | ENSG00000101003 | 2.14 | other |
| HEPH | hephaestin | ENSG00000089472 | -1.23 | transporter |
| HSD17B11 | hydroxysteroid 17-beta dehydrogenase 11 | ENSG00000198189 | -1.22 | enzyme |
| LIG1 | DNA ligase 1 | ENSG00000105486 | 1.06 | enzyme |
| MFAP2 | microfibril associated protein 2 | ENSG00000117122 | 1.92 | other |
| MXD3 | MAX dimerization protein 3 | ENSG00000213347 | 1.43 | transcription regulator |
| MYO10 | myosin X | ENSG00000145555 | 0.73 | enzyme |
| NCAPD2 | non-SMC condensin I complex subunit D2 | ENSG00000010292 | 0.90 | other |
| NCAPG | non-SMC condensin I complex subunit G | ENSG00000109805 | 2.57 | other |
| NCAPH | non-SMC condensin I complex subunit H | ENSG00000121152 | 2.48 | other |
| NUP210 | nucleoporin 210 | ENSG00000132182 | 2.06 | transporter |

| | | | | |
|---|---|---|---|---|
| PHLDB1 | pleckstrin homology like domain family B member 1 | ENSG00000019144 | -1.12 | other |
| PLAAT4 | phospholipase A and acyltransferase 4 | ENSG00000133321 | 0.57 | enzyme |
| POLE2 | DNA polymerase epsilon 2. accessory subunit | ENSG00000100479 | 1.11 | enzyme |
| POLQ | DNA polymerase theta | ENSG00000051341 | 2.00 | enzyme |
| POLR3K | RNA polymerase III subunit K | ENSG00000161980 | 0.99 | transcriptio n regulator |
| PORCN | porcupine O-acyltransferase | ENSG00000102312 | 0.54 | enzyme |
| RAD51AP 1 | RAD51 associated protein 1 | ENSG00000111247 | 1.85 | other |
| RFC2 | replication factor C subunit 2 | ENSG00000049541 | 0.92 | other |
| SMTN | smoothelin | ENSG00000183963 | -0.92 | other |
| TSPAN13 | tetraspanin 13 | ENSG00000106537 | 0.63 | other |
| UNC5B | unc-5 netrin receptor B | ENSG00000107731 | 1.34 | transmembr ane receptor |
